# White light employing luminescent engineered large (mega) Stokes shift molecules: a review

**DOI:** 10.1039/d1ra00129a

**Published:** 2021-04-12

**Authors:** Nadia Nabihah Mohd Yusof Chan, Azila Idris, Zul Hazrin Zainal Abidin, Hairul Anuar Tajuddin, Zanariah Abdullah

**Affiliations:** Department of Chemistry, Faculty of Science, University of Malaya 50603 Kuala Lumpur Malaysia azila_idris@um.edu.my; Centre for Ionics University of Malaya, Department of Physics, Faculty of Science, University of Malaya 50603 Kuala Lumpur Malaysia

## Abstract

Large (mega) Stokes shift molecules have shown great potential in white light emission for optoelectronic applications, such as flat panel display technology, light-emitting diodes, photosensitizers, molecular probes, cellular and bioimaging, and other applications. This review aims to summarize recent developments of white light generation that incorporate a large Stokes shift component, key approaches to designing large Stokes shift molecules, perspectives on future opportunities, and remaining challenges confronting this emerging research field. After a brief introduction of feasible pathways in generating white light, exemplifications of large Stokes shift molecules as white light candidates from organic and inorganic-based materials are illustrated. Various possible ways to design such molecules have been revealed by integrating the photophysical mechanisms that are essential to produce red-shifted emission upon photoexcitation, such as excited state intramolecular proton transfer (ESIPT), intramolecular charge transfer (ICT), excited state geometrical relaxation or structural deformation, aggregation-induced emission (AIE) alongside the different formations of aggregates, interplay between monomer and excimer emission, host–guest interaction, and lastly metal to ligand charge transfer (MLCT) *via* harvesting triplet state. Furthermore, previously reported fluorescent materials are described and categorized based on luminescence behaviors on account of the Stokes shifts value. This review will serve as a rationalized introduction and reference for researchers who are interested in exploring large or mega Stokes shift molecules, and will motivate new strategies along with instigation of persistent efforts in this prominent subject area with great avenues.

## Abstract 2

Illustration of white light designated with the 1931-CIE coordinate of (0.33, 0.33), and photophysical mechanisms that contribute to large Stoke shift molecules.

## Graphical abstract

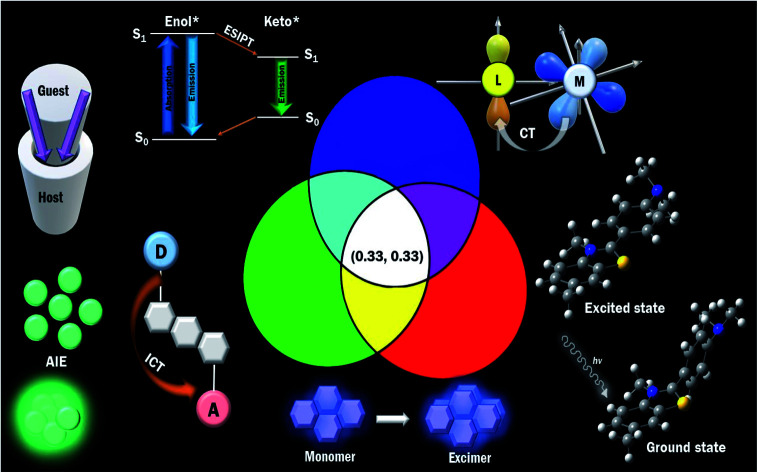

## 1. Introduction

**Fig. 1:**
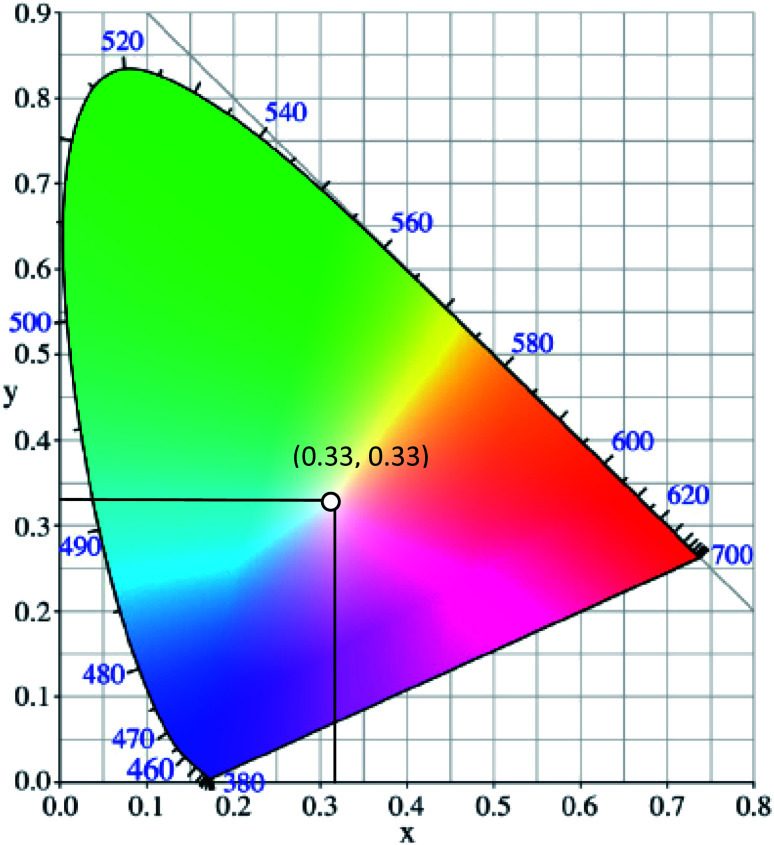
The chromaticity coordinates (*x*, *y*) of pure white light, as given by the Commission Internationale d'Eclairage, is (0.33, 0.33). The figure was adapted from ref. 25 with accreditation given to Creative Commons Attribution 4.0 International Public License.

White light can be generated from the combination of the whole visible spectrum of electromagnetic radiation. It can thus appear as broadband or multi-band emission. Physical means to generate white light may involve the combination of a few specific kinds of light, such as compact and discrete red-green-blue (RGB) light-emitting diodes (LEDs), single phosphor based white LED,^1^ uniform photonic crystal fiber using microchip laser,^2^ lanthanides-doped film by pulsed laser ablation^3^ or through the fabrication of multiphase phosphors on an ultraviolet-emitting diode.^4^ Based on the former pathway, the generation of white light resulted from the process of color additive mixing. Upon additive mixing, the color rendering has been evaluated based on the physiologically perceived colors by humans, known as the Commission Internationale d’Eclairage (CIE)-1931 chromaticity diagram.^5^ A pure and ideal white light is designated as (0.33, 0.33) in CIE coordinates (Fig. 1).^6^ In the last century, tremendous advancements of white light generation have been witnessed through varying methods and materials. Methods ranged from an attempt for a complementary mixing of colors (blue and yellow or cyan and orange), assembling three primary colors (RGB) in a device, while operating each of them independently, to the utilization of blue or UV-LED to excite phosphor materials by means of energy conversion. The most remarkable method to achieve a true white light is reflected upon the correct ratio mixture of three primary colors.^7^ In view of this, researchers developed a single white emissive molecule,^8^ or combined molecules with different emissive wavelengths in a single mixture.^9,10^ One of the unwelcomed natures when mixing different molecules, however, is the Förster Resonance Energy Transfer (FRET) effect. The FRET effect quenched the emission intensity; thus, curtailing the efficiency and brightness of the light.^11^ Looking from the perspective of white light emitting materials, the photophysical mechanisms need to be scrutinized in order to understand the possible pathways of suitable chromophores designation.^12^ A detailed understanding of these mechanisms prompted not only a true white light in the future, but also great photostability, tunability and quantum efficiencies.

The spectral distributions and quantum efficiencies of chromophores are one of the many crucial attributes to be assessed when considering a larger scale material production. It is without doubt that previous research strides have been focused on molecules engineered with high quantum efficiencies in their respective emission wavelength region.^13–15^ High quantum efficiency materials are a desirable characteristic in most imaging applications for the best display, illumination, and performance. In white light application, several efforts to design suitable chromophores have been done by different research groups, employing organic,^9^ inorganic,^16^ supramolecular chemistry,^17^ polymer micelle,^18,19^ biocomposite,^7^ lanthanides doped phosphors,^3,20^ quantum dot,^21^ carbon dot,^22,23^ and other approaches. Continuous efforts in experimenting with different types of white light emitting materials were inspired by the demand for cost effective production, usage of low toxicity materials, scarcity of resources that make up current materials, facile fabrication, green strategy and existing loopholes in generating a true white light.^24^ The discussion in this review article highlights the recent developments of employing large Stokes shift materials to generate white light, diverse classes of organic and inorganic materials with large Stokes shift, and molecular engineering approaches to large Stokes shift molecules with relevant applicability to optoelectronic devices. Practically, a large or mega Stokes shift is a desired feature for fluorophores in white light emission to inhibit self-absorption or the inner filter effect.

## 2. Employing large Stokes shift molecules in white light emission

An ideal white light emitter requires the simultaneous emission of red, green, and blue with almost similar intensity distribution to cover the entire region of the visible spectrum. On this basis, research groups of vastly different expertise have addressed the fundamental question to fulfill this condition by developing a single white emitter or mixing different emitters that originate from a single excitation wavelength. Through this approach, there will be no or minimal emission reabsorption that would quench the overall white intensity and efficiency. Nevertheless, such strategy is undeniably challenging and requires future extensive study to gain comprehension of the multicomponent mixture behaviors. The realization of a material with multicomponent emitters is expected by connecting them *via* covalent bonds or noncovalent interactions.^26^ This includes hydrogen bonds, van der Waals forces, π–π stacking, dipole-induced dipole forces, metal coordination, and others. In order to achieve this, employing a large Stokes shift molecule within the selection of material provides a state-of-the-art strategy that becomes of paramount importance in the field of optoelectronics, bio-imaging, next-generation display and sensing devices.

A Stokes shift is the difference in energy between the band maximum of the absorption and the lower energy emission. It is a key parameter of the luminescence property because its value quantifies and explains other significant parameters of the studied compounds, for instance, how the Stokes shift justifies the different dipole moments of molecules in different electronic states and how changes in the solvent polarity affects the polarization and anisotropy of fluorescence.^27^ The relationship between the solvent polarity parameter and Stokes shift can be evaluated quantitatively according to the Lippert–Mataga equation below .^28^ Δν corresponds to the Stokes shift value (cm^−1^), while *υ*_a_ and *υ*_e_ are the absorption and emission wavenumbers, respectively. The letter *h* is Planck's constant, *c* is the speed of light, and *a* is the Onsager radius. Parameters *μ*_e_ and *μ*_g_ are the permanent dipole moments of the excited state and ground state, respectively, in which values can be obtained from the slope of a Lippert–Mataga plot. *ε*_0_ corresponds to the permittivity in a vacuum, ε is the static dielectric constant of the solvent, *n* is the refractive index and Δ*f* represents the orientational polarizability. This plot has been reported by researchers when studying the Stokes shift parameter of molecules.^29–31^1
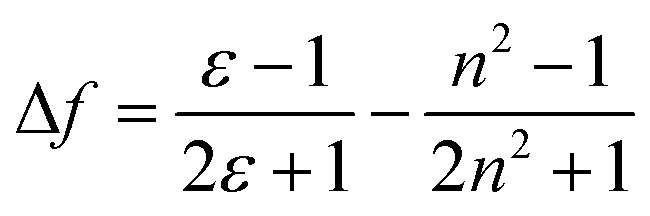
2
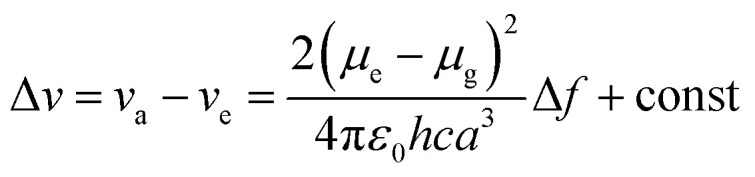


**Fig. 2:**
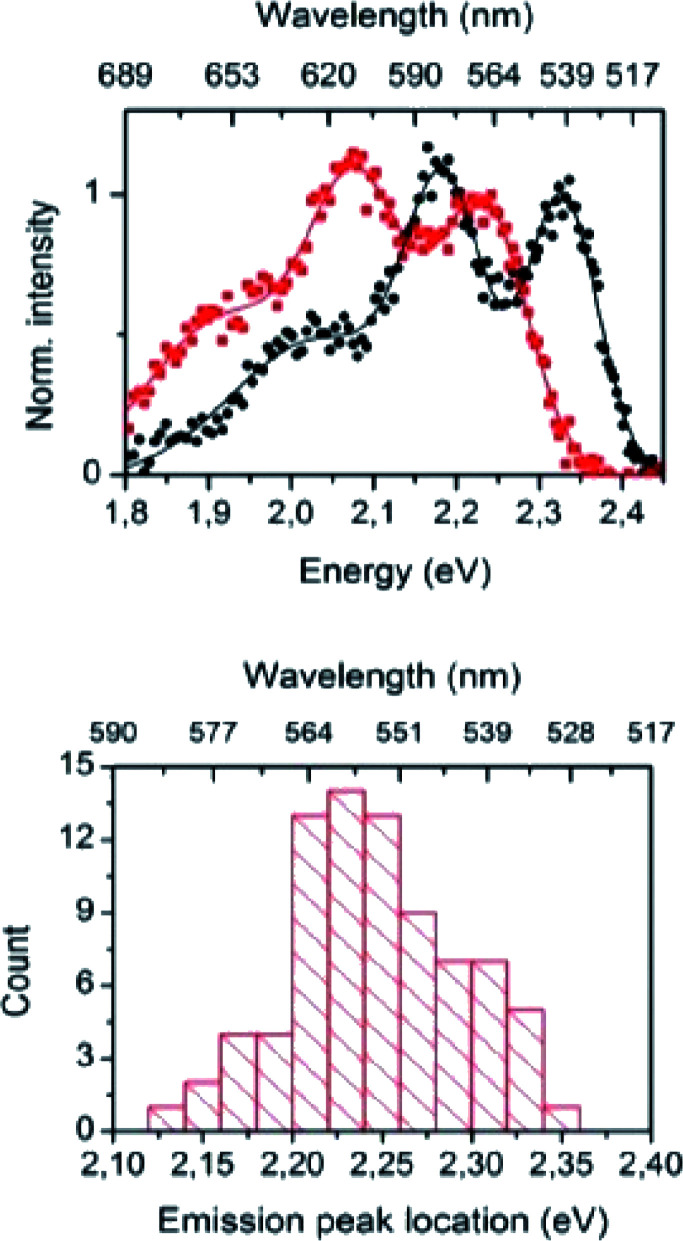
(Top) Examples of the recorded emission spectra from different emitters, indicating molecule-to-molecule differences in the shape and the spectra position. Solid lines are a guide to the eye, and based on double and triple Gaussian fits to the data. (Bottom) The histogram for the emission shows the distribution of the peak positions. The figure was adapted from ref. 32 with permission. Copyright 2014 American Chemical Society.

The importance of the Stokes shift in the phenomena of spectral diffusion and spectral heterogeneity at room temperature has been demonstrated by Stopel and his co-workers in their single organic molecule study.^32^ The Stokes shift has always been assumed to be constant for single molecules. However, in reality, heterogeneity in the conformation of chromophores and their nanoenvironments can determine the exact photophysical quantities, including the lifetime, polarization and Stokes shift variation. In Stopel *et al.*'s study, the polystyrene-embedded perylene derivative *N*,*N*-di(*tert*-butoxycarbonyl)-9-amino-*N*-(2,6-diisopropylphenyl)perylene-3,4-dicarboximide showed a Stokes shift of 96 meV for the ensemble toluene solution. Meanwhile, for the single molecule analysis, they recorded 80 emission spectra to construct a histogram of the highest energy emission peak energies (Fig. 2). The broad width distribution (95 meV) of the histogram implies inconsistency of the Stokes shift for different molecules, reflecting differences in the polarity and flexibility of the individual nanoenvironments that directly affect the Stokes shift. This study has initiated a promising tool to study the correlation of the energy band gap between the ground state and excited state, besides quantifying the local variations of the chromophores in matrix-like polymers. Future experiments involving single organic dyes in solvents of varying polarity may be conducted to establish a detailed characterization of the Stokes shift.

### 2.1 Organic molecules

After having some insights on the importance of the Stokes shift, the next aspect to consider is the challenges in the synthesis and molecular designation of large Stokes shift materials. Herein, several organic-based molecules that were reported to exhibit a large Stokes shift are to be discussed. There are huge classes of organic compounds that exhibit a large Stokes shift, such as terpyridines, pyrenes, BODIPY, coumarins, benzothiazoles, quinolines, quinoxalines, and others. Studies revolving around pure organic molecules as emitters started when researchers aspired for a greener approach and low cost production as a replacement for metals, inorganic or biological hybrids. In this section, each structural framework will be reviewed in terms of their photoluminescence properties and potential applicability in white light emission. A hybrid fluorophore based on terpyridine-benzoxadiazole (TPBD) was developed by Yang *et al.*, which enlarged the donor–π–acceptor system, leading to a large Stokes shift (127 nm).^33^ In an acidic environment (pH = 2), the absorption wavelength red-shifted from 470 nm to 494 nm. This was due to the protonation of two lateral pyridine nitrogen atoms, which enhanced the electron-withdrawing ability of the terpyridine moiety. Resultantly, TPBD also emitted at longer wavelengths up to 624 nm. Another terpyridine-based fluorescent material has been investigated by Song *et al.*, which highlighted the relatively large Stokes shift, quantum yield and strong solvatochromism properties.^34^ Such characteristics were achieved by integrating an acceptor moiety (*N*,*N*-diphenylaminophenyl aldehyde) into the terpyridine system, which served as the donor moiety (with electrons coming from nitrogens), thus conferring an efficient charge transfer in the overall molecule. In alcoholic solvents, this derivative emitted at long wavelengths (*ca.*, 505–524 nm), which was detected to be due to intramolecular charge transfer (ICT) in the excited states and the twisting dynamic of the terpyridine unit. Several achievements incorporating terpyridine motifs in white emission include co-polymerization with a polyacrylamide network in a hydrogel^35^ and the formation of a supramolecular polymer gel with pyridinium salt units to emit strong white light at ∼474 nm and 571 nm.^36^

One of the highly acclaimed fluorescent dyes is the pyrene chromophore. Due to its ability for strong π–π interaction, a pyrene monomer can form an excimer (excited state dimer) species. In 2017, a large Stokes shift and bright red-emitting pyrene was developed by introducing a π–acceptor system.^37^ The pyrene was coupled with a pyridinium moiety to establish a strong push–pull effect with concentrated electrons coming from the highly conjugated pyrene. The compound successfully emitted light at 610 nm with a Stokes shift of up to 130 nm, making it biologically useful for cell staining. An extension of the acceptor strength was pursued by Chen *et al.* in 2020, whereby a boronic moiety was engulfed to further extend the acceptor fragment.^38^ Interestingly, a mega Stokes shift of up to 220 nm was observed as the compound bound to detect hydrogen peroxide, thereby reducing crosstalk between the excitation (*λ*_ex_ = 380 nm) and emission light. Chao and co-workers designed a pyrene-based colorimetric and fluorescent pH probe with a large Stokes shift of up to 145 nm.^39^ 1-Acetylpyrene was mixed with 2-aminopyridine-3-carbaldehyde in anhydrous ethanol to afford 2-pyrenyl-1,8-naphthyridine (PNY). PNY initially showed two absorption bands at 271 nm and 368 nm. Upon acidification, the absorbance at 271 nm increased, and an opposite trend was observed for the 368 nm band. In excess acidic condition, new peaks at 322 nm, 338 nm and 429 nm appeared and gradually increased, which happened concomitantly with a distinct color change from colorless to yellow. The fluorescence wavelength was observed at 515 nm, which prevented self-quenching of the dye. These significant characteristics prompted future study of the pyrene molecules in display, sensing and imaging applications.

**Fig. 3:**
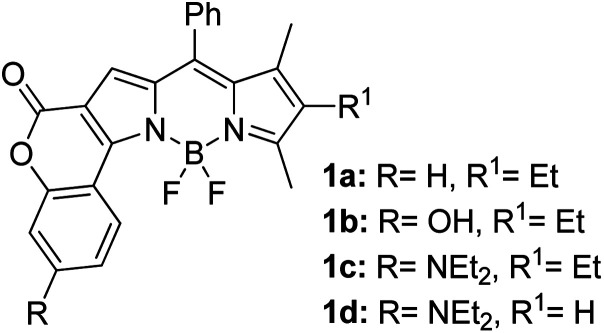
Diethylamino-substituted dyes show large Stokes shifts in polar solvents (MeOH and DMF). The compounds are from ref. 40.

Another notable fluorescent dye that is capable of emitting light in the orange to red region (500–600 nm) of the visible spectrum is the classical 4,4-difluoro-4-bora-3*a*,4*a*-diaza-*s*-indacene (BODIPY) based dye. In 2013, novel non-symmetrical coumarin-fused BODIPY dyes were discovered by Bochkov and his group.^40^ This series of dyes (Fig. 3) were reported to show near-infrared (NIR) emission with an exceptionally large Stokes shift and good fluorescence quantum yield. Compound 1a has a narrow Stokes shift of 30–40 nm in different types of solvents because of the non-substituted coumarin that is fused to BODIPY, while compound 1b is more promising, having a Stokes shift of almost 60 nm in DMF. However, when 7-diethylamino-4-hydroxycoumarin is fused to BODIPY, a dramatic bathochromic shift of the excitation and emission bands was observed, reaching up to 144 nm Stokes shift value in a similar solvent. This is the highest ever reported Stokes shift for BODIPY derivatives to the best of our knowledge. This phenomenon can be associated with the strong ICT character of the coumarin and BODIPY in the excited state. Typically, emissive states with ICT feature are weakly fluorescent in polar solvents compared to that in non-polar solvents. This corroborates with results observed in Bochkov *et al.*‘s study, where the fluorescence quantum yields of 1b were 0.71 and 0.65 in toluene and methanol, respectively. Quenching was detected in a polar aprotic solvent (DMF) with *Φ*_F_ = 0.15. Meanwhile, a large disparity was detected for 1c when tested in non-polar and polar solvents. The diethylamino-substitution on the coumarin moiety produced *Φ*_F_ = 0.86 and *Φ*_F_ = 0.62 in cyclohexane and toluene, respectively, while strong quenching occurred in methanol (*Φ*_F_ = 0.06) and DMF (*Φ*_F_ = 0.04). It was understood that the formation of twisted ICT (TICT) caused a full charge separation in polar media (a positively charged dialkylamino group rotated perpendicularly to the plane of the molecule). The TICT excited states resulted in a considerably high percentage of the non-radiative decay pathway. The incorporation of BODIPY dyes in stride for white light emission has been recently demonstrated through the supramolecular assembly of three organic components emitting at different wavelength regions.^41^

**Fig. 4:**
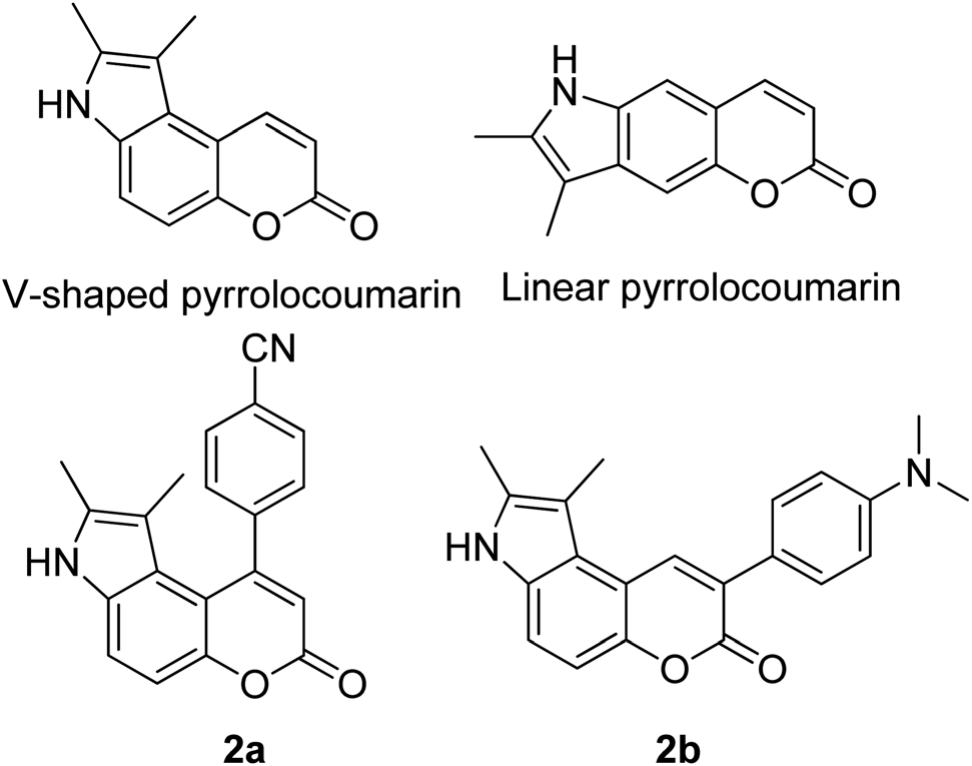
Pyrrolocoumarin derivatives with chemical modification. The compounds are from ref. 43.

Many coumarin dyes exhibit large Stokes shifts and fulfill the requirements for use in bio-imaging and optoelectronics. Generally, neutral, compact, and medium polarity compounds (several heteroatoms as hydrogen bond donors and acceptors) are suitable to cross the membranes of living cells. For instance, the mega Stokes shift dyes from Dyomics are coumarins absorbing around 500–520 nm and emitting in the region of 590–670 nm in ethanolic solvent.^42^ Emitting at much lower energy (>600 nm) proffers less scattering and safer biochemical communication in the human body. There are many studies in the literature presenting large or mega Stokes shifts for coumarin derivatives. Chen *et al.* reported new pyrrolocoumarin derivatives with remarkably large Stokes shifts. Aiming for coumarin as the parent skeleton, their synthetic route proceeded through Fischer's indole synthesis and Suzuki coupling to form either V-shaped or linear molecules (Fig. 4).^43^ The highlight of their discovery is compound 2b, which was reported to exhibit a Stokes shift of 113 nm and long emission wavelength at 523 nm, producing an intense green fluorescence (*Φ*_F_ = 0.55). This provides potential applicability of this fluorescent compound in biological FRET devices, with more efficient energy transfer. Compound 2a has a Stokes shift of 159 nm, which is reported to be the highest among the series of derivatives that they synthesized, and is related to the presence of a strong electron withdrawing cyano-group within the molecule. Nevertheless, a large Stokes shift always happens collectively with a non-radiative decay pathway. In their study, 2a exhibits a low fluorescence quantum yield of 0.03. A more detailed exploration on the photophysical nature of coumarin chromophores upon attaching different substituents was evaluated by Liu *et al.* Apparently, attaching an electron-withdrawing group at the 4-position of coumarin will greatly stabilize the LUMO, and it is thus able to reduce the energy band gap between the HOMO and LUMO. This is due to the higher atomic contribution to the aforementioned position, as compared to the rest of the positions available in a coumarin structure.^44^ The discovery by Liu and co-workers consolidates with the red-shifted emission wavelength observed in 2a (*λ*_em_ = 547 nm) compared to that in 2b (*λ*_em_ = 523 nm). Integrals between coumarin and naphthalimide were reported to nearly construct an ideal white light with a CIE coordinate of (0.29, 0.33) in the solid form.^45^ Furthermore, tunable fluorescence derived from the host–guest system of perylene bis(diimide) (PDI) and coumarin has been recently reported, successfully covering the whole white light region with a CIE coordinate of (0.32, 0.35) upon excitation at 360 nm. Jin and his research group developed a host-induced white emission for the mixture of coumarin and PDI using the macrocycle cucurbit[8]uril to boost the fluorescence intensity and efficiency.^46^ These are clear examples of coumarin derivatives that may be useful in white-emitting applications.

**Fig. 5:**
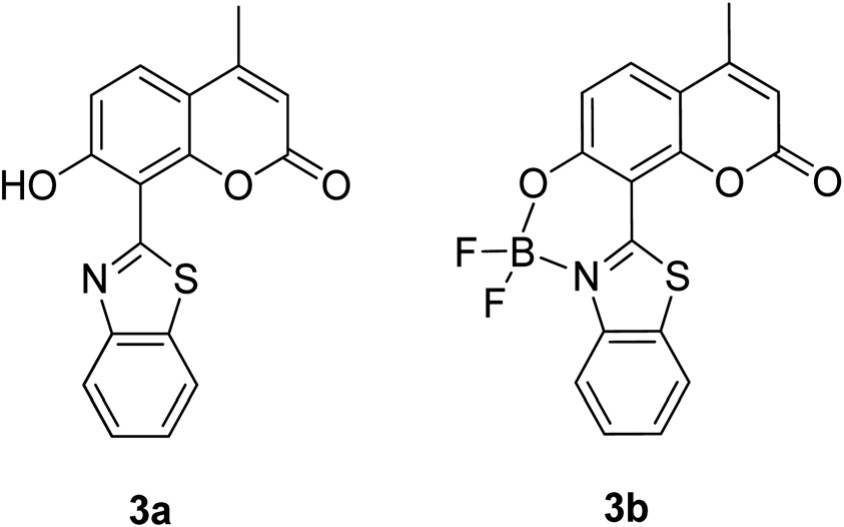
Dual-emissive coumarin-based dyes with 8-benzothiazole (left) and its difluoroboron bound derivative (right). The compounds are from ref. 51.

The screening of large Stokes shift compounds can be particularized when evaluating candidates, especially from physiologically withstanding material like in bioimaging and molecular probe applications. Benzoxazole, benzothiazole and benzimidazole molecules are renowned to be utilized in such applications due to their salient photophysical characteristics, such as dual-emission, ease of synthetic modification and good photostability.^47–49^ Gao *et al.* synthesized a fluorescent dye, 2,5-bis(6-amine-benzoxazol-2-yl)thiophene (BBTA) that possessed strong emission at 568 nm with the largest Stokes shift of 186 nm^48^ when tested in buffer solution (phosphate-buffer saline (PBS), pH = 7.2) with 5% (v/v) of polyethylene glycol monomethyl ether as an additive. This additive increased the aqueous solubility of BBTA and caused the molecules to aggregate in the ground state, which slightly shifted the maximum absorption of the fluorophore towards the UV range at around 382 nm. The result depicts a distinct gap between the maximum absorption and emission wavelength that consequentially gave rise to an exceptionally large Stokes shift. BBTA was also reported to exhibit excellent stability over a wide pH range and excellent photostability, which makes it a feasible material in lighting applications. Zhao and co-workers developed a ratiometric fluorescence probe with large Stokes shift for the detection of Cu^2+^ based on a new clamp-on unit.^50^ The molecular design is based on *ortho*-arylethylnyl benzothiazole, an aromatic alkyne that exhibits a maximum absorption wavelength at 375 nm and maximum emission wavelength at 566 nm (Stokes shift of 191 nm). The notion of this observation is the presence of a benzothiazole unit that promotes ESIPT within the molecule. Upon coordinating with Cu^2+^, the emission wavelength blue-shifted to around 446 nm because of the “push–pull” structure that draws the electron density from the triple bond towards the copper ion. This induced high selectivity of the designed compound towards Cu^2+^, making it a good indicator as a probe. Recently, Xie and his co-workers synthesized two dual-emissive fluorescent dyes based on the coumarin core and benzothiazole moiety.^51^ Both derivatives (3a and 3b in Fig. 5) showed a notably large Stokes shift of up to 188 nm in toluene. The Stokes shift for the derivatives are relatively large compared to most of the coumarin-benzothiazole fluorophores that have ever been reported. The multi-emission properties of these compounds become particularly interesting for white light electroluminescence application because of the ability to circumvent the molecular self-absorption problem that disrupts the white light intensity and color purity. From the Xie group analysis using the time-dependent density functional theory (TDDFT) method, it was found that there is a redistribution of the frontier molecular orbitals at the excited states of the compound after absorbing light. Existing as an enol form in the ground state (S_0_) and keto form in the excited state (S_1_), the molecular orbitals of compound 3a in the latter state is simulated to be more localized, creating a small energy band gap between its HOMO and LUMO that can rationalize the large Stokes shift event. The difluoroboron bound derivative (3b) produced dual emission even with the absence of the enol rotamer structure, which confirmed that the large Stokes shift can occur upon reallocation of the molecular orbitals.

**Fig. 6:**
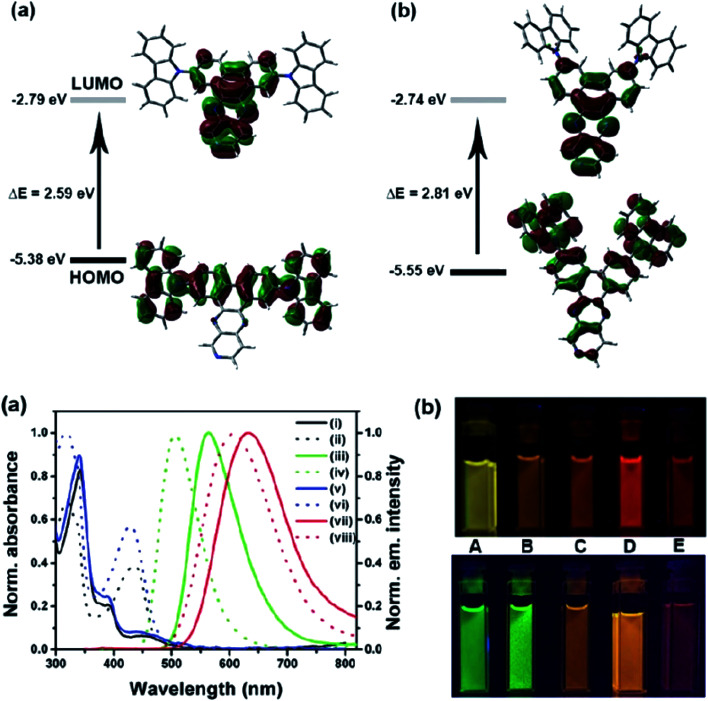
(Top) The optimized ground-state HOMO and LUMO distributions, and the respective energy values of (a) PQCz-T and (b) PQCz-V obtained by DFT calculations at the B3LYP/6-31G(d,p) level. Bottom: (a) normalized absorption and emission (*λ*_ex_ = 340 nm) spectra of PQCz-T (solid line) and PQCz-V (dotted line) in toluene (absorption: black), emission: green, (i–iv) and CH_2_Cl_2_ (absorption: blue), emission: red, (v–viii). (b) The digital photographs of PQCz-T (upper panel) and PQCz-V (bottom panel) in solvents of varying polarity under the illumination of a UV lamp (*λ*_ex_ = 365 nm). (A) Toluene (0.099), (B) 1,4-dioxane (0.164), (C) tetrahydrofuran (0.207), (D) chloroform (0.259) and (E) dichloromethane (0.309). The solvent polarity parameter (*E*^N^_T_ value) is mentioned in the parenthesis. The figure was reproduced from ref. 55 with copyright permission from The Royal Society of Chemistry.

Another attractive organic scaffold that exhibits a large Stokes shift is the quinoline unit. This privileged structure has been widely used as a core molecular fragment in pharmaceuticals, biocompatible substance, stimulated emission depletion (STED) microscopy, and FRET imaging.^52^ False-positive outcomes from overlapping in the emission and excitation spectra of the donor and acceptor molecule, respectively, can be resolved by integrating a large Stokes shift dye.^52,53^ The provision of such integral would allow scientists to perform robust intermolecular FRET assays to investigate the molecular interactions. Styrylquinolines developed by Czaplińska and co-workers displayed a remarkable push–pull capability.^52^ Derivatives bearing strong electron-donating substituents produced a markedly enhanced extinction coefficient of the low energy band and exceptionally large Stokes shifts in ethanol (131–152 nm). Furthermore, the influence of the bithienyl and *N*-octyl-carbazyl groups on quinolines has been extensively investigated by the same group, showing distinct photophysical characteristics.^31^ In Czaplińska *et al.*'s work, it was found that *N*-octyl-carbazole moieties provided a better quantum yield (*Φ*_em_ = 9–70%) and longer lifetime (*τ* = 0.83–5.72 ns) as compared to the bithiophene moieties. An enlarged Stokes shift was realized in a quinoline-*N*-octyl-carbazole system that contained a strong electron-withdrawing nitro (NO_2_) group (*λ*_em_ > 600 nm). It was understood that the NO_2_ facilitates relaxation processes by intensifying the acceptor nature of the quinoline unit. Quinolines are capable of upholding ICT due to its electron-accepting nature from the well-defined dipole moment. Hisham *et al.* reported a large Stokes shift in the range of 93–120 nm for *N*-aryl-2-aminoquinolines bearing strong electron-donating substituents (CH_3_, CH_2_CH_3_, OCH_3_, OH) at the para position on the aryl moiety.^54^ This rationalized the delocalization of electrons from the aryl moiety into the quinoline ring, which strongly withdraws electrons. Closely related nitrogen-bearing heteroaromatic frameworks that were studied for their large Stokes shift include derivatives of quinoxaline and pyridine. Interestingly, a T and V-shaped donor–acceptor–donor (D–A–D) structure in the context of a single molecular material has been explored from the combination of these heteroaromatics. In 2018, Sk and his research group integrated a pyridoquinoxaline skeleton as the acceptor core, linked to carbazole groups as the electron-donating groups with the provision that they have excellent electronic properties, high thermal stability, and low cost.^55^ A single-component white light emission was observed, including the temperature-induced tunable emission from blue to orange region of the visible spectrum. In solvents of varying polarity, the compound showed an absorption peak at around 340 nm with shoulders at 390 and 440 nm. However, the emission spectra were recorded at *λ*_em_ = 564 nm (yellow fluorescence) in toluene, which gradually red-shifted to *λ*_em_ = 631 nm (red fluorescence) in the more polar dichloromethane (refer Fig. 6). This gives rise to the solvent-dependent high Stokes shift of the compound, which agrees with the facile ICT and possible twisted ICT (TICT) state due to the effortless D-A rotation. Xu *et al.* reported surprisingly high external quantum efficiencies (EQE) for bicyclic fused pyrazine of up to 7.37%, which justifies that the nitrogen-bearing main chromophores could potentially be applied in optoelectronic and light-emitting diode applications in the future.^56^ The theoretical limit EQE for the fluorescent emitter is only 5%, thereby opening versatile avenues of nitrogen-fused aromatics.

**Fig. 7:**
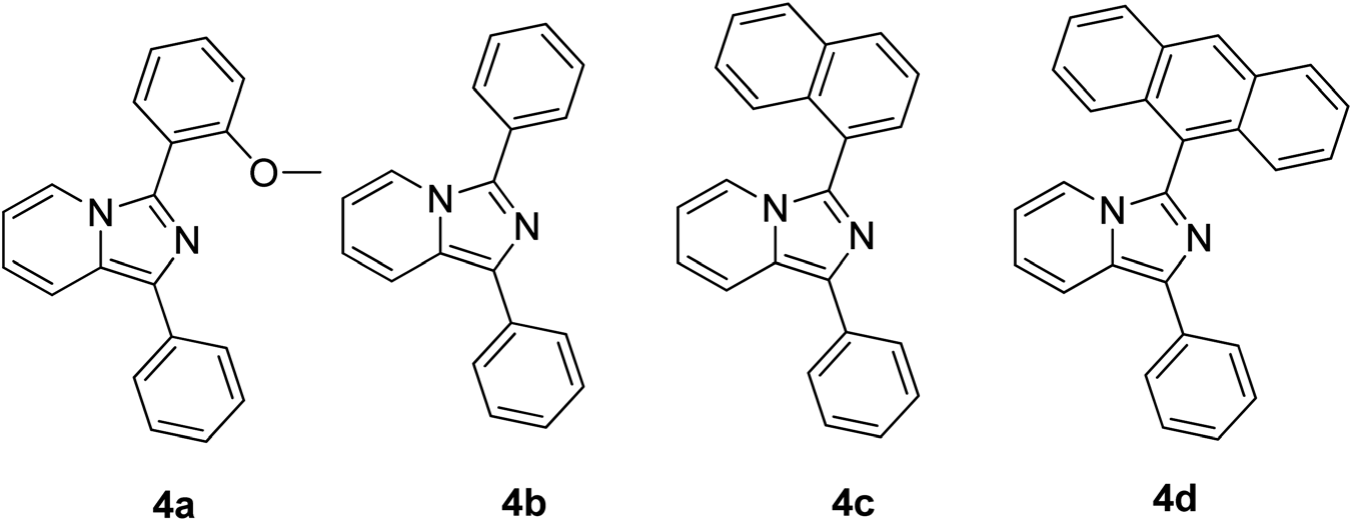
Different substituents in position 3 of the 1-phenylimidazo[1,5-*a*]pyridine skeleton. The compounds are from ref. 57.

Continuous effort in searching for a low-cost emitter with a large Stokes shift was realized by Volpi *et al.* when synthesizing a series of 1,3-diarylated imidazo[1,5-*a*]pyridine derivatives.^57^ We selected several of their compounds (Fig. 7) to discuss the absorption and emission properties. Compound 4a has a Stokes shift of 174 nm and high fluorescence quantum yield, *Φ*_F_ = 0.385, as compared to the rest of the molecules. Thus, it was selected to test as a luminescent low-cost material candidate for downshifting application. The compound was uniformly dissolved in a transparent thermosetting polyurethane resin in a 1 : 1 ratio to produce solid fluorescence. The absorption 4a in the solid state and in dichloromethane solution showed no changes, while the emission in the solid state was blue-shifted by about 15 nm as compared to the solution. From compound 4b to 4d, the extension of the conjugation in position 3 showed bathochromic shift emission wavelengths in the region of 460 nm–550 nm (cyan color). The presence of an anthracene moiety in 4d has shown a quite large Stokes shift value of 166 nm. However, to a slight dismay, a lower yield was observed with *Φ*_F_ = 0.064.

A low molecular weight material with a single aromatic ring has been highlighted in a study conducted by Percino and his group.^58^ They introduced two electron-withdrawing cyano groups in 4-((2-hydroxyethyl)(methyl)-amino)benzaldehyde, and compared its luminescence property with the commercial polymer poly(2-methoxy-5(2′-ethyl)hexoxy phenylenevinylene) (MEH-PPV). Compellingly, their compound showed red emission, which red-shifted slightly by 67 nm when compared to MEH-PPV. Both molecules possessed notable capacity to form a thin film with smooth morphology, thereby making it high likely useful as an OLED. In methanol, the compound 2-(4-((2-hydroxyethyl) (methyl)amino)benzylidene)malononitrile produced a large Stokes shift of more than 150 nm, which was due to intra- and intermolecular interactions with solvent molecules. Another work showing a potential organic molecule as a light-emitting diode includes the designation of 5,5′-([1,2,5]thiadiazolo[3,4-*c*]pyridine-4,7-diyl)bis(*N,N*-diphenylthiophen-2-amine) (DTPS-PT), which generated real NIR emission with *λ*_onset_ > 700 nm in both doped and nondoped devices.^59^ Gratifyingly, the molecule was able to achieve a maximum radiance of 2202 mW Sr^−1^ m^−2^, which is known to be one of the highest values ever reported for NIR-OLEDs. The central pyridine thiadiazole (PT) worked as an acceptor against the donor diphenylamine thiophene (TPS). These examples depicted a wide selection of organic molecules that exhibit large or mega Stokes shift property and outstanding luminescence responses, which make them highly sought after as low-cost alternatives to the existing metal-based or inorganic emitters.^60^ Nevertheless, a deeper understanding of the processes for different chromophores is crucial in order to modify the current molecular structure to achieve better optical features. The details of these mechanisms will be discussed in Sections 3.1–3.7.

### 2.2 Inorganic molecules

**Fig. 8:**
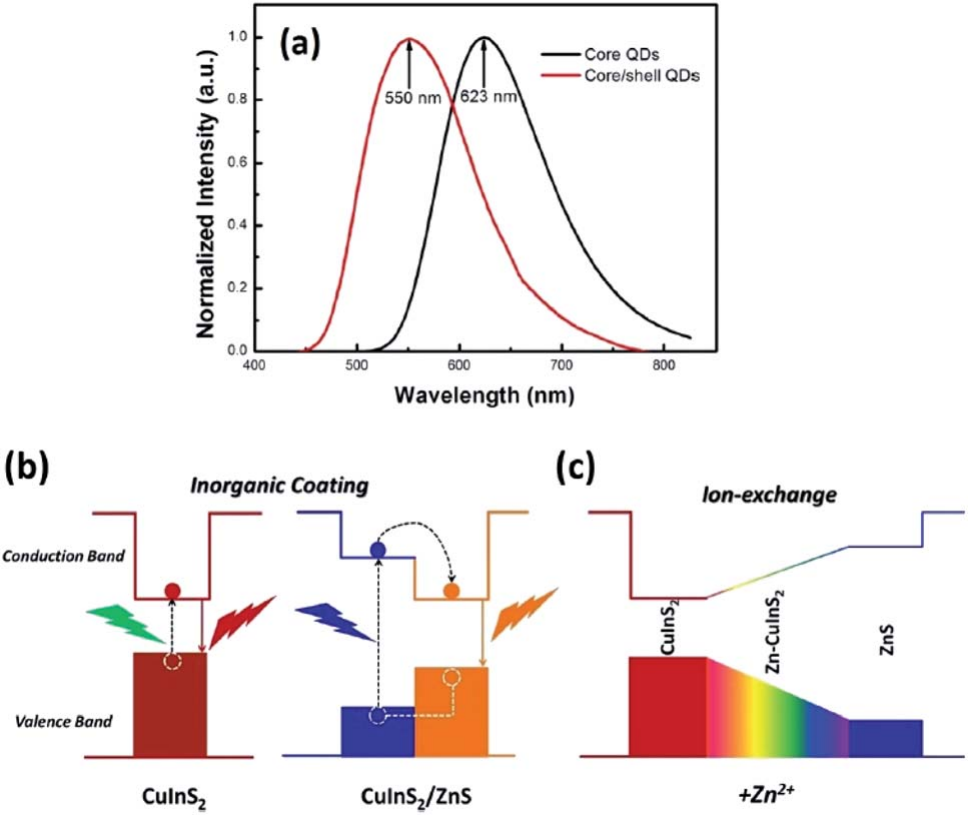
(a) Normalized emission spectra of the CuInS_2_ core QDs and CuInS_2_/ZnS core/shell QDs. (b) Photoluminescence emission mechanism of the CuInS_2_/ZnS core/shell QDs. (c) Schematic of the changes of the energy band gap with the addition of Zn^2+^. The figure was reproduced from ref. 61 with accreditation given to Creative Commons Attribution 4.0 International Public License.

As for inorganic-based materials, large Stokes shift molecules can be derived from a group of metal complexes, organic–inorganic hybrid scaffolds, carbon dots and quantum dots (QDs). In 2015, Li *et al.* recently developed a one-pot method of synthesizing colloidal CuInS_2_/ZnS core/shell quantum dots using the air-stable non-coordinating solvent paraffin liquid to reduce the reaction rate, obtaining spherical shape and monodispersed QDs.^61^ When tested in chloroform, the fluorescence peak was recorded at 550 nm and the full width at half maximum (FWHM) was detected at 125 nm. CuInS_2_/ZnS also has a noteworthy photoluminescence quantum yield that reached up to 81%. In addition, it displayed a large Stokes shift that reached more than 150 nm, thereby making it suitable as a luminescent solar concentrator (LSC). The CuInS_2_ core and Zn–CuInS_2_ nanocrystals dominated the emission wavelength, while the ZnS shell with a wider band gap dominated the absorption wavelength. The typical binary QDs' inorganic coating mechanism (Fig. 8) explains the nature of wider band gap materials, like ZnS, that act as a shell by epitaxial growth on cores with a lower valence band and higher conduction band than that of the core materials to acquire the core–shell structures. On the basis of this mechanism, the inorganic shell coating has a low self-absorption (large Stokes shift) and high quantum yield. Nowadays, the fabrication of eco-friendly carbon quantum dots is attainable by scientists. It was proven by Zhao and co-workers from their synthesis using the space-confined vacuum heating approach with citric acid or urea as precursors and water as the solvent.^62^ In Zhao's study, the large Stokes shift of carbon dots was required for large-scale LSC application, and aimed for the removal of light losses due to reabsorption during light collection. Hence, they designed colloidal carbon dots with a quantum yield of ∼65% and Stokes shift value of 0.53 eV. The proceeded synthetic method was reported to achieve 50% larger Stokes shift than in conventional solvothermal method (Stokes shift of 0.36 eV). The large Stokes shift can be attributed to a recombination of the electron–hole pair with a single type of dominant energy state that differed from the band-gap emission. Also associated with solar cell technology, Khan *et al.* synthesized N-doped graphene quantum dots with an ultra-high photoluminescence quantum yield (99%) and large Stokes shift (98 nm) as downconverters in copper indium gallium selenide (CIGS) solar cells.^63^ Inorganic quantum dots are nano-sized semiconducting crystals, which provide robust tunability of the absorption and emission bands. Through the thermal treatment of graphene oxide and polyethylenimine composite, the doping concentration of the nitrogen and oxygen moieties can be controlled to achieve a desirable photoluminescence quantum yield and Stokes shift value. The conversion efficiency of Khan's solar cell increased up to 15.31% *via* downconversion and light -trapping effect.

The endeavor to explore large Stokes shift inorganic molecules was also realized by Shishino and his group, which pivoted to luminescence in gold nanoparticles (AuNPs).^64^ Even with diameters smaller than 2 nm, this material is luminescent, making them an attractive alternative to quantum dots. The large Stokes shift of AuNPs was ascribed to the luminescence from a low-lying triplet state populated by intersystem crossing (ISC) from the lowest singlet state. Produced by molten matrix sputtering (MMS) method, their AuNPs depicted a large Stokes shift value due to the absorption peak at wavelengths shorter than that of previously reported AuNPs. Since MMS involves a temperature-controlled dispersion medium during the sputtering deposition, the large Stokes shift may correspond to the stabilization of the d band and/or destabilization of the sp-conduction band of the AuNPs. Wen and co-workers also worked on fluorescent Au nanoclusters (AuNCs) with a mega Stokes shift of up to 237 nm that can detect berberine hydrochloride (BHC).^65^ The AuNCs were prepared from citrate-stabilized stannous chloride and hydrogen tetrachloroaurate(iii) as raw materials *via* hydrothermal method. The material was capable of fluorescing at long wavelength emission (566 nm) and quenched gradually upon increasing detection of BHC, which made it remarkably useful as a sensitive and selective spectrofluorometric probe.

Metal complexes are capable of undergoing ultrafast excited state structural deformation. In this context, Zhou and co-workers designed an organic seesaw-shaped tin bromide with deep-red emission ((C_9_NH_20_)_2_SnBr_4_).^66^ This organic metal halide hybrid possessed a zero-dimensional (0D) structure, allowing bulk crystals to exhibit intrinsic properties of individual SnBr_4_^2−^ species in solid state. Decreased dimensionality leads to the emergence of unique properties like exciton self-trapping or excited state structural deformation. Upon photoexcitation, these bulk crystals produced a highly efficient broadband deep-red emission peaked at 695 nm, which has a conspicuous Stokes shift value of 332 nm. The bulk crystals also showed an appreciably high quantum efficiency (∼46%) and long single exponential lifetime (6.51 μs), which is consistent with the characteristics of a phosphorescent molecule. The seesaw structure enabled a pronounced excited state structural deformation as confirmed by DFT calculations, which was responsible for a large Stoke shift. Platinum complex motifs with the mega Stokes shift have been explored by Liu *et al.* by connecting a quinolinium moiety (A) and a triphenylamine derivative moiety (D) with a carbon–carbon double bond.^67^ The tripodal structure with triphenylamine at the centre linked its remaining two arms with pyridine coordinated to Pt moieties. The overall Pt complex showed two absorption bands at approximately 400 nm and 490 nm in acetonitrile solution. The low energy band was tentatively assigned to an intraligand charge transfer (ILCT) transition from triphenylamine to the quinolinium moieties. Emissions were observed at around 530 nm and 700 nm, which originated from a triplet metal-perturbed intraligand (^3^IL) excited state and charge transfer excited state character, respectively. Based on TDDFT study, it was confirmed that the spin density was predominantly localized on the organic moieties of the Pt complex, which further supported the ^3^ILCT character for the low energy emission band (∼700 nm). The enhanced coplanarity of the phenyl ring with the quinolinium moiety in the T_1_ geometry and increased dipole moment from the S_0_ to T_1_ states are believed to contribute to the mega Stokes shift property of the complex. The complex was further tested by being stained on HeLa cells prior to analysis with super-resolution microscopy based on structured illumination microscopy (SIM). The results showed an improvement in the signal-to-noise ratio and good photobleaching resistance.

**Fig. 9:**
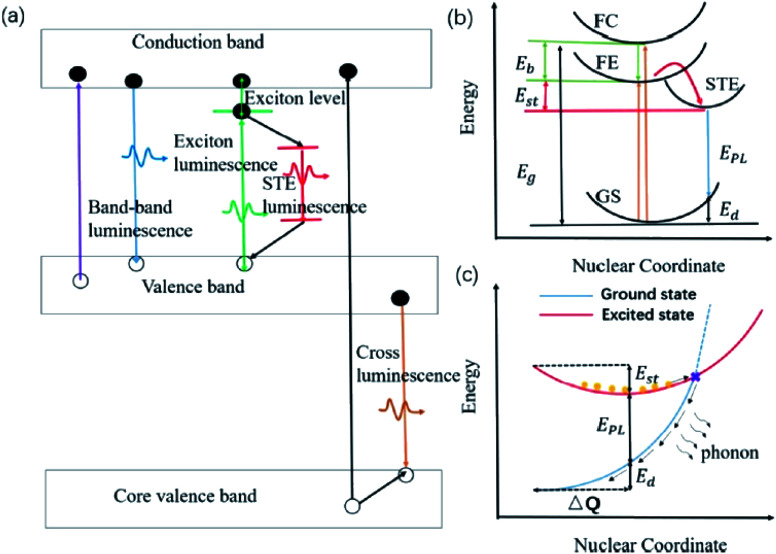
(a) Schematic of various intrinsic photoluminescence phenomena, including band-to-band luminescence, exciton luminescence, STE luminescence, and cross luminescence. (b) Schematic of the energy level structure of STE (GS, ground state; FE, free exciton state; FC, free carrier state; STE, self-trapped exciton state; *E*_g_, band gap energy; *E*_b_, exciton binding energy; *E*_st_, self-trapping energy; *E*_d_, lattice deformation energy; *E*_PL_, emission energy). (c) Schematic of the non-radiative recombination process for STE. Orange circles represent the excited electrons. The figure was reproduced from ref. 69 with permission. Copyright 2019 American Chemical Society.

Zhang and co-workers demonstrated inorganic lead halide perovskite nanocrystals (NCs), which are built on the trigonal Cs_4_PbX_6_ (X= Cl, Br, I).^68^ Cs_4_PbCl_6_ NCs with sizes of 2.2–11.8 nm were synthesized by using the solvothermal method. This type of crystal structure is said to have a wider band gap and distinct optoelectronic properties compared to its cubic counterpart, CsPbX_3_. It has shown a considerably large Stokes shift of 75 nm for the inorganic NCs structure, which can be associated with the lattice relaxation. A more detailed discussion of this type of crystal revealed that the Cs_4_PbX_6_ crystal possesses the Frenkel-type excitons that are trapped by the PbX_6_^4−^ octahedrons. Due to its indirect band gap, this crystal structure has a relatively strong luminescence coming from the self-trapped excitons (STE) at low temperature. Cs_4_PbCl_6_ emitted a single smooth near-UV band that was gradually red-shifted, and peaked at 360 nm as the excitation wavelength increased. The quantum confinement effect accounts for the shifting of the emission wavelength, giving rise to a significant Stokes shift for such small sample. The STE has thus become a useful indicator to assess for large Stokes shift characteristics in all-inorganic halide perovskites. Li *et al.* reviewed several perovskites with STE emission, which were promising for solid-state lighting.^69^ Before delving into the examples of halide perovskites, they outlined the phenomenon of intrinsic luminescence as in Fig. 9. STE has an emission energy that is much smaller than the band gap of the exciton binding energy, and thus generally features a broad spectrum. Materials like Cs_3_Cu_2_I and Cs_2_Na_*x*_Ag_1−*x*_InCl_6_ are said to be good STE emitters due to their capability to suppress the self-absorption, leading to a large Stokes shift. They are capable of generating a large electron-phonon coupling and soft lattice. A mixture of Cs_3_Cu_2_I with a yellow phosphor can induce a white phosphor, while the optimized Cs_2_Na_*x*_Ag_1−*x*_InCl_6_ has demonstrated a warm white light-emitting diode. Another important characteristic of the optimized Cs_2_Na_*x*_Ag_1−*x*_InCl_6_ NCs is that their maximum absorption wavelengths are bound to be in the range of the UV region, thus providing the full potential of the excitons to become more transient within the electronic confinements. For instance, Cs_2_AgInCl_6_ has been shown to reveal an absorption peak at exactly 365 nm upon Na alloying to depress the non-radiative defects, providing a rewarding potential for a high-quality white emission.

**Fig. 10:**
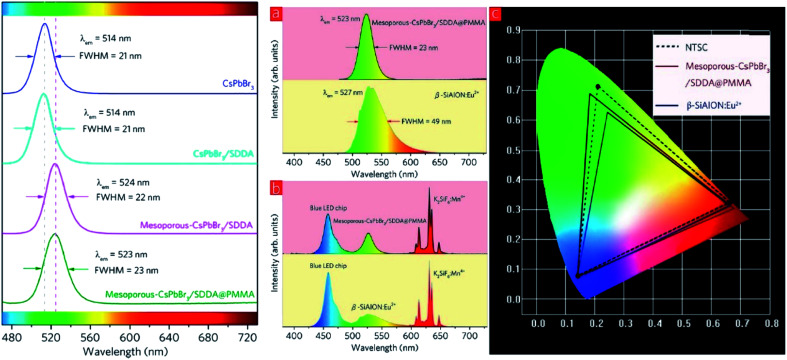
(Left) PL spectra of CsPbBr_3_, CsPbBr_3_/SDDA, mesoporous-CsPbBr_3_/SDDA, and mesoporous–CsPbBr_3_/SDDA@PMMA. (Right) (a) PL spectra of β-SiAlON : Eu^2+^ phosphor and mesoporous-CsPbBr_3_/SDDA@PMMA powder. (b) EL spectra of two white LEDs employing a β-SiAlON : Eu^2+^ phosphor and mesoporous-CsPbBr_3_/SDDA@PMMA powder. (c) Color gamut of two white LEDs employing a β-SiAlON : Eu^2+^ phosphor and mesoporous-CsPbBr_3_/SDDA@PMMA powder, and NTSC. The figure was reproduced from ref. 70 with permission. Copyright 2016 American Chemical Society.

White light-emitting diodes comprise a wide color gamut, and are determined by the color coordinates of RGB. Research efforts on the luminescent properties based on green and red emitters have been continuously pursued to gain a narrower color band that would contribute impressively to the white light. Among the inorganic green emitters designed, perovskite CsPbBr_3_ quantum dots have been particularly interesting and promising due to their high photoluminescence quantum yield. Nevertheless, there are several recognized issues, including the still insufficient efficiencies due to the surface trap states, low thermal stability, and low tolerance to humidity. For these reasons, Zhang and co-workers proposed an inorganic–organic hybrid pair (didodecyl dimethylammonium sulfide, S^2−^–DDA^+^) to passivate the surface defects of the current quantum dots.^70^ Moreover, they suggested a mixture of the CsPbBr_3_ quantum dots with mesoporous silica particles to overcome the thermal instability, and the subsequent blending of the mesoporous-CsPbBr_3_/SDDA with polymethylmethacrylate (PMMA) to diminish the sensitivity of this green emitter towards moisture. White LEDs were fabricated as a proof-of-concept, and it was found that the color gamut achieved up to 102%. Fig. 10 shows the red-shifted emission from the new designation of the inorganic green emitter, which substantially contributed to white light application upon the mixture built-in with the blue LED chip and red emitter of K_2_SiF_6_ : Mn^4+^. It was noteworthy that even a slight enlargement of the Stokes shift value could significantly render an approaching ideal white light emission and color gamut.

## 3. Molecular design for large (mega) Stokes shift material

The facile mixing of multiple emitters can cause unfavorable interactions between molecules, making it difficult for chemists to design the appropriate molecular structure, be it for organic or inorganic materials. Currently, much effort has been devoted to proposing materials that exploit the concept of ESIPT, ICT, excited state structure deformation, hydrogen bonding mediated J- or H- aggregation, aggregation induced emission (AIE), interchange between monomer and excimer fluorescence, host–guest interaction, metal–ligand charge transfer (MLCT), among others.

With this in mind, the photophysical processes will be highlighted and discussed by taking various examples from previously reported materials. The discussion will also cover both individual materials, which can single-handedly produce white light emission, and combinatorial materials being integrated together as a part of the white light system.

### 3.1 Excited state intramolecular proton transfer (ESIPT)

The ESIPT reaction usually incorporates the transfer of a proton donor (*e.g.*, hydroxy or amino proton) to proton acceptor moieties, like carbonyl oxygen or pyridyl nitrogen, in the proximity of a molecule.^71^ Some remarkable properties of ESIPT are the large Stokes shift,^72,73^ dual emission,^74^ ultrafast process,^12^ and the spectral sensitivity to nanoenvironments.^73^ The most widely investigated ESIPT fluorophores revolve around the conventional proton donor or acceptor molecules, such as in 2-(2′-hydroxyphenyl)benzimidazole (HBI), benzoxazole (HBO) and benzothiazole (HBT) derivatives.^75–77^

**Fig. 11:**
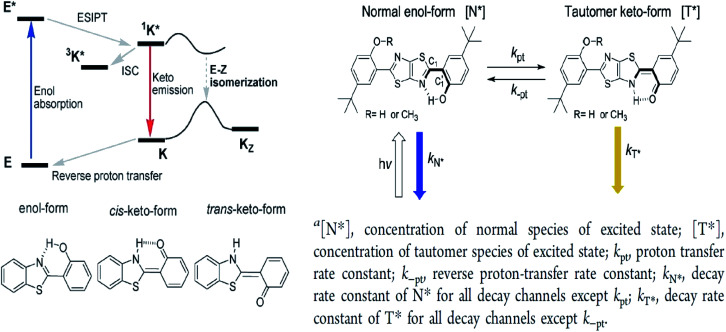
(Left) Principal photophysics of ESIPT. Illustrated by 2-(2′-hydroxyphenyl)benzothiazole. The figure was adapted from ref. 12 with copyright permission from the PCCP Owner Societies. (Right) Reversible ESIPT reaction pattern for two thiazolo[5,4-*d*]thiazole derivatives. The figures were adapted from ref. 78 with permission. Copyright 2016 American Chemical Society.

In this perspective, the basic photophysics of ESIPT chromophores have been discussed by Zhao *et al.*, which focuses on the state-of-the-art development of new luminescent materials.^12^ It should be mentioned that chemically modifying the conventional fluorophore to attain an increased Stokes shift can be rigorous. Fig. 11 illustrates the basic photophysical process of ESIPT in a 2-(2′-hydroxyphenyl)benzothiazole (HBT) compound. An intramolecular hydrogen bond between nitrogen and hydrogen atoms is formed at the ground state, giving rise to the *cis*-enol form. When irradiating photons, the excited state is populated with the enol form, followed by an ultrafast ESIPT process that subsequently produces the *cis*-keto form at the singlet excited state. The keto tautomer usually accounts for the fluorescence observed in the ESIPT chromophore. There is also a deactivation channel (ISC) of the *cis*-keto form, which leads to a low-lying triplet excited state. Furthermore, a *trans*-keto structure may also form *via* the isomerization non-radiative decay. The decay of these two events, *i.e.*, triplet excited state keto tautomer and *trans*-keto to *cis*-keto transformation occurs at the μs timescale, which can be considered as slow processes. Both processes can be distinguishable by time-resolved spectroscopy, whereby the decay of the triplet excited state is sensitive to O_2_, whereas the transformation of the *trans*-keto to *cis*-keto form is pretty much unaffected by O_2_.

Also revolving around the ESIPT system, Zhang *et al.* reported thiazolo[5,4-*d*]thiazole (TzTz) derivatives that involved a proton (or hydrogen atom) transfer from a pre-existing hydrogen bond, which has led to a proton-transfer tautomer in the excited state.^78^ In theory, the reaction dynamics of the unimolecular ESIPT system can be harnessed *via* chemical modification or external factors in a way that the fluorescence is coming from both reactant and product. This provides the possibility to fine-tune the overall luminescence properties of the single compound to generate white light. The strategy to establish the ESIPT system in their study was to integrate two phenol-substituted groups onto the TzTz proton acceptor core, as in Fig. 11. All TzTz molecular structures were confirmed to be planar based on the X-ray diffraction analyses. There was no substantial π–π stacking because of two very bulky *tert*-butyl groups. This bulkiness was inserted to increase the solubility of the molecule, which is a key factor for the later femtosecond fluorescence up-conversion study and substantial reduction of any intermolecular contact (solvent effect). A large Stokes-shifted yellow emission emerged around 560 nm for derivatives of TzTz. This emission peak has been assigned to the proton-transfer tautomer emission originating from ESIPT. Fast equilibrium between the two species, N* and T*, prior to their respective emission contributed to identical population decay time constants between the two. The emission intensity ratios of the TzTz derivatives were sensitive to changes in the solvent polarity. On passing from non-polar (cyclohexane) to polar aprotic (dichloromethane), it was found that the tautomer emission predominated and led to a smaller quantum yield. The mechanism of ESIPT worked in an uneven electron density distribution, arising from enol–keto tautomerization on only one side of the symmetric molecule. This led to an enlargement in the dipole moment in T*, which stabilized further as the solvent polarity increased.

Yadav and co-workers also explored the ESIPT properties of rhodol derivatives that were linked to benzimidazole and benzothiazole units.^79^ Rhodol is generally a hybrid form of fluorescein and rhodamine, which is well-known for its good solubility, photostability and high fluorescence quantum yield. Interestingly, the rhodol derivatives that they designed displayed large Stokes shifts (50–260 nm) in spirocyclic forms, which were assigned to the ESIPT process. The benzimidazole and benzothiazole units were strategically introduced adjacent to the oxygen atom of the rhodol core fragment in expectation that the ESIPT process is prompted between the OH (rhodol) and N atom (benzimidazole/benzothiazole). Spirocyclic-rhodols emitted at longer wavelengths, *ca.* 428–525 nm, than their opened counterparts. An additional privilege of this structural framework is that it possessed strong solvatochromic properties when tested from non-polar to polar solvents. Such phenomenon could be explained by dipole–dipole interaction and solute–solvent interaction, which was proven by Lippert–Mataga and Mac-Rae functions. These advantages provide an avenue for applications in photochromic and laser dyes, and fluorescence recording techniques. Barman *et al.*, on the other hand, designed a drug delivery system (DDS) that exploited the property of ESIPT.^80^ A benzothiazole unit was attached to the 8-position of 7-hydroxycoumarin before conjugating with the anticancer drug, chlorambucil (Cou-Benz-Cbl). The interconnection between the three organic units successfully produced a large Stokes shift up to 151 nm, which can be ascribed to ESIPT between OH (coumarin) and N atom (benzothiazole). In polar protic (ethanol and methanol) and polar aprotic (acetonitrile and tetrahydrofuran) solvents, the compound emitted dual emission at 406 nm and 516 nm, respectively. The lower energy emission can be attributed to keto formation in the excited state upon photoirradiation. Barma also found that upon strengthening of the hydrogen bonding in a binary mixture of methanol : water, the keto emission intensity became more pronounced.

**Fig. 12:**
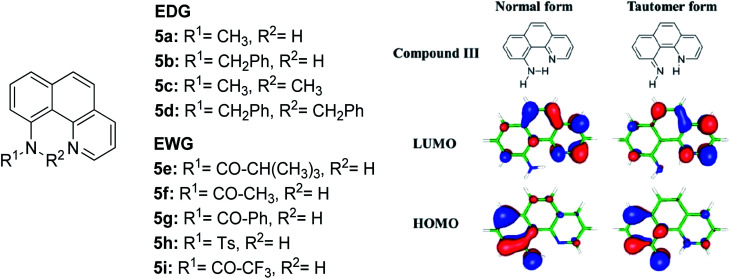
Molecular structures of 10-aminobenzo[*h*]quinoline derivatives (left), and calculated HOMO and LUMO molecular orbitals of the normal and tautomer forms of unsubstituted compound 5 (right). The figure was adapted from ref. 81 with copyright permission from The Royal Society of Chemistry.

The N–H type ESIPT emitter has an advantage over the O–H type because of the three valences of the nitrogen atom. One of them can be used to tailor the molecular structure to control the proton migrating ability and tune the electronic properties. Tseng and co-workers designed a facile, single site amino derivatization in 10-aminobenzo[*h*]quinoline by replacing one of the N–H hydrogen atoms with varying substituents to construct a series of excited state intramolecular N–H transfer molecules (Fig. 12).^81^ When one of the amino protons was substituted by a strong electron-withdrawing group, it would increase the N–H acidity, which promoted the ESIPT process. The general comparison between the presence of these two opposing behaviors within the parent compound is revealed when EWG groups (5e–5i) were shown to emit light in more red-shifted regions (590 nm–740 nm) than EDG groups (5a–5d). The compound that has the stronger intramolecular hydrogen bonding strength will have a larger downfield shift of the N–H proton and better acidity character. Hence, a faster and highly exergonic type of ESIPT was achieved with increased proton donating strength. This can be rationalized by varying the EWG on the amino site, which results in lowering the HOMO energy and increasing the energy gap of the imino-tautomer emission.

**Fig. 13:**
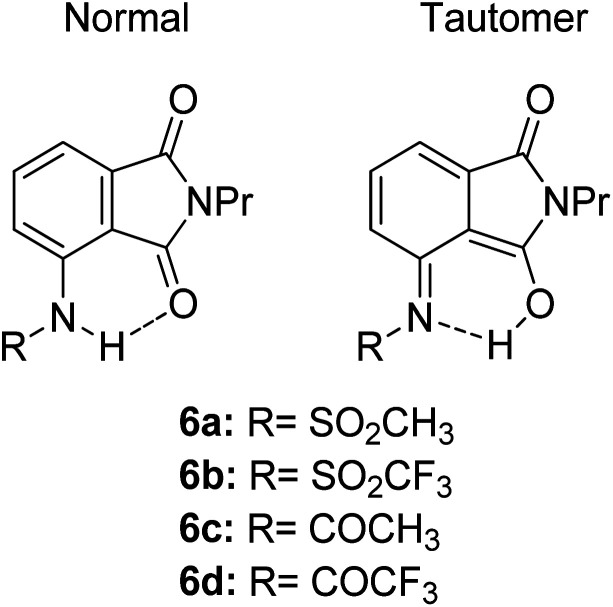
Chemical structures of phthalimide of the normal amide form and its tautomer. The compounds are from ref. 82.

Another ESIPT-based fluorescence was demonstrated by Okamoto and group through the solid-state emission of 3-amidophthalimides.^82^ All derivatives (Fig. 13) showed an absorption band in the range of 323–345 nm. Compound 6a produced red fluorescence at a wavelength of 625 nm with a very large Stokes shift of 13 400 cm^−1^ when tested in toluene. Similarly, for the remaining derivatives, this behavior can be resolved by the ESIPT phenomenon through their respective tautomer formation upon excitation of the molecules. Compound 6d showed dual emission at 391 nm and 565 nm, appearing yellow when tested in the solvent toluene. The fluorescence at 565 nm accounts for the ESIPT process due to the fast equilibrium between the relaxed amide and its tautomer species. The aim in modifying the molecular structure for a large Stokes shift property has thus been rectified by tuning the enol and keto emission bands through the ESIPT process. There are two forms of which researchers can opt to approach for obtaining ESIPT-based fluorescence, *i.e.*, tuning molecules in solution or in solid states. Therefore, the fluorescence intensity of molecules can be further optimized so that an appreciable quantum yield can be achieved for the development of white light emission. This can be carried out by introducing different nanoenvironments to the molecule that are soluble in solvents (using polar aprotic or non-polar solvent), while solid-state fluorescence can also be regarded since the restricted conformation of the molecule promotes an enhanced quantum yield. Since ESIPT is always accompanied by a diminished fluorescence quantum yield, the problem has taken the interest of Zhang *et al.* They addressed the issue by undertaking the role of the H-donor nitrogen in imidazole, which usually has a higher quantum yield by one order of magnitude than that of benzoxazole and benzothiazole. Besides, imidazole or benzimidazole promotes more intermolecular interactions, which is beneficial for molecular aggregations.^83^ 3-(4,5-Diphenyl-1*H*-imidazol-2-yl)naphthalen-2-ol (DPIN) was designed after examining two phenyl tails on imidazole that can restrict intermolecular π–π stacking, thereby inhibiting aggregation-caused fluorescence quenching. When dispersing DPIN in water, stable homo-dispersed aggregated nanoparticles were formed, showing an almost exclusive keto emission with a quantum yield of 0.20. More fascinating quantum yields were observed in DMF and DMF : water with keto emission values of 0.28 and 0.39, respectively. The fluorescence performance of DPIN was stable in the physiological pH range of 6.2–9.2 without interference from common ions, making DPIN suitable as a sensing material. Additional improvement for a high fluorescent quantum yield with ESIPT character was demonstrated by Mutai *et al.* by spin-coating 0.5 wt% of the fluorophores of interest within a polymer matrix (PMMA).^84^ ESIPT molecules in a polymer matrix produced fluorescence with enhanced and more efficient quantum yield (*Φ*_F_ = 0.6), as compared to that in fluidic forms. Herein, we reviewed the criterion to which an ESIPT mechanism can occur within a molecule. The crucial prerequisite is the formation of a tautomer in the excited state that would red shift the emission wavelength further, leading to a large or mega Stokes shift feature.

### 3.2 Intramolecular charge transfer (ICT)

Another way to design a material with a large Stokes shift property involves the ICT mechanism, which is usually established within a molecule with a donor–acceptor (D–A) electronic system. This single emitter system is capable of revealing unique two or multi-color emissions, caused by the change in the twisting angles between the D and A units.^85^ Generally, organic molecules with extended π-conjugation, end-capped by D and A offer a facile synthetic route, molecular arrangement and unique luminescence properties that become notable in the field of material science.^86^ The promising avenue of the ICT mechanism in such system has led to the development of thermally activated delayed fluorescence (TADF) materials, which could maneuver future emitters in the field of OLED. D–π–A molecules represent a sub-class known as the push–pull dye due to the dispersed polarization during ICT. There are many push–pull dyes present in the literature, but only a few classes will be discussed in this review. The key approach to evaluating the ICT mechanism is to skim the D and A moieties in the molecular structure.

**Fig. 14:**
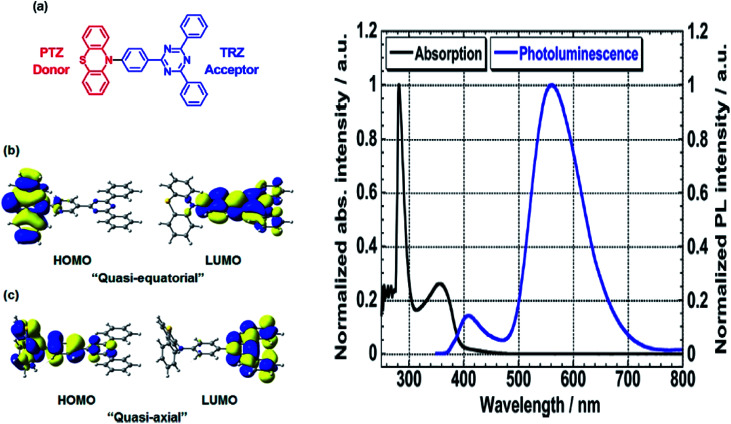
(Left) (a) Molecular structures, and (b and c) HOMO and LUMO of the ground state quasi-equatorial and quasi-axial conformers of PTZ–TRZ calculated at the CAM-B3LYP/cc-pVDZ level, respectively. (Right) Normalized absorption (black line) and PL (blue line) spectra of PTZ-TRZ in toluene solution with a concentration of 1.0 × 10^−5^ M. The PL spectrum was obtained by excitation at 340 nm. The figures were reproduced from ref. 87 with permission. Copyright 2014 American Chemical Society.

The most developed arrangements are linear (D–π–A), quadrupolar (D–π–A–π–D or A–π–D–π–A)^28^ and octupolar/tripodal ((D–π)_3_–A or (A–π)_3_–D) systems. A typical D is represented by substituents like OH, NH_2_, OR, NR_2_, or heterocyclic moieties like thiophene and carbazole. On the other hand, A usually features substituents like NO_2_, CN, CHO, CF_3_, SO_3_R, and electron-deficient heterocycles like imidazole, quinoline and benzothiazole.^86^ Halogens like F, Cl, Br and I can be classified as weak A. In this regard, selected systems of phenothiazine, carbazole, pyridine and rhodamine will be discussed in this section. Tanaka and his co-workers introduced a very efficient up-conversion mechanism of excitons from the spin-triplet (T_1_) to spin-singlet (S_1_) charge transfer excited state^87^ through the phenothiazine–triphenyltriazine (PTZ–TRZ) derivative as shown in Fig. 14. The HOMO and LUMO in such molecular system are said to be effectively separated to allow the transfer of the triplet excitons through a small energy gap. The PTZ–TRZ molecule showed a broad absorption and emission, arising from the twisted ICT state. Two emission signals with peaks around 409 and 562 nm were generated when excited at 340 nm. The large Stokes shift at 562 nm emission was attributed to the ICT emission from the spin-singlet excited state (S_1_) to spin-singlet ground state (S_0_). Pivoting to the phenothiazine system as well, Zhan *et al.* synthesized a phenothiazine-based benzothiazole (PVBT) system that was able to emit light from 483 nm (in *n*-hexane) to 580 nm (in DMF).^88^ With increasing solvent polarity, the Stokes shifted too, increasing from 9599 cm^−1^ to 11 913 cm^−1^. The broadening of the lower energy emission maximum indicated an ICT characteristic. PVBT further displayed superiority in its photoluminescence property with a maximum quantum yield of 0.76 in cyclohexane. A lower *Φ*_F_ was found in DMF (0.02), and was associated with a strong ICT process and dipole–dipole interaction between the molecules. Verification of the ICT process in PVBT was done using the TDDFT method, where they found that the HOMO was mainly distributed over the electron donor (phenothiazine unit), while the LUMO was concentrated on the electron acceptor benzothiazole unit.

Among the acceptor units, quinoline derivatives are often investigated due to their high thermal and chemical stability, high optical responses, and efficient electron transport properties. A recent study by Slodek *et al.* exploited the role of quinoline in comparing the fluorene's and carbazole's electron donating character.^89^ The construction of 2,4-difluorenylquinoline derivatives has successfully produced bright emission in the blue spectral region (∼400 nm) and high fluorescence efficiency in solution (63–97%). The carbazole-substituted compound showed a strong solvent effect on the emission wavelength with increasing Stokes shift value of up to 6366 cm^−1^ in more polar solvent. This corroborated with the phenomenon of ICT occurring between the D–A units. The study of the carbazole units was also emphasized by Yadav *et al.* by comparing the strength of the donor units between triphenylamine and carbazole.^90^ The absorption and emission bands of the triphenylamine-coumarin derivatives were found to be slightly red-shifted compared to that of the carbazole-coumarin derivatives. Generally, large Stokes shifts were obtained from both types of derivatives (81–135 nm). The strength of the electronic ICT in D–π–A dyes can be measured to predict the solvatochromism using the generalized Mulliken–Hush (GMH) method. Positive solvatochromism is achieved when a highly polarized excited state is created. There are two ways to facilitate polarization of a molecule, *i.e.*, to engineer the structural framework and substituents present in a molecule, and controlling its nanoenvironments (solvent, pH, temperature, presence of dissolved oxygen, concentration).

Recently, Zhu and co-workers developed an ICT-based fluorescent probe with a large Stokes shift for determining the highly active alkali, hydrazine.^91^ 4-(2-(3-(Dicyanomethylene)-5,5-dimethylcyclohex-1-enyl)phenyl)4-bromobutanoate (DDPB) was synthesized as the sensing material that exhibited a large Stokes shift (186 nm). The ICT pathway facilitates the connection between the external reactive groups with the donor or acceptor moiety of the sensing material to obtain a measurement proportional to the dual emission wavelength. The substitutional cleavage cyclization reaction between the DDPB and hydrazine in their study promotes a trailblazing idea among researchers to design a single emitter that allows the ICT process to occur either intermolecularly or intramolecularly (accounting for the targeted white light emission) for a more red-shifted emission.

**Fig. 15:**
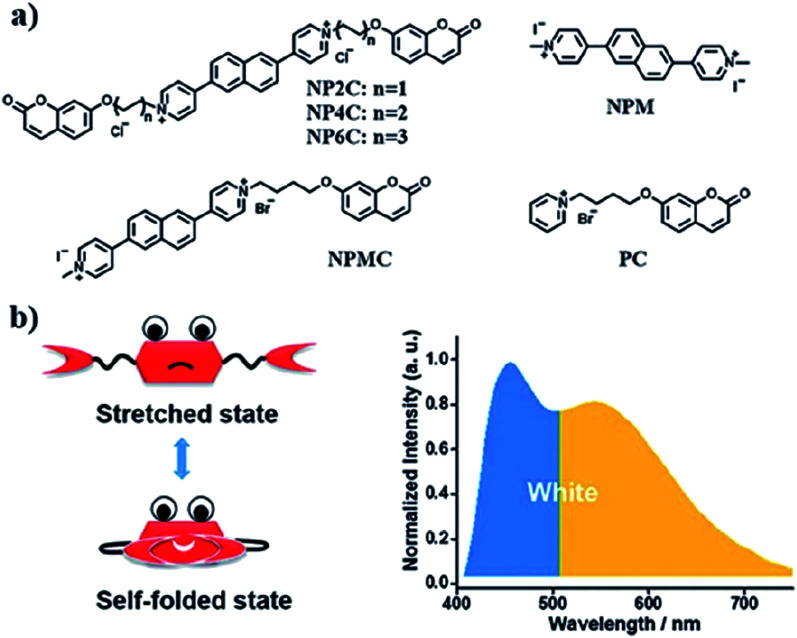
(a) Molecular structures of white-light emitting organic molecules (NP4C and NP6C) and the reference compounds (NP2C, NPM, NPMC and PC); (b) the schematic cartoon illustration of single-molecule white-light emitters. The figure was reproduced from ref. 92 with copyright permission from The Royal Society of Chemistry and accreditation given to CC BY-NC 3.0.

A new bi-functional organic molecule with a symmetric D–A–D type structure was designed by Li *et al.* using pyridinium–naphthalene (PN) core connected to two coumarin moieties *via* flexible alkyl chains.^92^ NP4C (Fig. 15), which exhibits the natural self-folded conformation, could enhance the ICT efficiency, leading to a broad emission spectrum. NP4C and NP6C were reported to be capable of emitting white light with a CIE coordinate of (0.30, 0.33), arising from their dual-emissive properties when tested in water. This can be rationalized by the different existing chromophores at distinct regions in both compounds, which can construct a significant difference in the frontier molecular orbitals. For NP4C, the HOMO was primarily located in the coumarin groups, while the LUMO was distributed on the PN core in favor of the ICT process. The presence of the varying flexible alkyl chain lengths operates the rotation and motion of the coumarin moieties at both ends, which might lead to stacking with the PN core due to hydrophobic effects, π–π stacking and donor–acceptor interactions, which further favor the CT emission. The interesting output from this research is the fact that intramolecular motions and further accelerated transformation between the self-folded and stretched states of NP4C can be achieved by gradually increasing the temperature of the aqueous solution. A similar palette was attempted by Gandioso and his group, where a compound emitting in the NIR and a large Stokes shift (99 nm) were accomplished.^93^ A pyridinium moiety was attached at the 2-position of a coumarin core, which acted as the pull unit, inducing a more polar environment to the coumarin scaffold in the ground state. This occurrence blue-shifted the absorption band of the overall molecule, while the emission band stayed the same due to its insensitivity to solvent changes. Based on the NMR spectroscopy, the existence of *E* and *Z* rotamers in solution was confirmed, and they were responsible for further intensifying the push–pull effect.

**Fig. 16:**
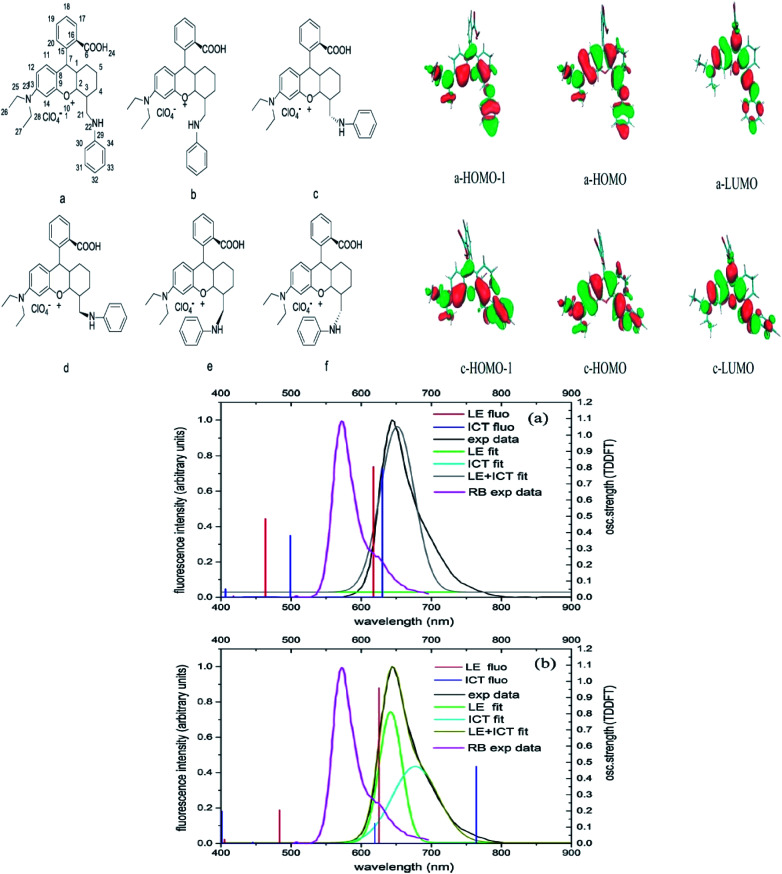
Six stable isomers for anilino-substituted Rhodamine analogues and visualization of HOMO-1, HOMO and LUMO+1 of the complex 7 (top). Experimental and theoretical fluorescence spectra, the deconvoluted local excited state (LE) and ICT bands for (a) 7a; (b) 7c (bottom). The figures were reproduced from ref. 95 with copyright permission from Elsevier.

If we are to explore the reasoning of dual fluorescence that comes from small molecules, Lippert's viewpoint would be conspicuous to consider. Donor and acceptor units linked together will have a limited degree of freedom. Therefore, the most preferred vibrational mode for a relaxation involving a change in the electronic structure of the excited state is the large amplitude torsional motion or twist.^94^ The features of the phenomenon are further described to be strongly dependent on the surrounding conditions, like the polarity of the solvent and the thermodynamic effect. The twisted intramolecular charge transfer (TICT) has been discussed by Zhang *et al.* by demonstrating the photophysical process of aniline-substituted rhodamine analogues using DFT/TDDFT approaches.^95^Fig. 16 presents the possible stable isomers of compound 7, 9-(2′-carboxylphenyl)-6-(*N*,*N*-diethylamino)-4-(*n*-phenyl-methanamine)-1,2,3-trihydroxanthylium. The isomers 7c–f are said to be twisted, unlike 7a and 7b, which are completely planar. The geometry of the relaxed S_2_ excited state of 7c shows that the anilino moiety is planar to the xanthene plane, suggesting that TICT may occur in this isomer. Compound 7 possesses rather red-shifted absorption bands and simultaneously, a red-shifted emission wavelength (∼600 nm). TICT is responsible for the large Stokes shift of compound 7 due to the presence of a strong electron-withdrawing anilino unit within the rhodamine precursor.

Based on the discussed examples, we have shown that the addition of specific substituted groups and modulating steric restrictions from eith–er bulkiness or π–π interactions can shift the absorption and emission simultaneously. Tuning the ICT process in a push–pull system becomes eminent in regulating the charge distribution over a molecule. Effects of the NO_2_ group have been covered by Panja and his research group, where they varied the number and position of NO_2_ groups that exist in a phenolic core. It was discovered that one strong and highly feasible ICT process happened in the *para* position of nitrophenolate that produced absorption at 420 nm (in acetonitrile). The ICT character increased when the number of NO_2_ groups increased in the molecule. This minute alteration changed the transition orbitals from HOMO to LUMO and HOMO to LUMO+1.^96^ Transfer of electronic charge fractions between the molecular entities causes a huge loss of excitation energy, resulting in large Stokes shift phenomenon. Certainly, ICT is one of the encouraging approaches to consider when developing a white light emitter with large Stokes shift properties. In order to generate a true white light, detailed photophysical mechanisms of the molecule need to be understood, simulated and designed prior to synthesis execution. The details of photophysical properties such as maximum absorption and emission wavelength, fluorescence quantum yield and fluorescence lifetime of some ICT-active molecules introduced in this review are summarized in Table 1.

**Table 1:** Photophysical properties of some ICT-active molecules

Compound	*λ*_abs_ (nm)	*λ*_em_ (nm)	*Φ*_F_	*τ* (ns)	References
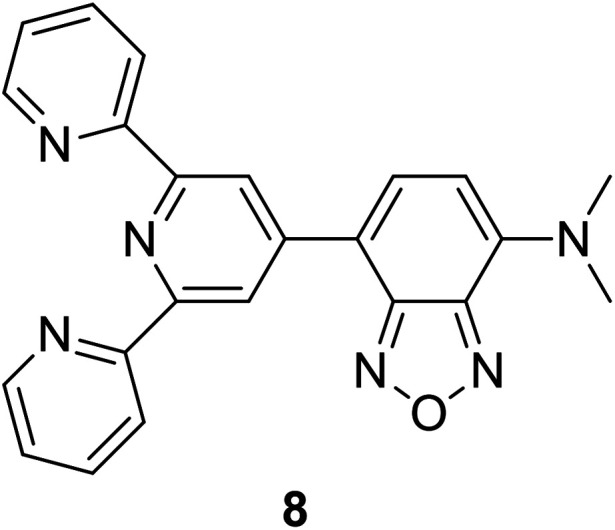	279, 336, 470 (MeCN : H_2_O)	624	0.036	—	33
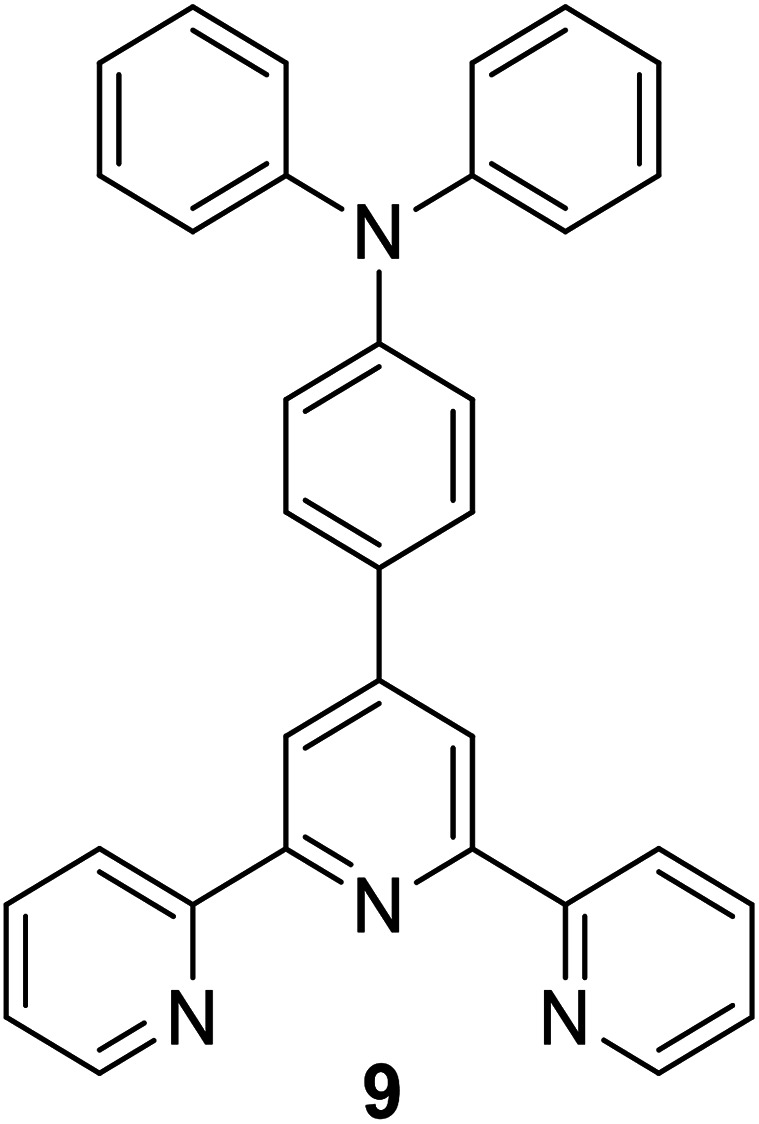	354 (*n*-hexane)	393 (*n*-hexane)	—	—	34
	360 (CH_2_Cl_2_)	476 (CH_2_Cl_2_)			
	357 (MeCN)	489 (MeCN)			
	362 (EtOH)	505 (EtOH)			
	359 (MeOH)	524 (MeOH)			
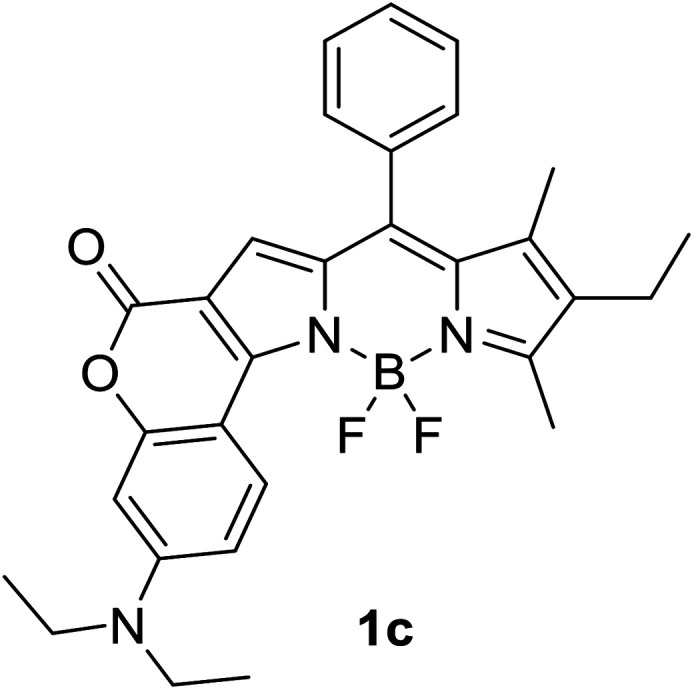	620 (CyH)	643 (CyH)	0.86 (CyH)	—	40
	632 (PhMe)	632 (PhMe)	0.62 (PhMe)		
	602 (CH_2_Cl_2_)	697 (CH_2_Cl_2_)	0.41 (CH_2_Cl_2_)		
	590 (MeOH)	720 (MeOH)	0.06 (MeOH)		
	606 (DMF)	748 (DMF)	0.04 (DMF)		
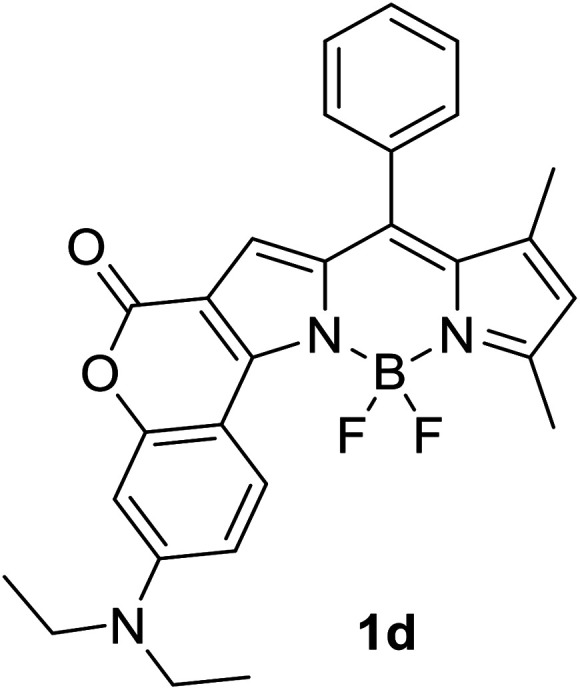	620 (CyH)	642 (CyH)	–(CyH)	—	40
	606 (CH_2_Cl_2_)	706 (CH_2_Cl_2_)	0.31 (CH_2_Cl_2_)		
	594 (MeOH)	724 (MeOH)	–(MeOH)		
	608 (DMF)	752 (DMF)	0.03 (DMF)		
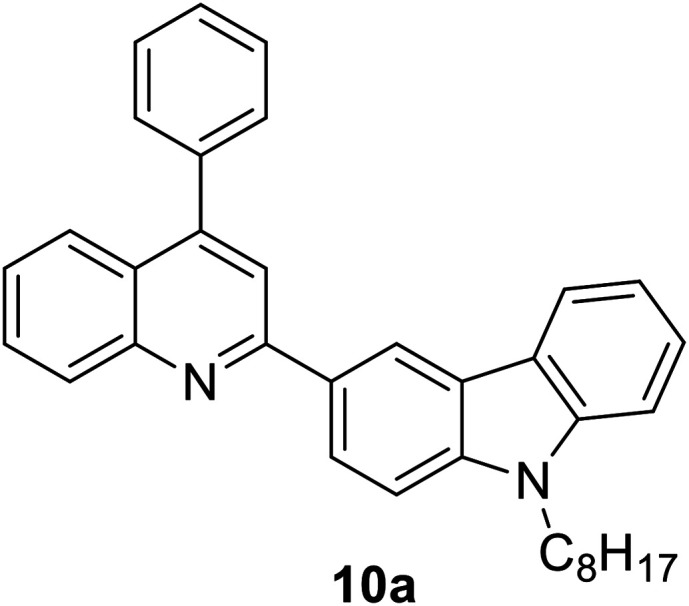	343 (CH_2_Cl_2_)	431 (CH_2_Cl_2_)	0.63 (CH_2_Cl_2_)	2.57 (CH_2_Cl_2_)	31
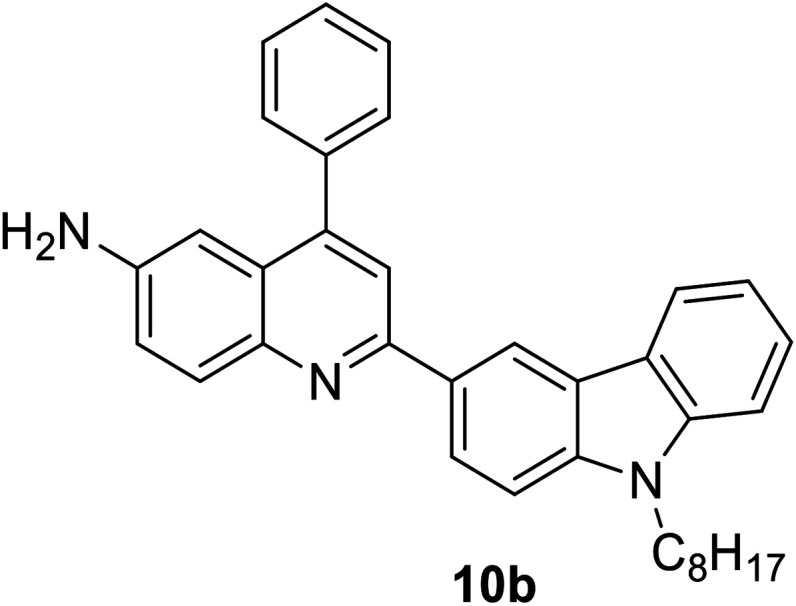	377 (CH_2_Cl_2_)	440 (CH_2_Cl_2_)	0.70 (CH_2_Cl_2_)	5.72 (CH_2_Cl_2_)	31
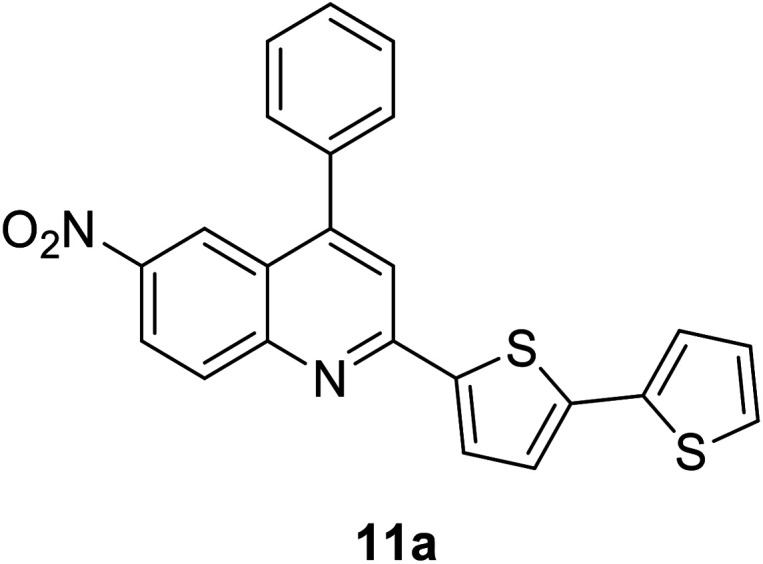	412 (CH_2_Cl_2_)	595 (CH_2_Cl_2_)	0.03 (CH_2_Cl_2_)	1.42 (CH_2_Cl_2_)	31 and 97
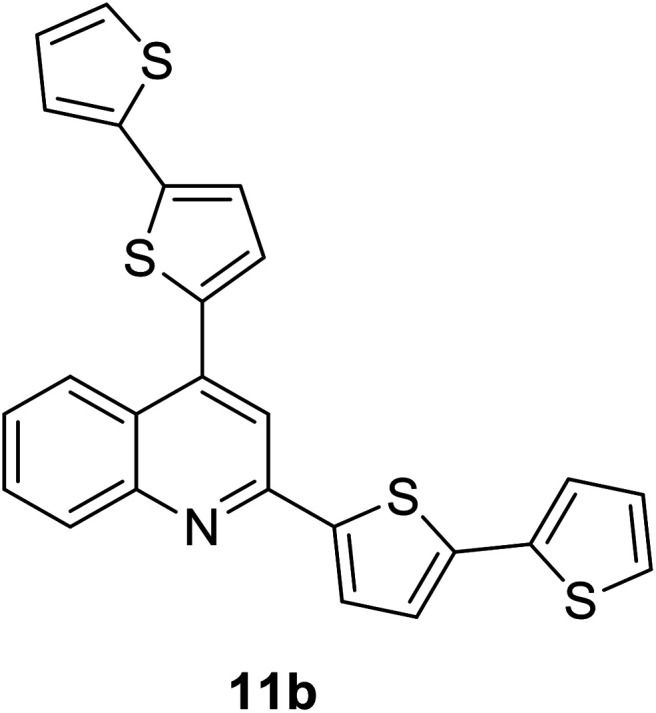	380 (CH_2_Cl_2_)	479 (CH_2_Cl_2_)	0.53 (CH_2_Cl_2_)	1.13 (CH_2_Cl_2_)	31 and 97

### 3.3 Excited state geometry relaxation and structure deformation

**Fig. 17:**
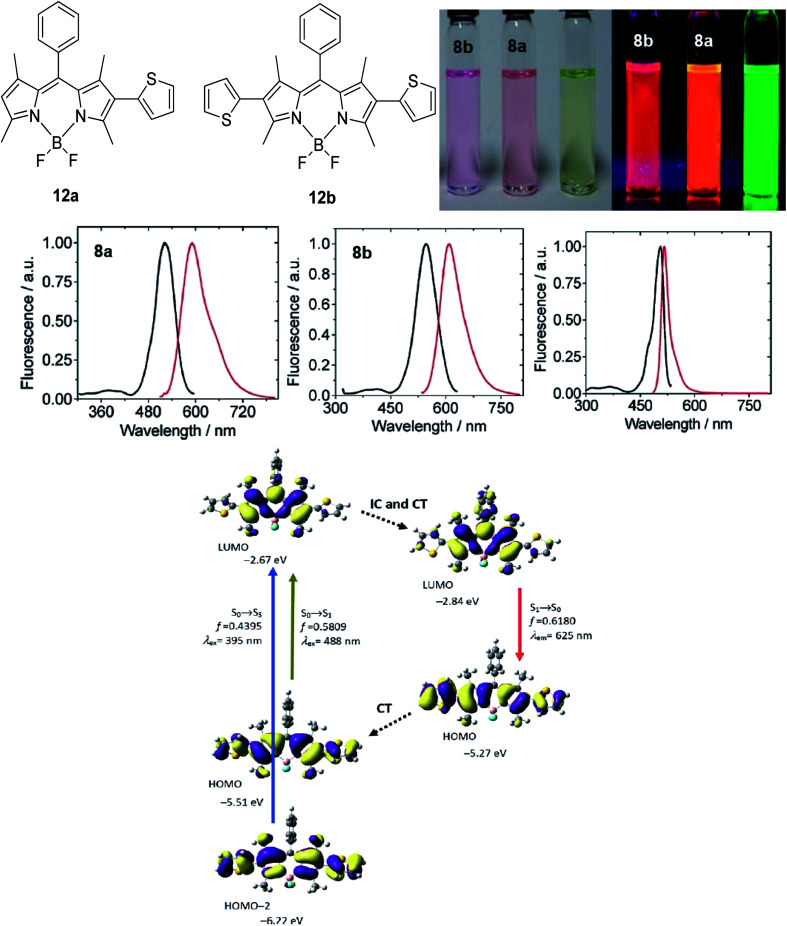
(Top) Molecular structure of 2-thienyl (12a) and 2,6-bisthienyl (12b) BODIPY derivatives and their emission color, taken under ambient light and UV light (handheld UV lamp, 365 nm). *c* = 7.5 × 10^−6^ M (toluene, 25 °C). (Middle) Fluorescence spectra of 12a, 12b and unsubstituted BODIPY. (Bottom) Rationalization of the large Stokes shift of 12b: the geometry relaxation upon photoexcitation, and the frontier molecular orbitals (MOs) involved in the vertical excitation (*i.e.*, UV-vis absorption, the left two columns) and emission (right column) of 12b. The figures were adapted from ref. 98 with permission. Copyright 2012 American Chemical Society.

The designation of molecules with a large Stokes shift property involves compromise on the drawback of self-absorption. Self-absorption is due to the proportions of the fluorescence wavelength being re-absorbed by the same or other molecules for subsequent fluorescence at a longer wavelength region. To address this issue, the *de novo* approach is to increase the geometry relaxation of the molecular framework upon photoexcitation. This method has been described by Chen *et al.* in their study by introducing thienyl substituents at the 2- and 6-position of a BODIPY core (12a and 12b).^98^ The output from DFT calculations proved that there was a change in the dihedral angle of the compound (between the thienyl unit and the BODIPY core) from 59° to 39° when comparing the geometry in the S_0_ and S_1_ states. The delineation for this is an increased difference between the emission energy as compared to the excitation energy that subsequently led to an enlarged Stokes shift. The excitation and emission maxima for 12a were at 509 nm and 601 nm, respectively, giving a Stokes shift value of 92 nm, while 12b had a Stokes shift value of up to 106 nm, giving a deep red fluorescence (Fig. 17). Other than assessing the dihedral angle changes in the excited state, recognizing any sterically hindered or restrained part of a molecule could shine light in the large Stokes shift material designation. Doroshenko *et al.* studied the *ortho* analogues of alternating the phenyl-oxazole-phenyl-oxazole-phenyl (POPOP) ring, which induced high steric hindrance and nonplanarity of the molecule.^99^ Such conformation and arrangement flattened the excited singlet state of the molecule, which further lowered the fluorescent energy and in turn, gave an enlargement of the Stokes shift. POPOP generally has an intramolecular interaction nature due to the bulkiness of the moieties, and thus experiences a minor influence of the environments. When the oxazole ring was replaced by oxadiazole instead, a nearly doubled flattening of the activation energy was observed, meaning a larger Stokes shift was achieved. This happened because the light was mainly absorbed by the phenyl-oxadiazolyl fragment, causing the molecule to rotate out of the main plane due to the sterically hindered oxadiazolyl in the electronic absorption spectra. The fluorescence quantum yield observed for POPOP derivatives were reasonably high, from 0.5 to 0.6, depending on the solvent used.

**Fig. 18:**
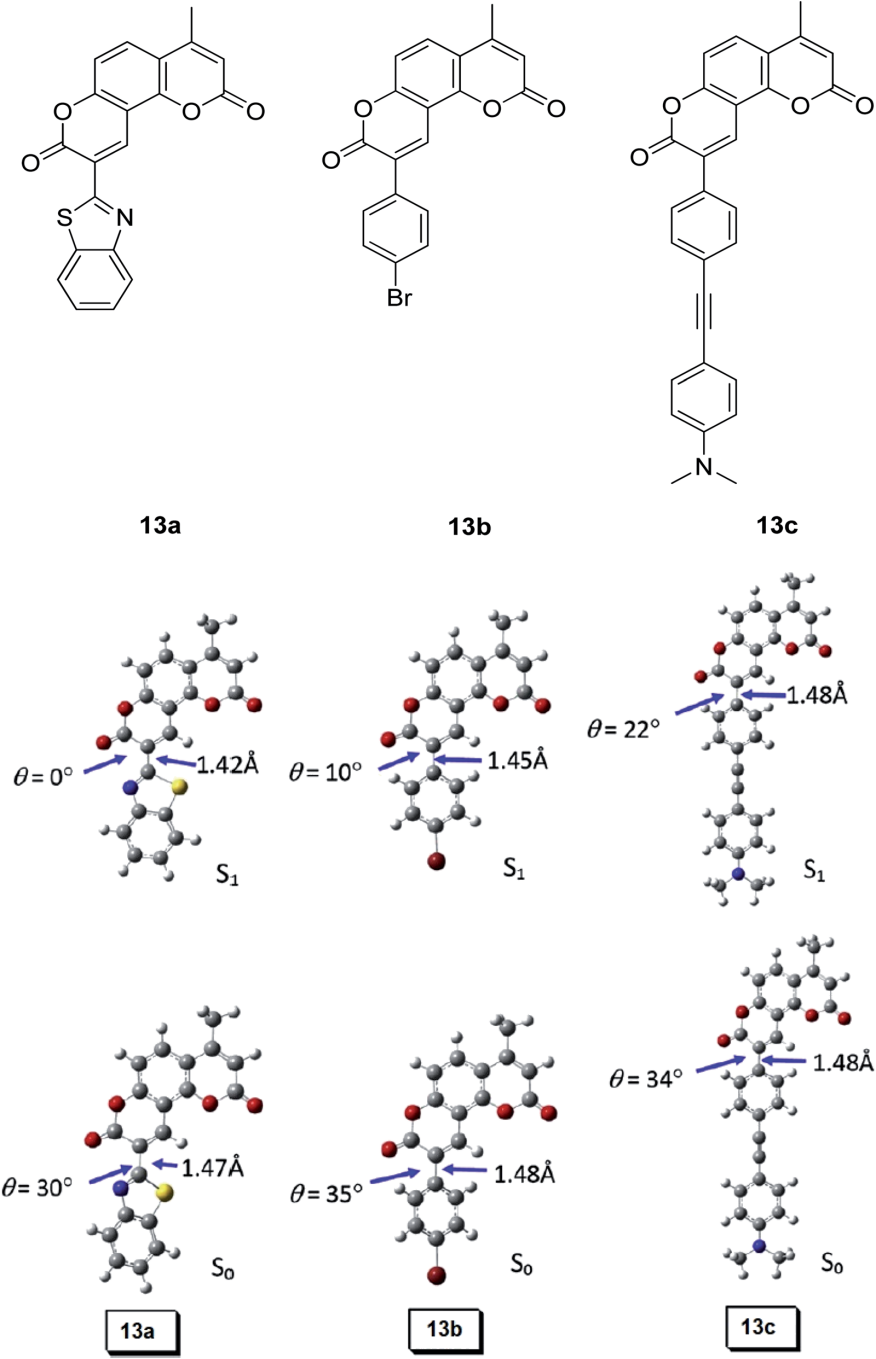
Geometry of the coumarins 13a, 13b and 13c at the ground state (S_0_) and the singlet excited state (S_1_). Toluene was used as a solvent in the calculation. Calculated at the B3LYP/6-31g(d) level with Gaussian 09W. The figure was adapted from ref. 101 with copyright permission from Elsevier.

Synthetic interest in cyanine dye can be associated with its high molar extinction coefficients, good fluorescence quantum yield and easy molecular tuning. In this regard, Sissa *et al.* studied the extension of the polymethine chain and introduction of the cyclohexenyl ring in the central position of the cyanine dye.^100^ It was understood that such molecular modification was responsible for the huge Stokes shift (larger than 0.3 eV) observed in their study. Through investigation of the first excited state and of its geometrical relaxation upon photoexcitation, a cyanine derivative encompassing an amino substitution on the heptamethine spacer was steered to a broken-symmetry ground state, which was subsequently restored upon relaxation in the excited state. This phenomenon caused a large structural re-arrangement of the molecule, and thus justifies the large Stoke shift seen in dichloromethane and DMF solvents. Extension of the π-conjugation framework of coumarin by integrating the benzothiazole moiety has also become one of the renowned strategies to aim for long emission wavelength. Huang and his co-workers devised new coumarin structures that are capable of undergoing structural deformation in their excited state.^101^ Based on the TDDFT method with optimized excited state geometries (S_1_ state), geometrical changes were realized for three of the derivatives (13a, 13b and 13c) in Fig. 18. As for 13a, the dihedral angle between the benzothiazole moiety and fused coumarin moiety changed from 30° in the S_0_ state to a coplanar conformation of 0° in the optimized S_1_ state. Based on this significant geometry relaxation, the energy levels of the LUMO orbital decreased significantly too, giving rise to a large Stokes shift (87 nm) of the compound. Since there was not much difference in the variation of the dihedral angles in 13b, this compound has a lower Stokes shift value of 81 nm. Surprisingly, 13c showed the largest Stokes shift of 144 nm with yellow emission (*λ*_em_ = 555 nm) due to the presence of an acetylide phenyl moiety. In both S_0_ and S_1_ states, both HOMO orbitals were localized on this acetylide phenyl moiety, while the LUMO orbitals were more localized on the fused coumarin moiety. Aside from having geometry changes within the molecule, ICT played an important role in producing the large Stokes shift for this compound.

The interest in the naphthalene-fused BODIPY dye synthesis by Yang *et al.*^102^ has led to the discovery of its asymmetric analogue that exhibits a large Stokes shift and high photostability. By investigating the frontier molecular orbital profiles after geometry optimization using TDDFT, it has been proven again that the geometrical relaxation of the molecule is one of the promising avenues that researchers should consider when designing large or mega Stokes shift compounds. Upon photoexcitation, there was a decrease in the LUMO energy level of the derivative from −2.56 eV (in the Franck–Condon state) to −2.93 eV (in the optimized S_1_ state), which accounts for the large Stoke shift characteristic. Kushida and co-workers also worked on a constraint-induced structural deformation in the excited state, particularly for the initially planarized triphenylborane derivatives.^103^ An important concept in molecular engineering is to provide a structural constraint to the π skeleton. This will restrict the structural flexibility, thereby suppressing nonradiative decay pathways from the electronically excited state. Since boron has a vacant p orbital, π-conjugation can be prompted by linking boron with electron acceptor materials. In Kushida's study, three-methylene-bridged triphenylboranes showed unique excited state dynamics, including plane-to-bowl structural changes, dual emission at 337 nm and 407 nm, and reasonably good fluorescence yield. The bowl-shaped structure in the S_1_ state was accountable for the red-shifted and lower energy emission.

In the case of the inorganic fluorescent dye, the role of structure deformation is responsible for the broadband emission. Applying the same concept as geometry relaxation, hybrid perovskites (NBT)_2_PbI_4_ and (EDBE)PbI_4_ were investigated to undergo structural changes in the HOMO–LUMO transition of the I_3_^−^.^104^ The emergence of such states was associated with emissive traps, leading to large Stokes-shifted luminescence in accordance with the relaxation mechanism typical of trapped charge carriers. These behaviors were also reported in self-assembled organic–inorganic perovskite (C_6_H_11_NH_3_)_2_PbBr_4_ that underwent elastic deformation for exciton delocalization to produce strongly Stokes-shifted emission^105^ and perovskite (*N*-MEDA)[PbBr_4_] that weathered elastic deformation, leading to strong vibronic coupling between excitons and the lattice to induce a Stokes shift of up to 170 nm.^16^ We have concluded the excited state structural or geometrical dynamics that can lead to a distinct gap between the absorption and emission wavelengths based on the reviewed examples involving BODIPY, oxazole, cyanine, coumarin, triphenylborane and some inorganic perovskites.

### 3.4 Hydrogen bonding mediated J- or H-aggregation and aggregation induced emission (AIE)

Another factor that governs the molecule in possessing a large Stokes shift component is the role of the aggregation morphology of the luminescent molecules. Before delving into AIE, the difference between the two types of aggregates should be noted. When dye molecules aggregate and form a concentrated aqueous solution with bound molecular assemblies, their absorption band will shift significantly towards the longer wavelength of the visible spectrum with respect to the monomer absorption band. Such phenomenon is known to be J-type aggregation in honor of Jelley's (J-) and Scheibe's bathochromically shifted aggregation of pseudoisocyanine chloride discovery in the early 1930s.^106^ Conversely, any blue-shifted absorption bands due to the self-assemblies with different arrays (depending on the dipole moments) are termed as H-aggregate (H denotes hypsochromic).^107^ H-aggregate could be one of the contributing factors to providing a large Stokes shift component, but comes with its downside of being non-emissive in most cases unless with several exceptions. Understanding the behaviors of these aggregates provides general guidance for researchers to design and control the aggregation morphology of the luminescent molecules in correspondence to their functionalities.

Concerning the nature of H-aggregates that result from the parallel stacking of molecules with a “side-by-side” orientation, we will look into a report by Ryu *et al.* on the remarkably large Stokes shift that was sourced from H-aggregated cyanine dyes.^108^ The system that they proposed was based on the interaction of the dye molecules with chiral assemblies of a cationic gemini surfactant. The key to obtaining a large Stokes shift molecule and narrow absorption band is to establish a media with the presence of the gemini surfactants (16-2-16 l-tartrate was used in their experiment). With this, the dyes can be electrostatically bound to the surfactants in the form of head-to-head arrangement. In methanol containing 0.2 mM of 16-2-16 l-tartrate, the H-aggregated cyanine dyes is capable of producing a notably large Stokes shift of up to 224 nm. This was in line with a previous study on the red-shifted fluorescence that was set forth to originate from the lower energy exciton state (forbidden state) of the H-aggregate species.^109^ However, Ryu and his group assumed that the H-aggregated cyanine dyes on the 16-2-16 l-tartrate assemblies in their study were converted to an excimer-like state upon photoexcitation. The wicked aggregation-caused quenching (ACQ) effect will also transpire from the presence of a detrimental species, like the excimer and exciplex.^110^ Hence, the judicious utilization of the AIE effect needs to be taken into consideration, while enriching the AIE palette for different applications.

**Fig. 19:**
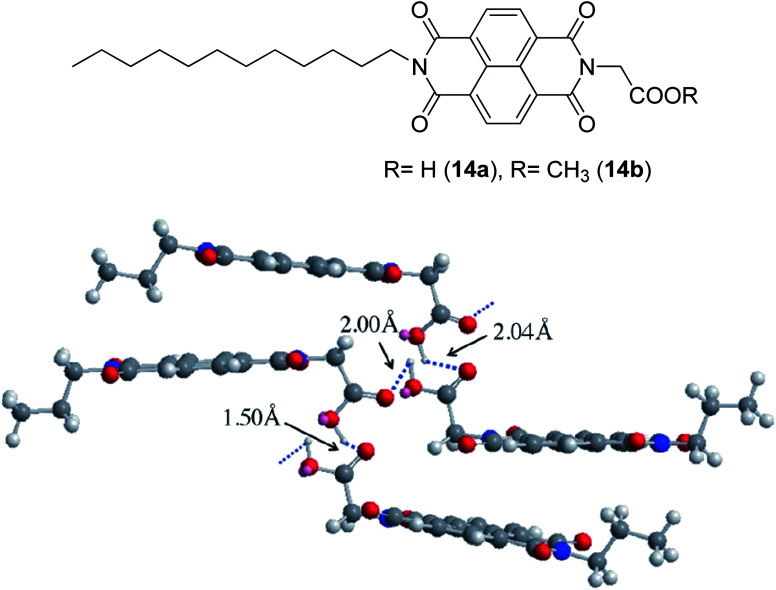
1,4,5,8-Naphthalenetetracarboxdiimide derivatives (14a and 14b) that were synthesized from ref. 111. The lower image shows the energy-minimised self-assembled structure of 14a (for simplicity, the alkyl chain in 14a was replaced by a *n*-propyl chain). The figure was reproduced from ref. 111 with copyright permission from John Wiley and Sons.

As for J-aggregates, they are known to have a small Stokes shift and red-shifted absorption band. However, such claims might vary depending on the structure–property relationships for different types of compounds. For instance, hydrogen bonding mediated J-aggregation has been mentioned by Molla *et al.* in their study on a single component chromophore based on the naphthalenediimide skeletal system.^111^ Compounds 14a and 14b were synthesized to investigate the role of the carboxyl groups that can form a self-complementary hydrogen bonding network, with the long alkyl chain for enhanced solubility in non-polar media. The absorption profile of 14a from polar to a less polar solvent unveiled a concomitant redshift band, but of reduced intensity, which was said to emanate from the J-type π stacking. The control molecule 14b showed no significant changes in its absorption spectra, which ascertained the role of the hydrogen bonds in promoting a red-shifted spectral feature. In a mixture of solvents consisting of 95 : 5 of methylcyclohexane : chloroform, 14a was shown to have various possible hydrogen bonding motifs of the carboxyl functionalities. However, the *syn*–*syn* catemer (Fig. 19) formation was said to be the most probable mode of self-assembly that corroborates the J-aggregation-induced redshift in the absorption and emission spectra. Compound 14a was reported to emit at 545 nm, giving a Stokes shift value of 151 nm, indicating emission from an excimer-like species.

**Fig. 20:**
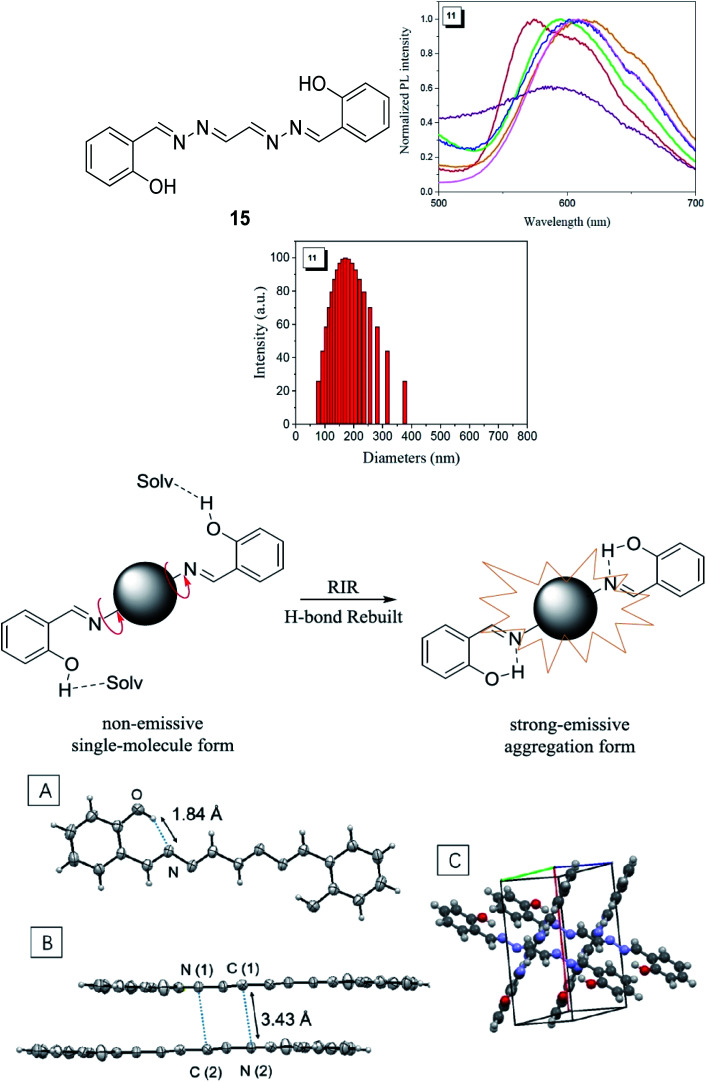
(Top) Chemical structure, normalized PL spectra in different solvents, and distribution of the particle size of compound 15. (Middle) The proposed AIE mechanism of the SSB dye in aggregation form. Solv stands for solvent molecules. (Bottom) Single crystal structure of compound 15. (A) ORTEP drawing with 50% probability ellipsoids and hydrogen bond geometry; (B) side view and short contact geometry; (C) a unit cell. The figures were reproduced from ref. 112 with copyright permission from Elsevier.

The behaviors of both J- and H-aggregates were proven not necessarily rigid, but may have alterations based on previous studies. It is undeniable that there is still a lot of effort needed to decipher light-emitting processes at the molecular and photophysical levels. Moving onto a more general aggregation, recently in 2018, Gong and his group designed red-emitting salicylaldehyde Schiff base (SSB) dyes with AIE behaviors and large Stokes shifts.^112^ When the SSB dye (compound 15) was tested in a tetrahydrofuran : water mixture, it displayed a strong yellow to red fluorescence (up to 617 nm) and large Stokes shift (up to 152 nm). A higher water volume fraction within the solvent mixture led to fluorescence enhancement and high absolute fluorescence quantum yield (up to 70-fold when the water fraction reached 90%). To scrutinize the aggregation state of compound 15, dynamic light scattering (DLS) was used to determine the particle size. Compound 15 having the smallest particle size (171.59 nm on average) was interrelated to the restriction of intramolecular rotation, hence possessing appreciatively high AIE activity (170.0). This coherently describes the recovered formation of intramolecular hydrogen bonds between the dye molecules (Fig. 20) that led to the notably large Stokes shift. Profound information was retrieved from the analysis of the single crystal structure of compound 15, which explains the AIE behavior in more detail. The distance of O–H⋯N was 1.84 Å, proving the presence of stronger intramolecular hydrogen bonding within the molecule that also facilitated the ESIPT process. Schiff base derivatives are known to offer a myriad of applications in provision of its large Stokes shift and other propitious photophysical properties.^113^ Both AIE and ESIPT photophysical mechanisms are responsible for the large Stokes shift of the molecule.

In the same year, Song *et al.* proposed a novel AIE plus ESIPT properties fluorescent probe based on salicylaldehyde azine (SA) to overcome the small Stokes shift and aggregation-caused quenching (ACQ) problems that arise when accumulated in cell^114^ and biological media. However, the focus of the discussion is the mother molecule that is capable of producing a large Stokes shift of up to 148 nm. Like Gong's study, they reported that the AIE character of the fluorescent dye can be evaluated when investigating the parameter of the fluorescence wavelength and intensity with the increase of water volume fraction. In the solvent of the PBS buffer-DMSO system, the SA's fluorescence intensity increased sharply when the water volume fraction exceeded 80%. Aggregates formed in the PBS buffer (“poor solvent” as termed by Tang *et al.*, indicating the poor solvating power with increased water volume fraction^115^) also produced a red-shifted fluorescence, establishing a significant difference in the excitation and emission wavelengths upon cysteine recognition. A coumarin-based AIE-active red emission has been developed by Yan *et al.* by exploiting the capacity of the Schiff base emerging from aldehyde and amino groups.^116^ The fluorescence behavior in water : DMF (*f*_w_, vol%) mixtures showed enhanced aggregation with a higher water fraction. In pure DMF (*f*_w_ = 0%), the coumarin Schiff base showed very weak fluorescence, whereas when *f*_w_ was varied from 10% to 40%, the emission intensity decreased along with a slight red shift of the emission peak as the solvent polarity increased. The behavior implied the presence of the TICT process, which caused a fluorescence decrease. The highlight of their study was when *f*_w_ > 40%, and the maximum emission wavelength shifted from 540 nm to 640 nm alongside an evident increase of fluorescence intensity. This red shift was ascribed to aggregation, which induced an additional mechanism responsible for the large Stokes shift (245 nm) and enhanced emission intensity. Based on the particle size distribution study (DLS), one size distribution peak was observed at 20 nm (*f*_w_ = 40%) and two size distribution peaks were detected at 494 nm and 9.8 μm, respectively (*f*_w_ = 60–70%). At *f*_w_ = 90%, the size of coumarin Schiff base particles shifted to larger values, thus consolidating the AIE character of the molecule. Yan's coumarin Schiff base reached a Stokes shift value of up to 146 nm, 161 nm, and 199 nm in toluene, ethyl acetate and ethanol, respectively. In thin film form, the material was capable of giving red emission with 26.3% quantum efficiencies.

Therefore, the next aspect to explore is researchers' strategies to molecularly design AIE-bearing compounds and/or endow AIE molecules with self-assembly. This question has been elaborated by Wu *et al.*, in which they highlighted that AIE molecules would normally be nonplanar with: (1) propeller-shaped molecules, (2) butterfly or V-shaped molecules, or (3) rotatable linear molecules.^117^ On these accounts, important approaches to envisage include the presence of intermolecular interactions, like π–π stacking, hydrogen bonding, van der Waals forces and hydrophobic effects. Based on the reviewed structural cores (cyanine, naphthalenediimide, salicylaldehyde and coumarin Schiff bases and salicylaldehyde azine), it is evident that the prerequisite to supplicate AIE is by accommodating a suitable solvent system and condition for molecules to start to agglomerate and fluorescence. Accompanying mechanisms like ESIPT and ICT are also liable for the preferred photoluminescence properties of AIE molecules.

### 3.5 Interchange between monomer and excimer fluorescence

Tuning fluorescent compounds to acquire a large Stokes shift characteristic is indeed challenging, especially when factors that regulate the mechanistic aspect imperil other properties that are demanded in white light generation or other lighting applications. Such feature peculiarity is reinforced in the excimer formation of a molecule. Excimers are excited atomic or molecular dimers (A*–A) that are normally derived from aromatic molecules, noble gas atoms, monatomic vapors, and activator ions in alkali halide crystals.^118^ They are fluorescent in nature, with an emission band produced at lower energy than its monomer as a result of the proximity between multiple chromophores.^119^ An excimer system typically combines strong lateral bonding (covalent bonding) with weak interlayer binding.^120^

Highly conjugated molecular systems (*e.g.*, naphthalene, pyrene, phenanthrene, anthracene, among others) are great excimer contributors due to the facile π–π interaction. The pyrene series have been reported to produce a long emission wavelength and Stokes shift of more than 130 nm, suggestive of an excimer emission when incorporated on the DNA backbone as a fluorescent label.^121^ They showed an increase in the fluorescence quantum yield upon the addition of more than one fluorophore to encourage close interactions. This behavior has a perquisite when assessing for white light emission that comes from a mixture of red, green, and blue fluorophores. In general, when the number of monomers increased, the excimer-to-monomer ratio will markedly increase, and this feasibly enhances the fluorescence peak intensity. Kobori *et al.* also reported a remarkably large Stokes shift (230–330 nm) excimer-forming candidate based on a cyanine dye and FRET-based pyrene probe.^122^ In their study, conjugation between the oligoribonucleotides and cyanine dye gave rise to a pyrene excimer in the excited state that contributed to the long-wavelength emission. Other research laboratories that worked on the pyrene series as an excimer emission characterized by a large Stokes shift extended the pyrene core with moieties like pyridinium and borane.^37,38,123^

Luminescent alkali halide crystals exhibiting interesting photophysical and photochemical behaviors have been outlined by Li *et al.* using dicyanoaurate, dicyanoargentate and dicyanocuprate as the activator ions.^124^ Closed-shell metals like gold can repel each other due to fully occupied valence orbitals, inducing a form of weak interaction called the aurophilic interaction. On this basis, they developed a ligand-unsupported Ag(i) complex, T1[Ag(CN)_2_], that can form excimers *[Ag(CN)_2_^−^] when in bulk condition. It was discovered that the bond distance between two Ag atoms was shorter in the excited state, while their binding energy and overlap population increased significantly in the same state. These resulted in a stable interaction between two monomeric units in the excited state (*i.e.*, stable dimer). A kinetic study conducted suggested that there was a fast energy transfer between the *[Ag(CN)_2_^−^]_*n*_ excimers and decrease in the HOMO–LUMO band gap, which promoted a longer wavelength emission and large Stokes shift. Similar manners were realized on Au(CN)_2_^−^ and Cu(CN)_2_^−^, but each possessed different relativistic effects.

Hence, it is conspicuous that when screening for an organic molecular architecture for excimer emission, π–π interactions are substantially essential within the chromophore. Extending the conjugation of the fused aromatic ring, therefore, is a rational design to enhance the number of non-covalent bonding interactions among the molecules.^125^ The concerns among researchers in the field are devoted on such systems that possess good solution processability, relatively good fluorescence quantum yield, good charge transport and other significant optoelectronic properties. As for inorganic approaches, continuous efforts toward varying the ligands on closed-shell metals with d^10^ electronic configuration have been done.^126^ With the wide spectrum of metal donors that exist nowadays, it is anticipated that unique photo-induced dynamics in the excited state of both solid and solution forms will be prompted, especially long wavelength emission of more than 600 nm that comes with a large Stokes shift property and high emission intensity.

### 3.6 Host–guest interaction

White light emitters that originate from single molecules are promising due to their high color stability and tunability. In this section, we emphasize factors that inhibit the pathways of non-radiative relaxation as much as possible *via* supramolecular interaction. The said interaction focuses on the host–guest relationship between large molecules containing heavy atoms (Br, Cl, and others) and heteroatoms (O, S, N, and others). Host–guest molecules can generate the room-temperature phosphorescence (RTP) that is well known to display a long emissive wavelength, lifetime, and large Stokes shift. Ma *et al.* highlighted the importance of the host–guest assembling and supramolecular polymer system that manifested the described characteristics.^127^ Conventionally, the crystalline packing would be an efficient avenue to establish a rigid environment of the molecules for enhanced RTP, but non-crystalline materials also have very recently caught the attention of researchers.^128,129^ These amorphous metal-free organic materials could potentially replace the rapid utilization of scarce and toxic metals in stride to develop large Stokes shift molecules.

**Fig. 21:**
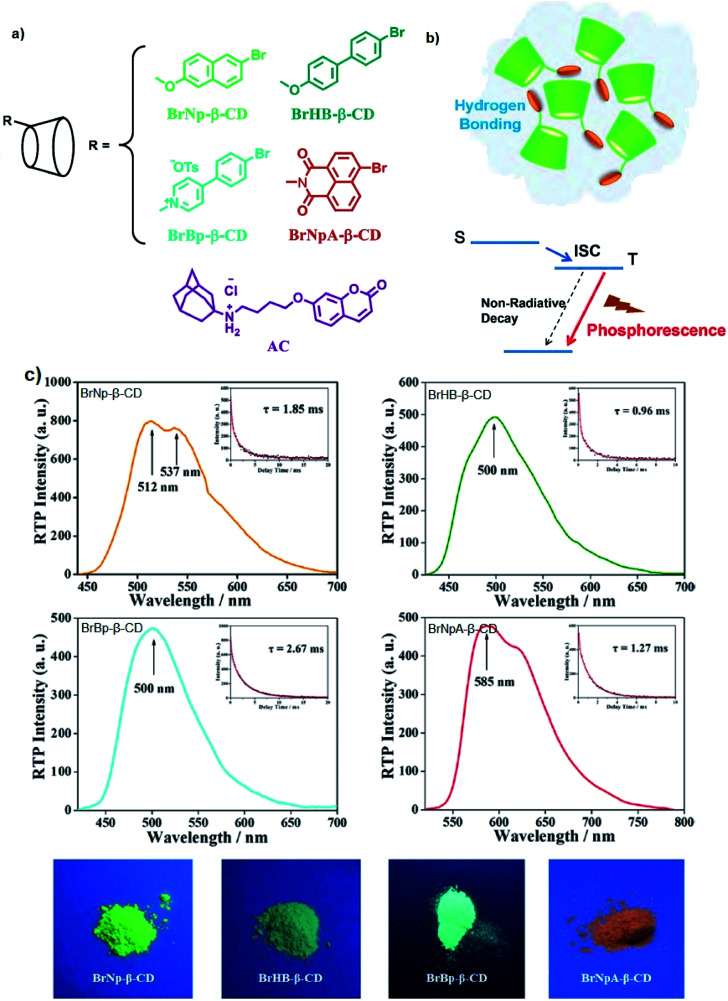
(a) Molecular structures of RTP emissive cyclodextrin derivatives and the fluorescent guest molecule AC; (b) schematic representation of the phosphorescence emission; (c) RTP emission spectra in amorphous solid states with *λ*_ex_ = 285, 300, 350, and 365 nm, respectively. Luminescent delay lifetime (insets) and photographs of the solid powder of the four RTP-emissive CD derivatives. The figures were adapted from ref. 130 with permission. Copyright 2018 American Chemical Society.

β-Cyclodextrin (β-CD) has been employed as a macrocycle host embedding the phosphor moieties as host–guest strategies. Several reports have been discussed about the unprecedented potentials of β-CD for having the hydrophobic cavity that can encapsulate guest molecules for RTP.^92,130^ Li *et al.* synthesized different guest molecules to be incorporated onto the hydrophobic pockets of β-CD in order to attain multicolor emission *via* fluorescence-phosphorescence strategy.^130^ In their study, four different phosphor materials were synthesized, 6-bromo-2-naphthol (6-BrNp), 4-bromo-4′-hydroxybiphenyl (4-BrHB), 4-(4-bromophenyl)-pyridine (4-BrBp) and 4-bromo-1,8-naphthalic anhydride (4-BrNpA) (Fig. 21). The Stokes shifts of RTP emission for BrNp-β-CD and BrNpA-β-CD exceeded 230 nm and 220 nm, respectively, while both BrHB-β-CD and 4-BrBp-β-CD reached over 200 nm. All can be regarded as having large Stokes shifts. These compounds could be RTP emissive even in amorphous solid states, which makes them convenient for preparation and practical utility. The key approach to RTP emission with the presence of β-CD could be attributed to an external factor, such as suppression of non-radiative decay *via* increased of intermolecular hydrogen bonding atmosphere. Extending the guest molecule based on adamantine derivatives within the host formed a supramolecular complex that could be fine-tuned from yellow to purple emission, including white light.

**Fig. 22:**
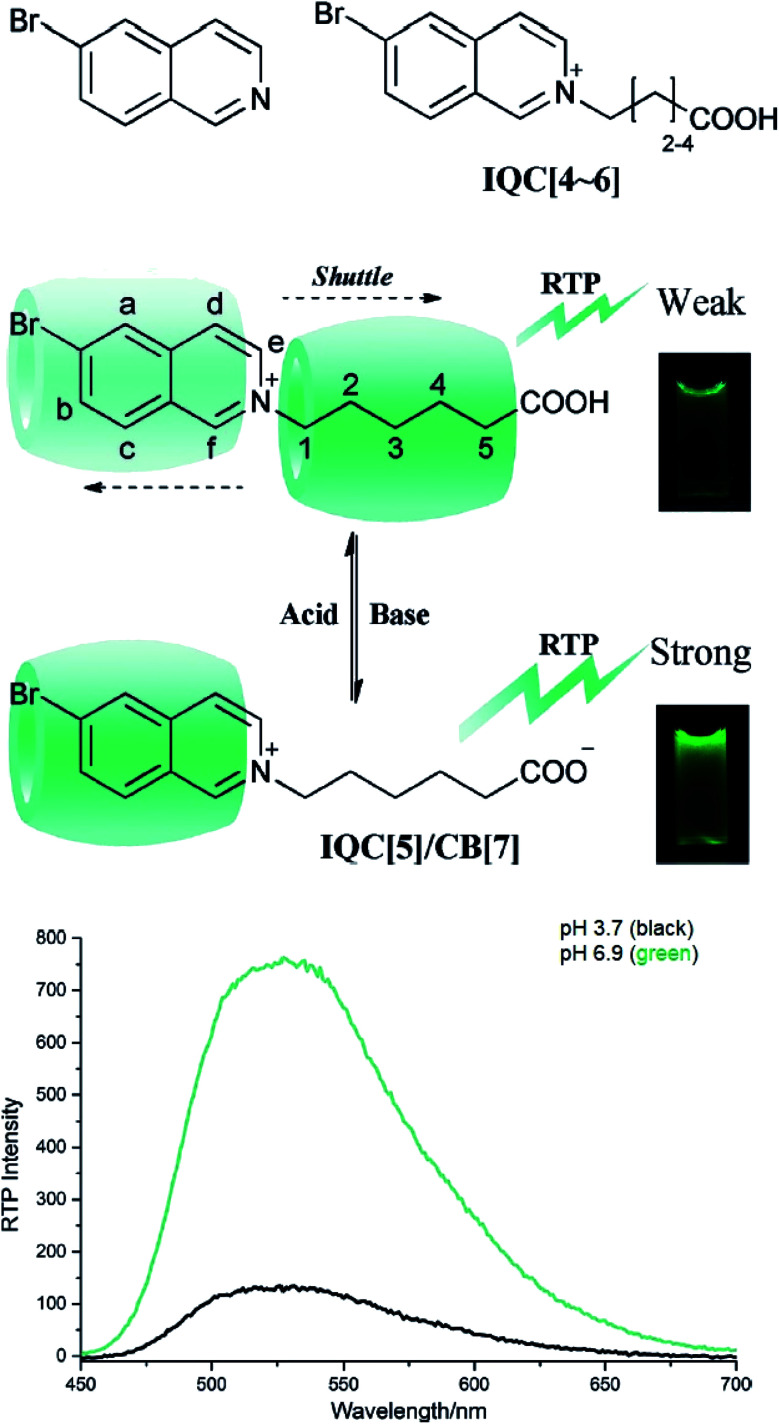
Structures of the guests and the reversible inclusion phenomenon of the complex system. Photographs show the aqueous solution of the complex upon illumination with a handheld UV lamp (254 nm). Below shows the RTP spectrum of the host–guest system in water with *λ*_ex_ = 300 nm. The figures were reproduced from ref. 131 with copyright permission from John Wiley and Sons.

In another work by Gong and his research group, cucurbit[7]uril, (CB[*n*]) host was complexed with a 6-bromoisoquinoline derivative guest to form a pH-controlling molecular shuttle for intense RTP emission, which also gave an appreciably large difference in their excitation and emission wavelengths.^131^ Similar to β-CD, CB[*n*] is a barrel-shaped macrocycle host that bears a hydrophobic cavity to fabricate stable inclusion complexes with phosphor molecules. Since heavy atoms are capable of enhancing the intersystem crossing (ISC) rate of a chromophore, yielding a population of the triplet state and enhanced RTP emission, 6-bromoisoquinoline derivatives were selected as the guest molecules.^132^ Experimental results revealed that a very stable host–guest system could be achieved in a non-acidic environment. The interesting feature of Gong's CB–RTP complex was the fact that upon illumination of UV light, the complex produced a considerably long emission wavelength near the green region, giving a Stokes shift value of more than 200 nm (Fig. 22). This emerged from the molecular switching of CB[7] *via* protonation or deprotonation of the terminal carboxyl group in water. An early article on the emission of isoquinoline derivatives showed a narrow to medium Stokes shift.^133^ Hence, it is evident that complexation with the host molecules unraveled a new route for future study that is to employ macrocyclic molecules to encapsulate “threads” of guest molecules, establishing a rigid environment in favor of better efficiency of the phosphorescence emission. However, a practical solution when employing host–guest recognition for white light in light-emitting diodes applications is to be investigated further. This is because most of the supramolecular entities have good solution processability and decent quantum efficiencies in polar solvents (*i.e.*, water), but insufficient data have yet to be acquired to understand their behaviors in nonpolar media.

### 3.7 Metal–ligand charge transfer (MLCT)

**Fig. 23:**
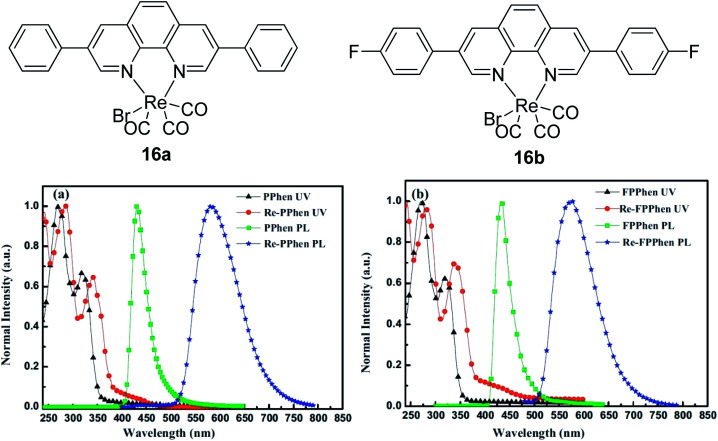
Molecular structure of rhenium(i) complexes (16a and 16b), and their absorption and emission spectra in dichloromethane. (PPhen = 3,8-diphenyl-1,10-phenanthroline; Re-PPhen = 16a; FPPhen = 3,8-bis(4-fluorophenyl)-1,10-phenanthroline; Re-FPPhen = 16b). The figure was adapted from ref. 134 with copyright permission from Elsevier.

For inorganic molecules, MLCT plays a crucial role in expressing a large Stokes shift value *via* phosphorescence by means of harvesting both singlet and triplet excitons. Therefore, many research groups have aimed the advancements of light-emitting materials based on metals like iridium, platinum, osmium, rhenium, and copper. Li and co-workers have brought forward novel rhenium(i) complexes with large Stokes shift (16a and 16b) (Fig. 23).^134^ Ligands based on decorated 1,10-phenanthroline were selected to bind to the central metal Re(i), corresponding to their bidentate chelating nature. The study demonstrated that both 16a and 16b were capable of displaying remarkably large Stokes shifts (>240 nm), which were attested to originate from MLCT processes. A deeper insight has shown that the absorption peaks of 16a, located at 287 nm and 342 nm, were allocated to spin-allowed ^1^π–π* ligand-centered electronic transitions. The said ligand in this compound 16a is 3,8-diphenyl-1,10-phenanthroline (PPhen). The extended absorption bands from *ca.* 380 nm to 550 nm were tentatively attributed to ^1^MLCT and ^3^MLCT, respectively, implying movement of electrons from dπ(Re)→π*(ligand). Compound 16b possessed absorption band features similar to that of 16a, but with more intense MLCT bands. Emission wavelengths of both compounds inherently appeared from *ca.* 575 nm to 582 nm, which proved the indispensability of the central metal to significantly red shift the photoluminescence spectra (from comparison with free ligand). The large Stokes shift also could be sourced from the structural changes that occur in the excited state upon delocalization of electrons between the metal and ligands that eventually relate back to the phenomenon of MLCT.

Copper complexes caught the heed of researchers very recently as well, owing to the intersystem crossing from singlet to triplet excited states in favor of high efficiency light emission. Zhang and co-workers surmounted the 25% internal quantum efficiency limit in fluorescent materials by introducing Cu(i) complexes with different phosphine and phenanthroline ligands in the solid state.^135^ The absorption maximum of the complexes ranged from 365–386 nm, attributed to the low-lying MLCT band, whereas the emission for different derivatives were *ca.* 504–555 nm. These indefinitely opened a new path of large Stokes shift (>138 nm) materials based on the phosphorescence phenomenon of Cu(i) complexes, which are inexpensive and non-toxic to the environmental matrices. It is noteworthy to highlight that the presence of bulky alkyl ligands in positions 2 and 9 of the phenanthroline ligand led to blue-shifted emission. This was due to the sterically hindered structural deformation of the overall complex in the MLCT state, which narrowed the energy gap between the excited and ground states. Hence, care should be taken when screening for ligand candidates to bind to the central metal, whereby characteristics of rigidity and bulkiness need to be compromised. Another Cu(i) complex useful for a large Stokes shift has been reported by Singh and co-workers using 2-mercaptobenzimidazole as ligands.^136^ Their compound was capable of depicting strong emission at 598 nm from an excitation of 300 nm, giving a Stokes shift value of 298 nm. Strong emission arose from MLCT when the 2-mercaptobenzimidazole ligands had more tendency to withdraw electrons from the copper(i) metal upon complex protonation in a solvent system of tetrahydrofuran : water.

In 2019, AlAbbad and his group recently scrutinized the *trans* influence and substituent effects on the HOMO–LUMO energy gap and Stokes shift component in ruthenium mono-diimine derivatives.^137^ The push–pull effect in the context of the metal complex was sourced from the strong oxidant of the Ru metal. Electrons transfer from the t_2_g orbital of Ru to the low-lying π* molecular orbital of the 2,2′-bipyridyl ligand, invoking a singlet ground state (S_0_) to singlet metal-to-ligand charge transfer (^1^MLCT). Emission of the ^1^MLCT band for the Ru complex was known to produce a large Stokes shift (∼150 nm) and maximum wavelength reaching the NIR region of the spectrum. The long-lived intensity decay, typically from 100 ns to 10 μs, was assigned to the triplet state ^3^MLCT emission. If we are to compare the emission wavelength of the Ru complexes designed by AlAbbad *et al.*, it is apparent that the band red-shifted for the Ru metal that accommodated stronger electron withdrawing ligands. Ru connected to the 4,4′-dicarboxylic-2,2′-bipyridyl ligand had the longest emission wavelength at 639 nm, followed by Ru linked to a bis-4,4′-(*N*-methylamide)-2,2′-bipyridyl ligand at 582 nm. The most blue-shifted emission of (514 nm) Ru complex held the ligand bis-4,4′-(methyl)-2,2′-bipyridyl, which is the strongest electron donor group. From these examples, we have clearly depicted that the MLCT character can tune the Stokes shift value. Therefore, selecting a metal–ligand that can promote intersystem crossing to an excited triplet state can widen the gap between the absorption and emission bands.

## 4. Classification of Stokes shifts by narrow, medium and large (mega)

Since there were many reported fluorescent materials with varying components of Stokes shift, we would like to propose nuances or definite categories of the Stokes shifts so that future facile studies based on these characteristics can be referred by researchers when evaluating different types of molecules in specified fields of application. In this section, only selected organic compounds, including some that have been mentioned earlier in this review, will be listed since we could not accommodate all reported fluorescent compounds with varying backbone and skeletal system. The essence of the selected skeletal groups is well known to produce near infrared (NIR) emission that is especially important for many optoelectronic devices. NIR or red emission is highly demanding and ubiquitous for the generation of white light-emitting diodes.

Such selections are outlined to show that chemical modification of molecules and altering the chemical environment can substantially affect the photophysical properties and Stokes shift value of the compounds. With that, it is undeniably that for different skeletal groups, a continual and extensive investigation of this area requires further devotion in order to obtain thorough experimental and theoretical data for a more comprehensive referral. The skeletal groups selected are based on boron dipyrromethene (BODIPY), coumarin, cyanine, perylene, squaraine, and rhodamine molecules. Table 2 lists the categories of narrow, medium, and large (or termed as “mega” when it is exceptionally large) Stokes shifts based on the differences between the excitation and emission wavelength, and some examples of the chromophore. Table 3 tabulates the photophysical properties of the discussed NIR chromophores, including absorption and emission wavelength, fluorescence quantum yield and fluorescence lifetime.

**Table 2:** Category of Stokes shifts and examples. (DMF: dimethylformamide; CH_2_Cl_2_: dichloromethane; EtOH: ethanol; MeOH: methanol; PBS: phosphate-buffer saline; CHCl_3_: chloroform; PhMe: toluene; DMSO: dimethyl sulfoxide)

Narrow Stokes shift, Δ*λ* ≤ 50 nm	Medium Stokes shift, 50 nm < Δ*λ* < 100 nm	Large or mega Stokes shift, Δ*λ* ≥ 100 nm	References
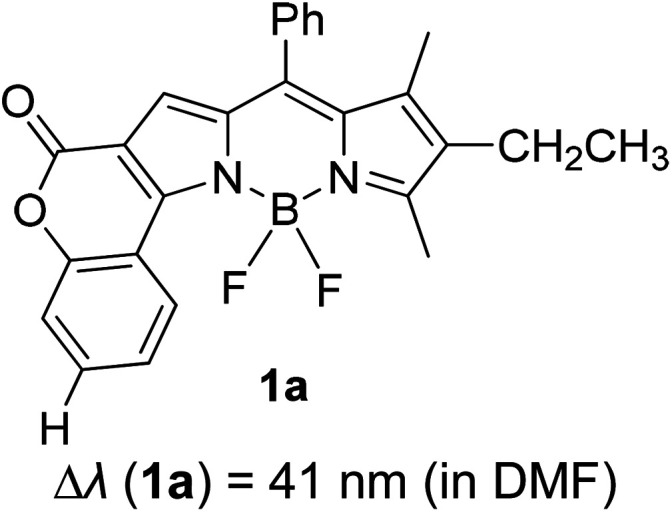	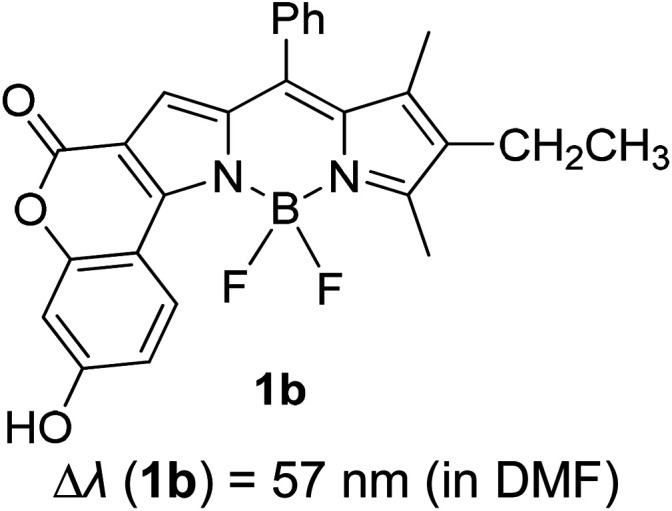	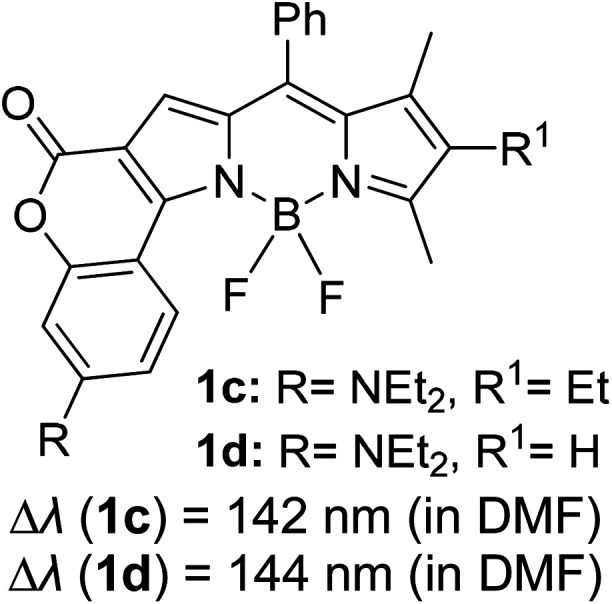	40
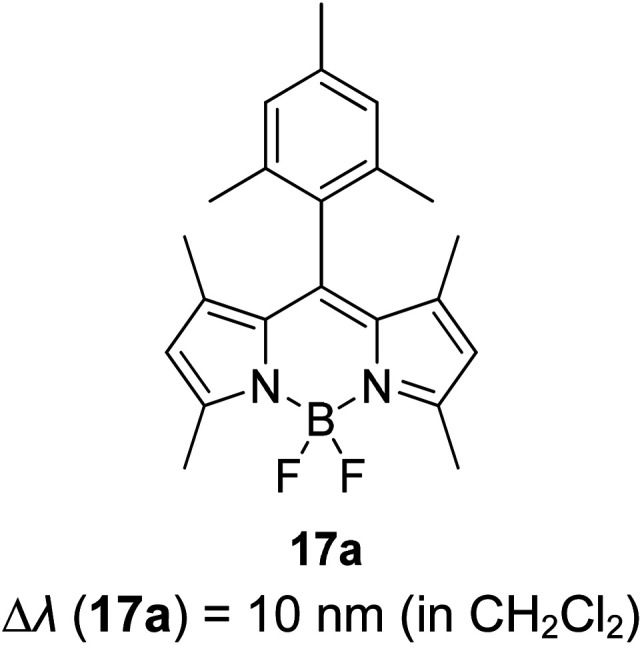	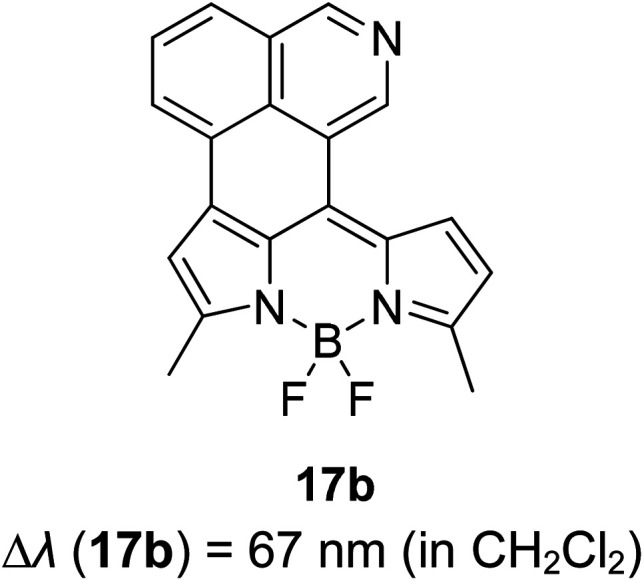		138 and 139
		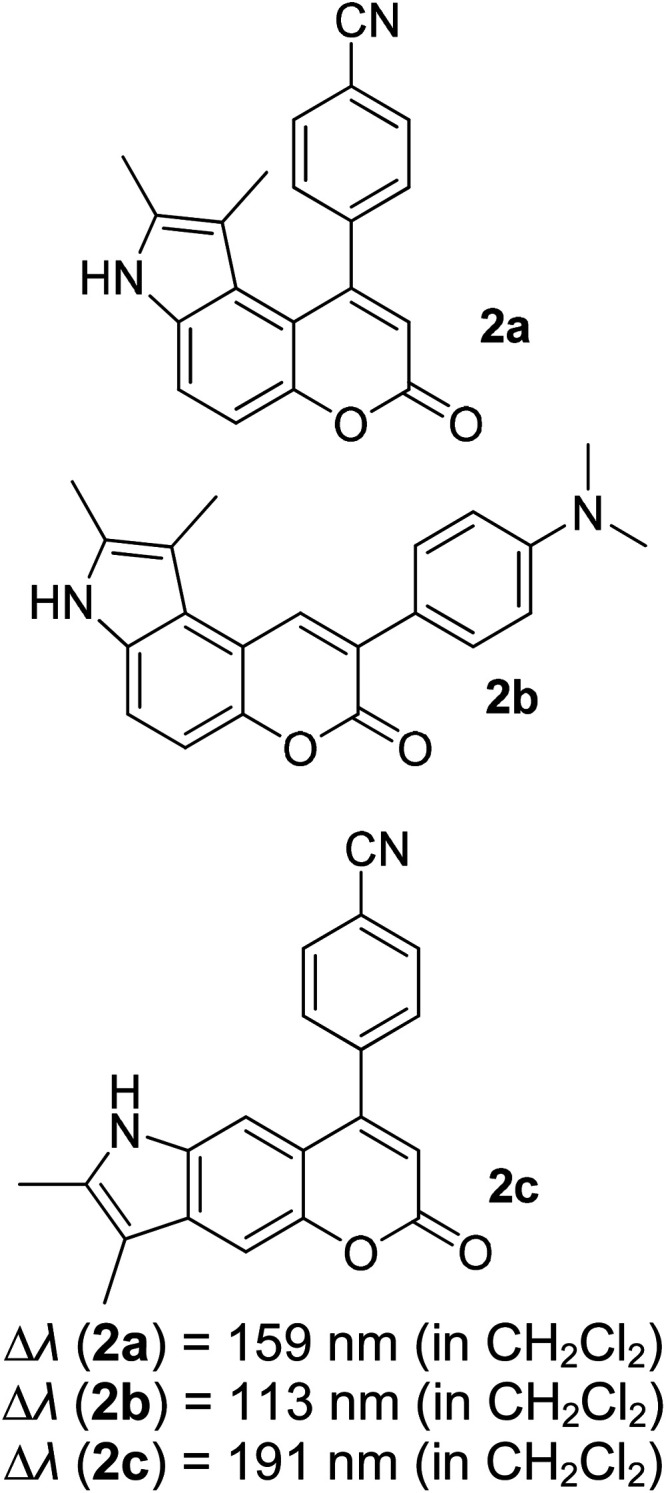	43
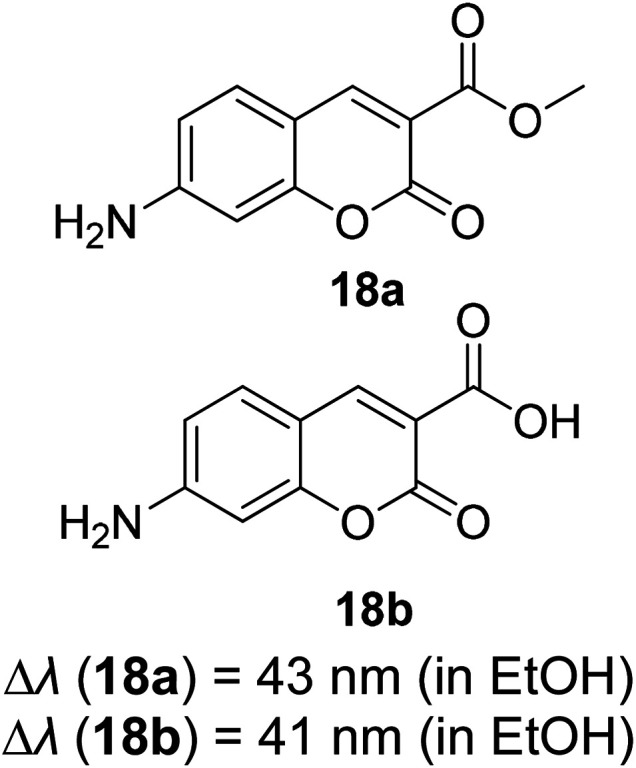	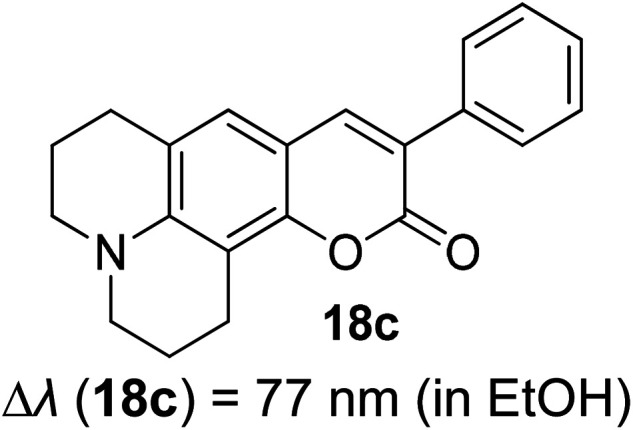	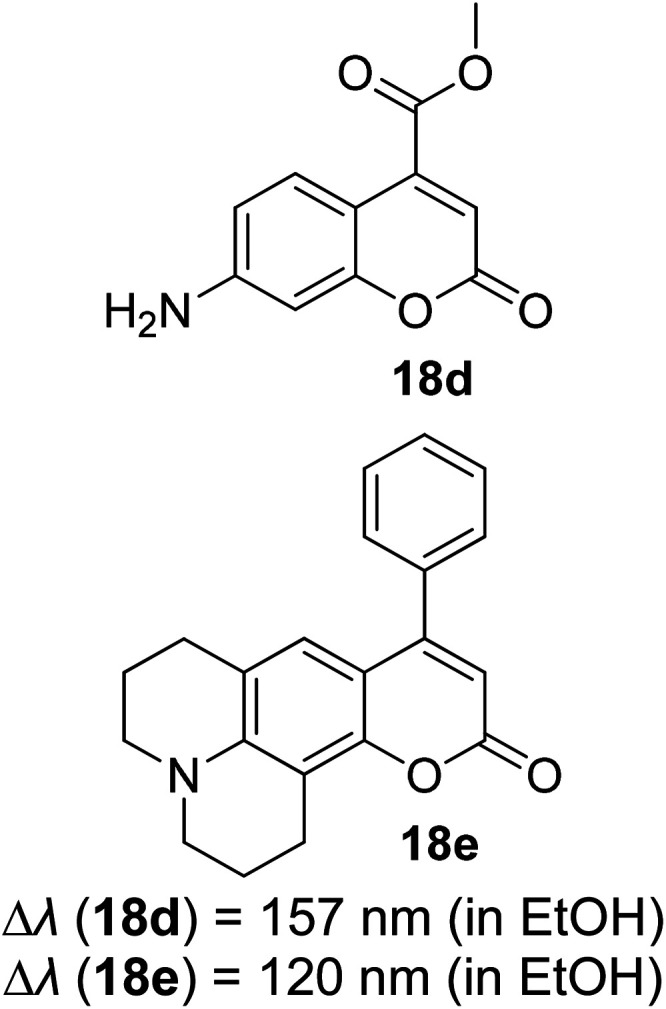	44
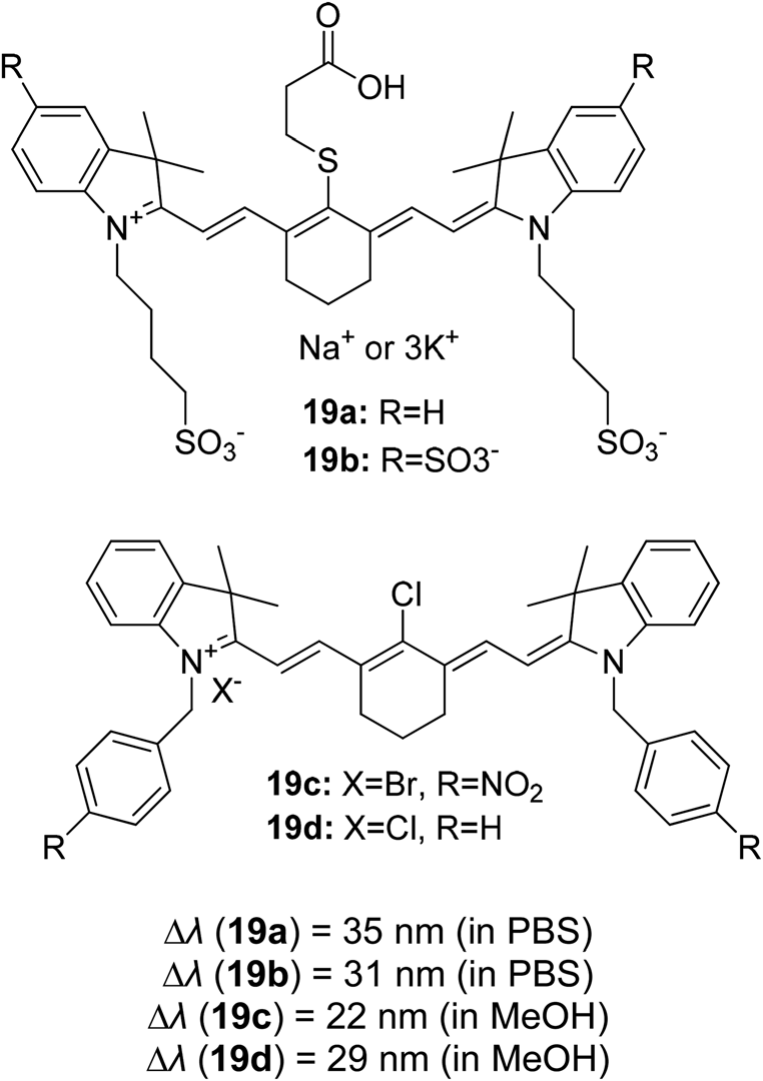	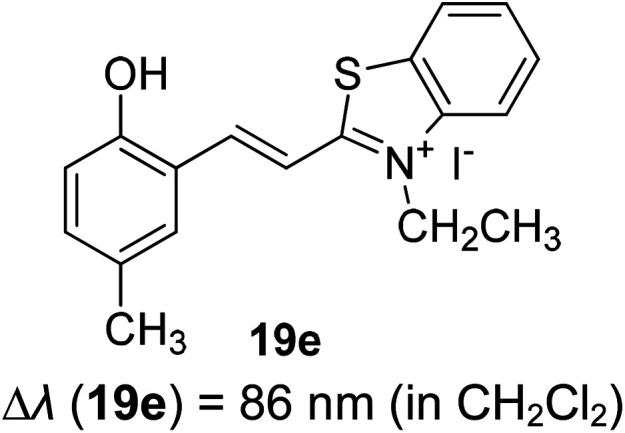	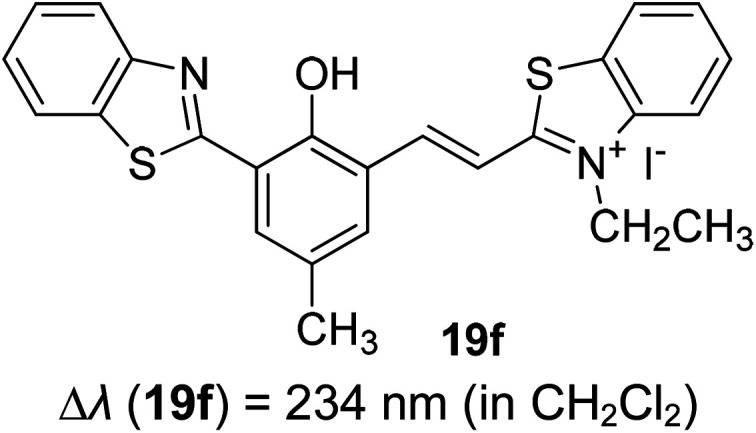	140 and 141
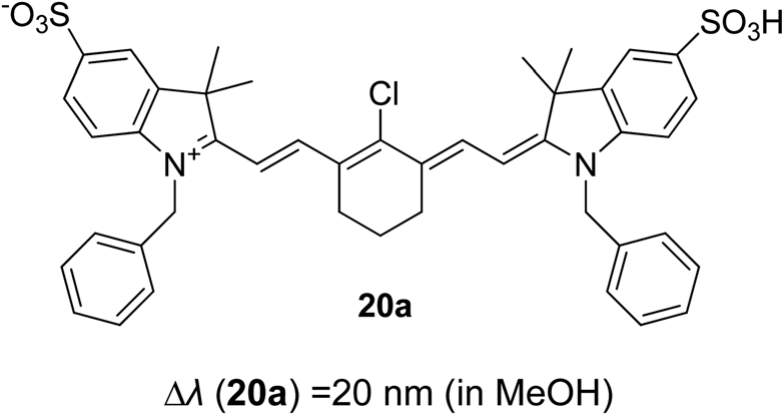		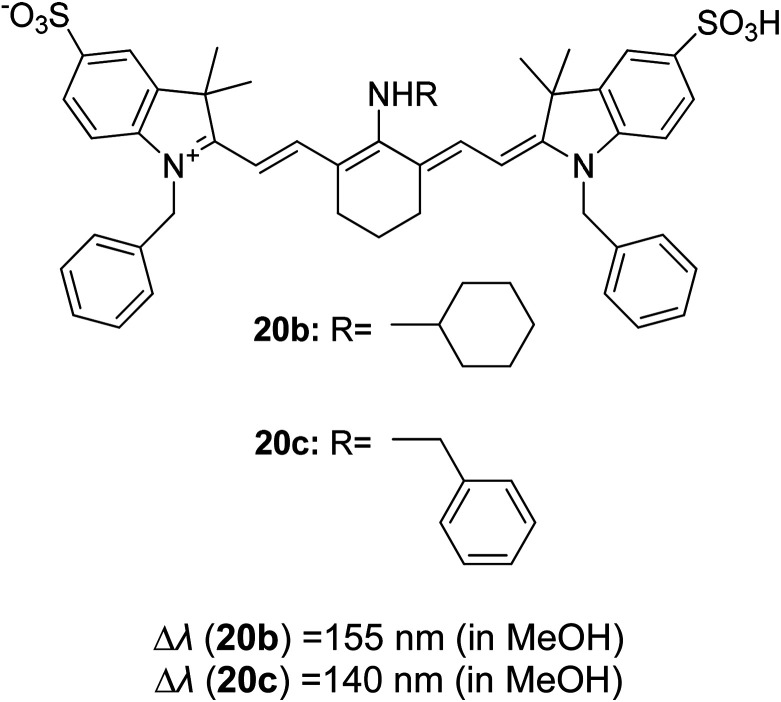	142
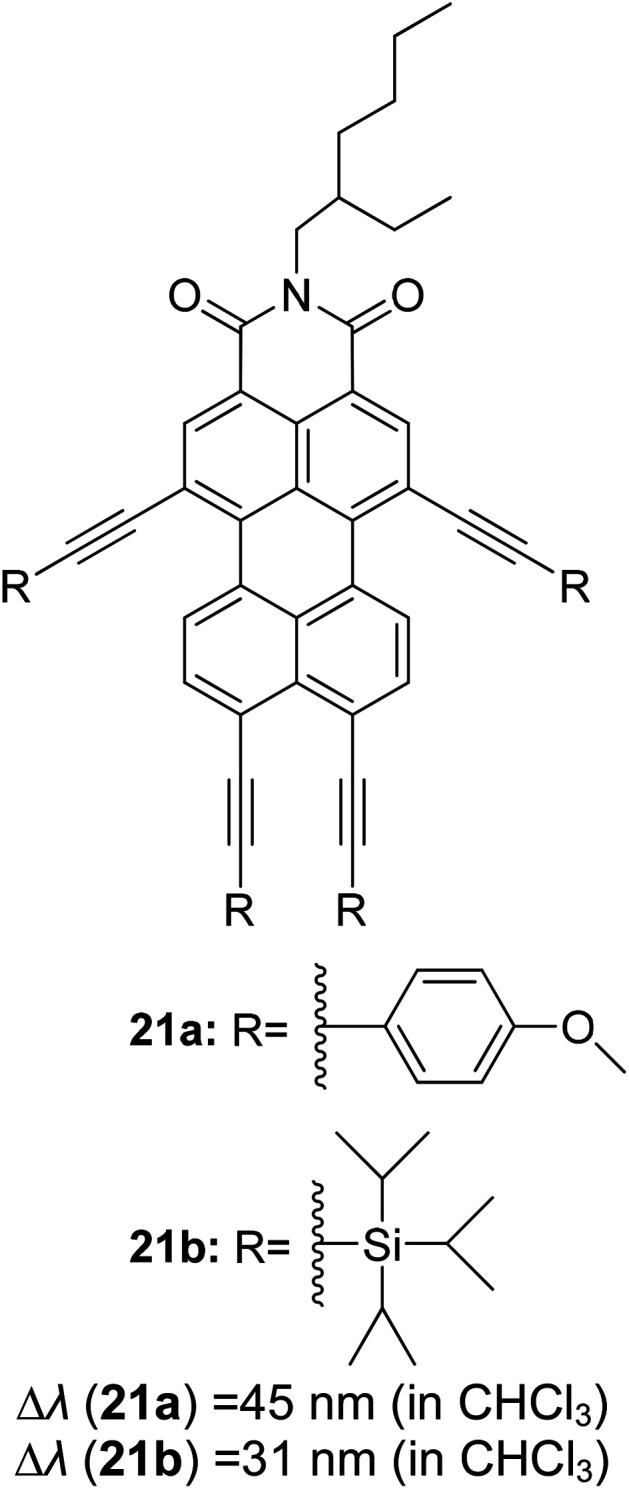		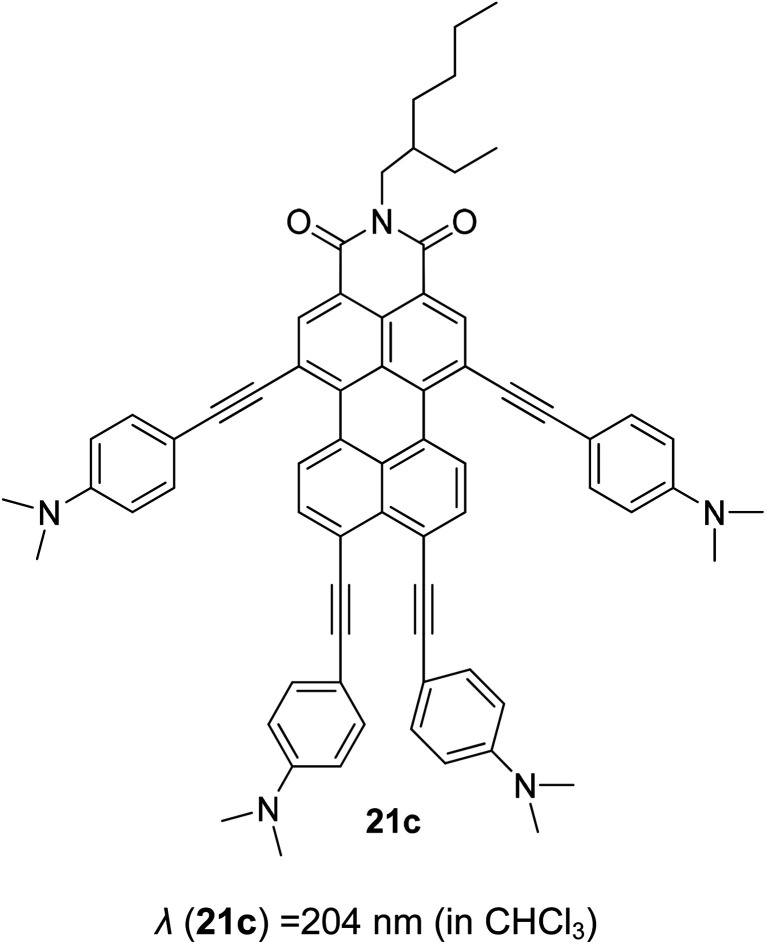	143
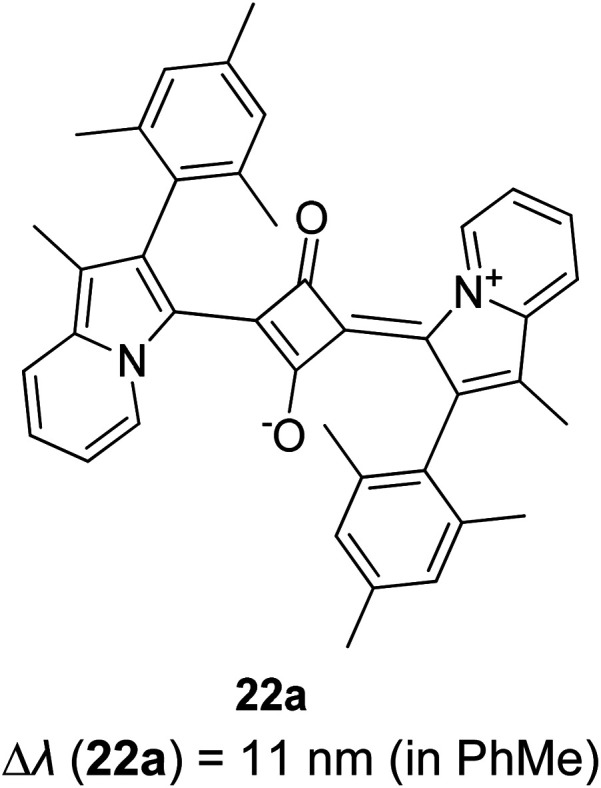	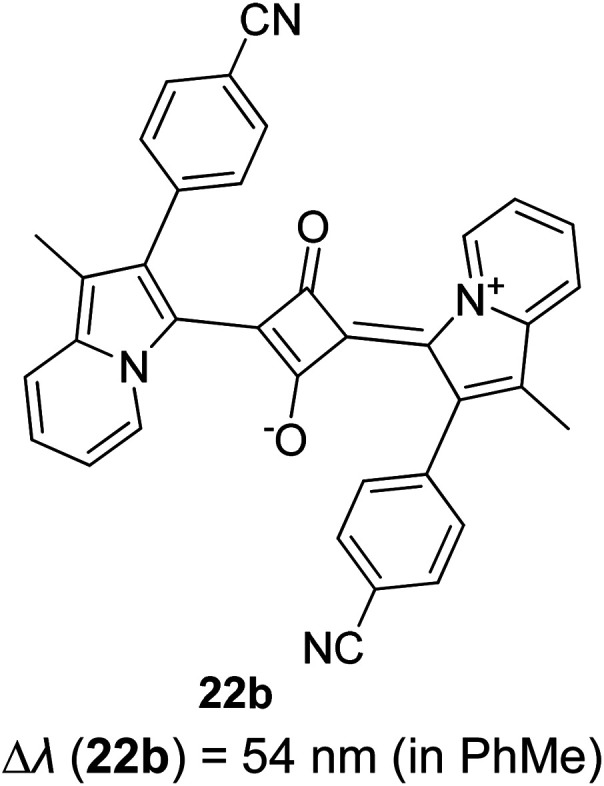	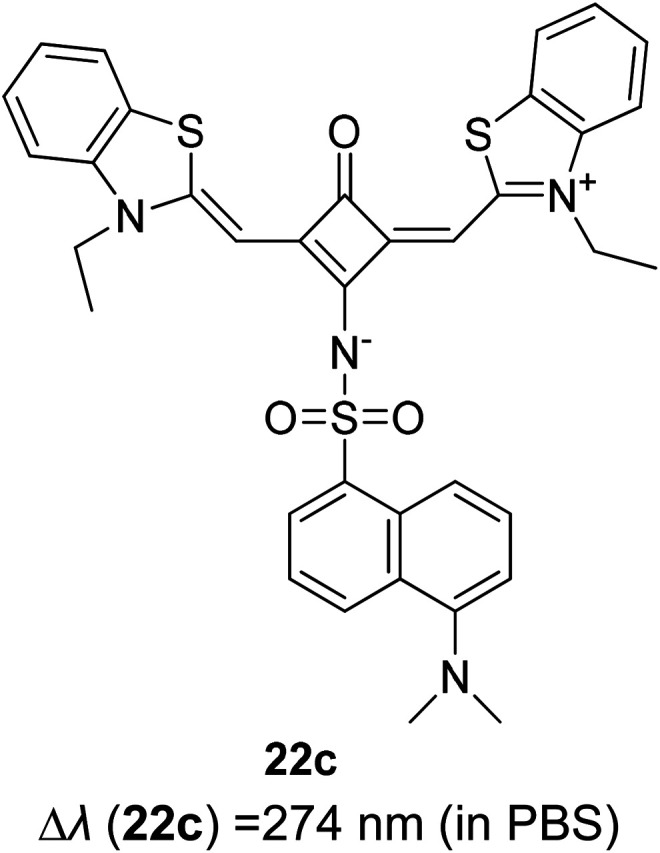	144 and 145
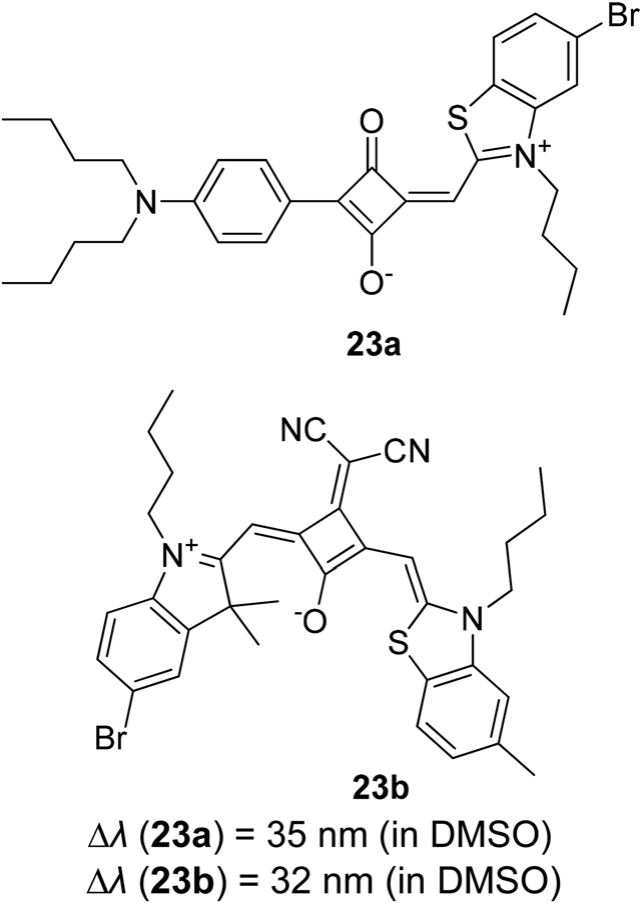	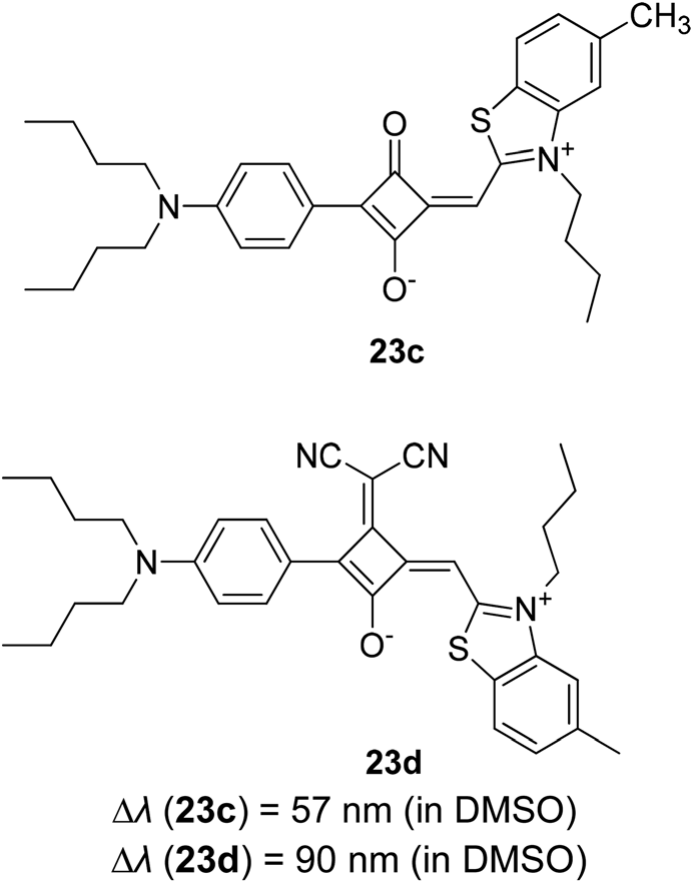		146
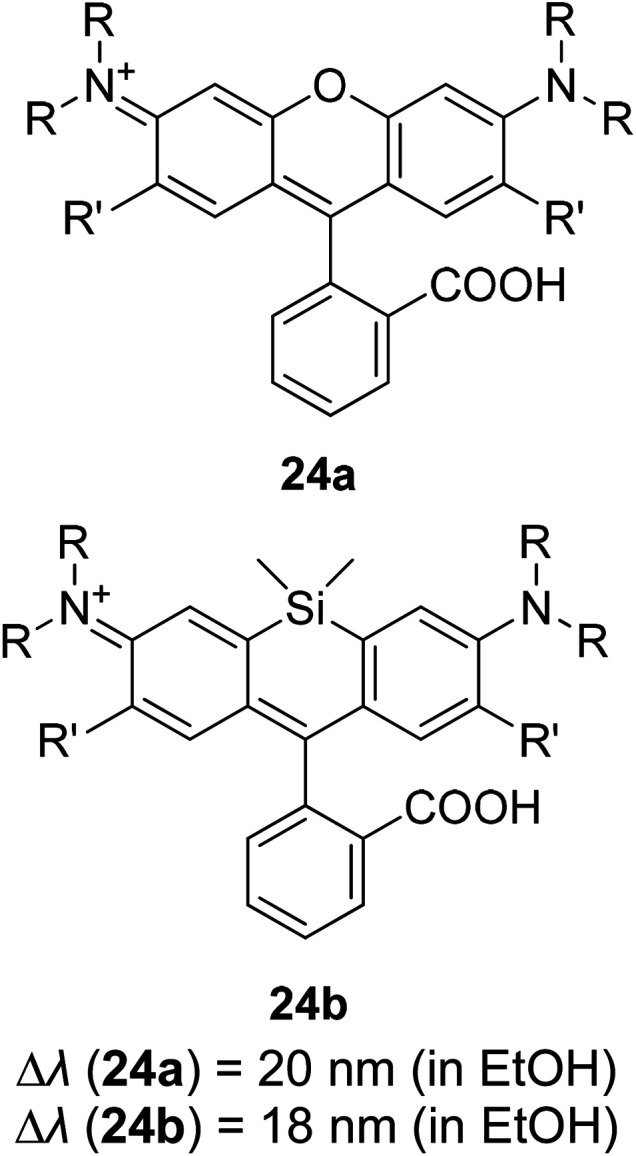		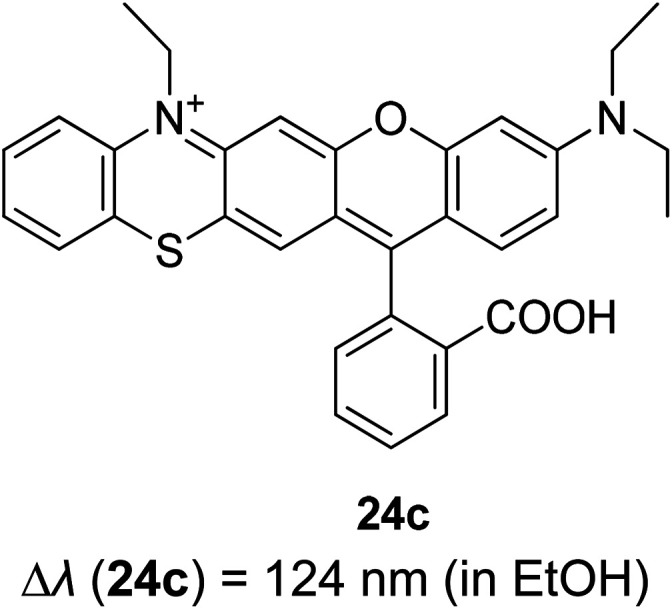	147
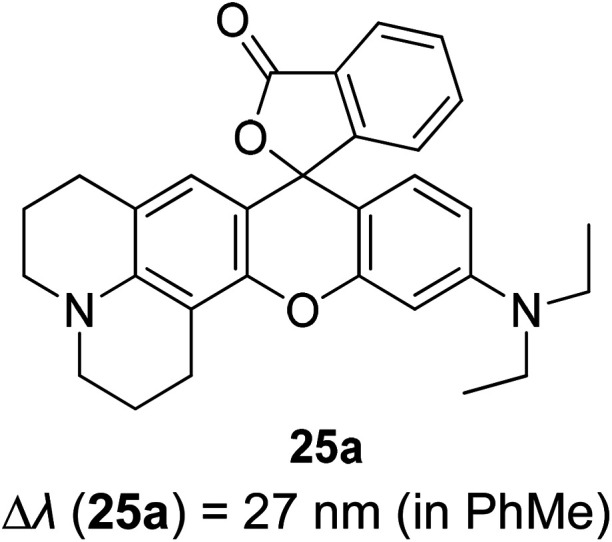	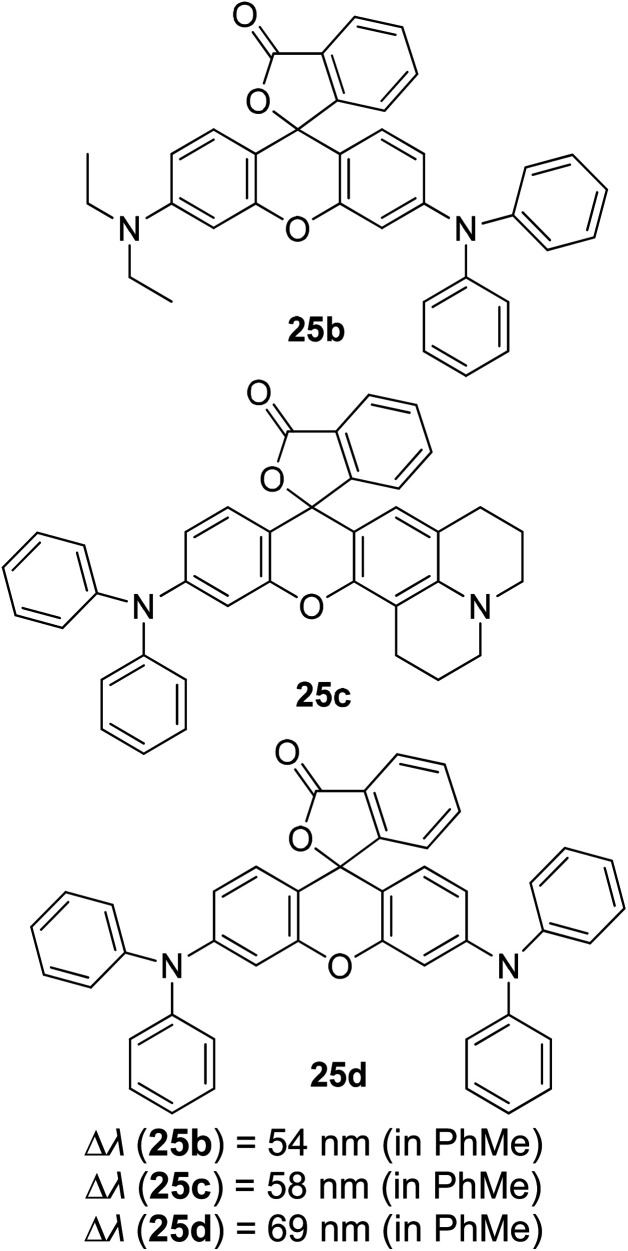		148
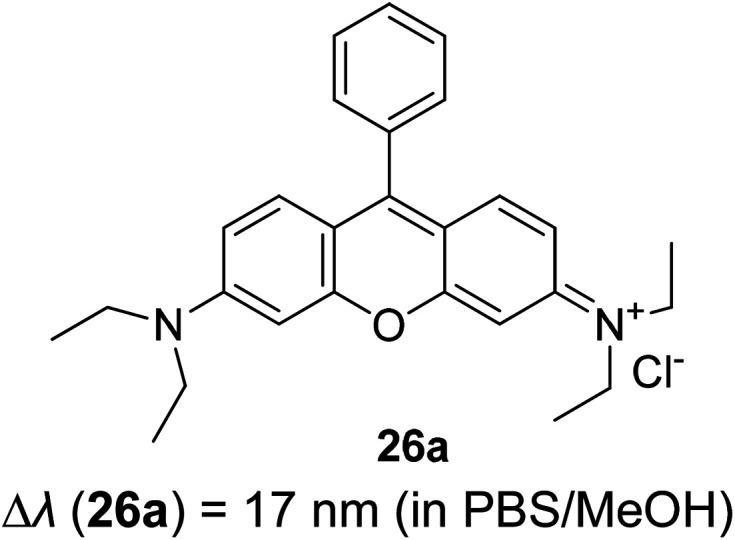	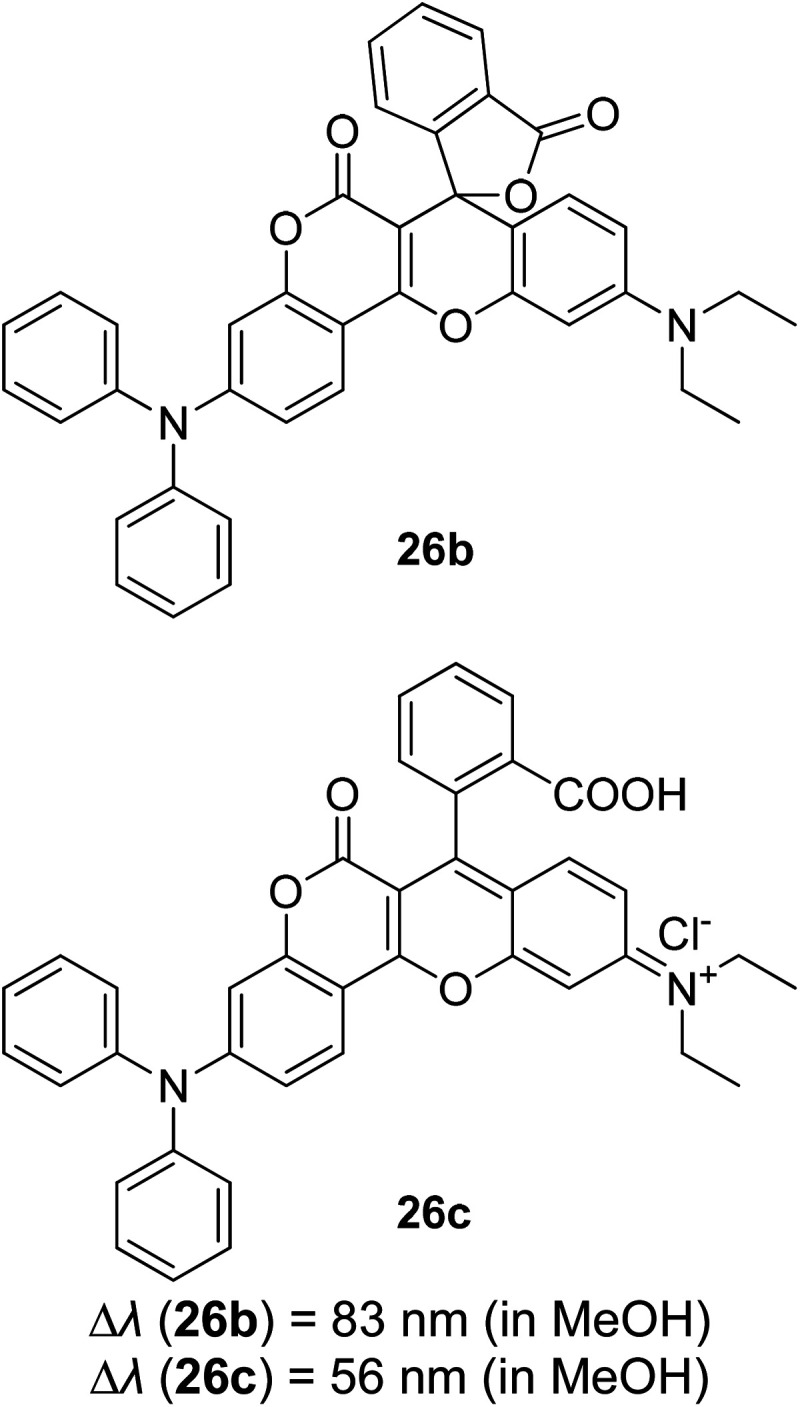	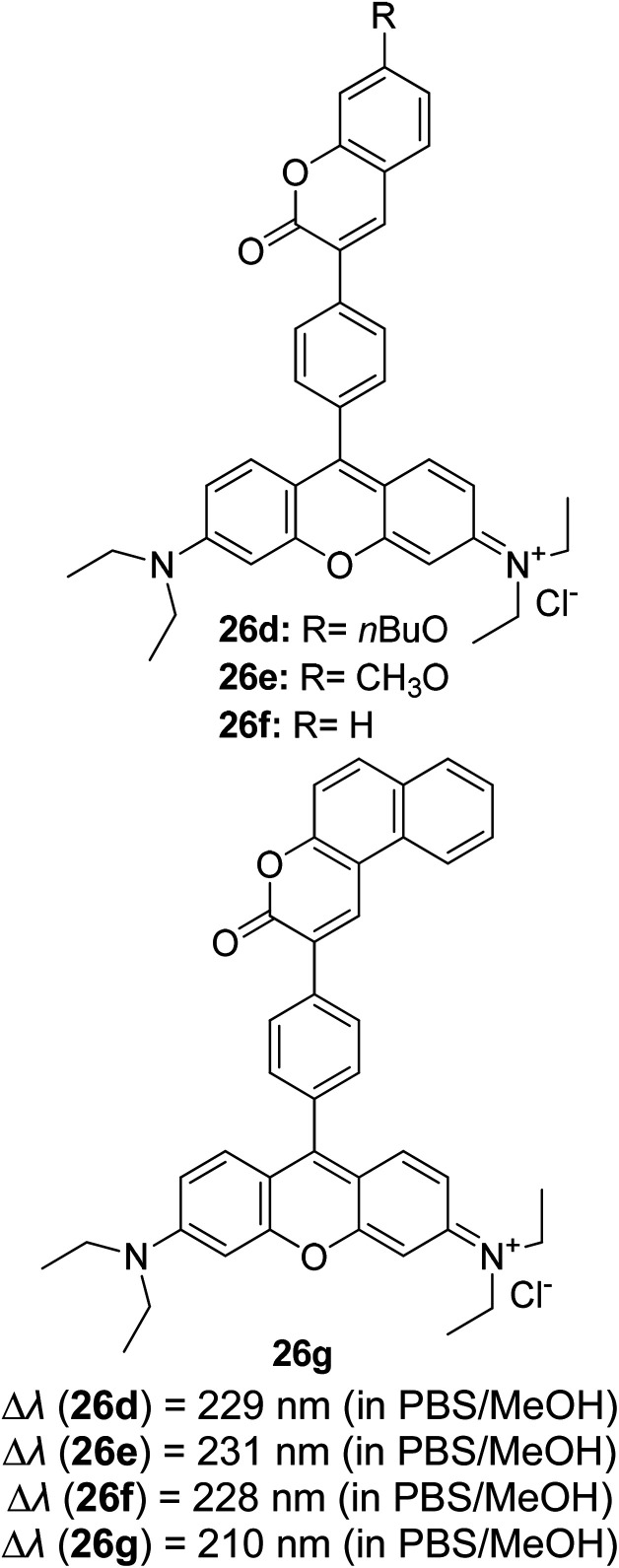	149 and 150

**Table 3:** Photoluminescence property variations for NIR molecules

Compound	*λ*_abs_ (nm)	*λ*_em_ (nm)	*Φ*_F_	*τ* (ns)	References
1a	516 (CH_2_Cl_2_)	546 (CH_2_Cl_2_)	0.660 (CH_2_Cl_2_)	—	40
	504 (MeOH)	542 (MeOH)	– (MeOH)		
	510 (DMF)	551 (DMF)	0.180 (DMF)		
1b	548 (PhMe)	578 (PhMe)	0.710 (PhMe)		
	530 (CH_2_Cl_2_)	567 (CH_2_Cl_2_)	0.640 (CH_2_Cl_2_)		
	530 (MeOH)	579 (MeOH)	0.650 (MeOH)		
	538 (DMF)	595 (DMF)	0.150 (DMF)		
1c	Refer to Table 1				
1d					
17a	502 (CH_2_Cl_2_)	512 (CH_2_Cl_2_)	0.830 (CH_2_Cl_2_)	7.60 (CH_2_Cl_2_)	138
17b	603 (hexane)	673 (hexane)	0.230 (hexane)	—	139
	605 (THF)	673 (THF)	0.450 (THF)		
	604 (CH_2_Cl_2_)	671 (CH_2_Cl_2_)	0.410 (CH_2_Cl_2_)		
	605 (CHCl_3_)	673 (CHCl_3_)	0.480 (CHCl_3_)		
	603 (MeOH)	673 (MeOH)	0.080 (MeOH)		
	608 (DMSO)	672 (DMSO)	0.130 (DMSO)		
2a	388 (CH_2_Cl_2_)	547 (CH_2_Cl_2_)	0.030 (CH_2_Cl_2_)	—	43
2b	410 (CH_2_Cl_2_)	523 (CH_2_Cl_2_)	0.550 (CH_2_Cl_2_)		
2c	353 (CH_2_Cl_2_)	544 (CH_2_Cl_2_)	0.030 (CH_2_Cl_2_)		
18a	399 (EtOH)	442 (EtOH)	0.580 (EtOH)	—	44, 151 and 152
18b	404 (EtOH)	445 (EtOH)	0.200 (EtOH)		
18c	415 (EtOH)	492 (EtOH)	0.830 (EtOH)		
18d	383 (EtOH)	540 (EtOH)	0.040 (EtOH)		
18e	406 (EtOH)	526 (EtOH)	—		
19a	777 (PBS : DMSO)	812 (PBS : DMSO)	—	—	140 and 141
19b	783 (PBS : DMSO)	814 (PBS : DMSO)	—		
19c	785 (MeOH)	807 (MeOH)	0.101 (MeOH)		
19d	784 (MeOH)	813 (MeOH)	0.117 (MeOH)		
19e	497 (CH_2_Cl_2_)	583 (CH_2_Cl_2_)	0.009 (CH_2_Cl_2_)		
19f	447 (CH_2_Cl_2_)	681 (CH_2_Cl_2_)	0.320 (CH_2_Cl_2_)		
20a	783 (MeOH)	803 (MeOH)	0.170 (MeOH)	—	142
20b	602 (MeOH)	757 (MeOH)	0.470 (MeOH)		
20c	617 (MeOH)	757 (MeOH)	0.380 (MeOH)		
21a	614 (CHCl_3_)	659 (CHCl_3_)	—	—	143
21b	575 (CHCl_3_)	606 (CHCl_3_)			
21c	665 (CHCl_3_)	869 (CHCl_3_)			
22a	724 (PhMe)	735 (PhMe)	0.073 (PhMe)	0.30 (PhMe)	144 and 145
22b	729 (PhMe)	756 (PhMe)	0.058 (PhMe)	0.65 (PhMe)	
22c	400 (PBS)	674 (PBS)	—	—	
23a	605 (DMSO)	640 (DMSO)	0.25 (DMSO)	—	146
23b	648 (DMSO)	680 (DMSO)	0.21 (DMSO)		
23c	561 (DMSO)	618 (DMSO)	0.26 (DMSO)		
23d	540 (DMSO)	630 (DMSO)	0.17 (DMSO)		
24a	553 (EtOH)	573 (EtOH)	0.530 (EtOH)	2.42 (EtOH)	147 and 153
24b	652 (EtOH)	670 (EtOH)	—	—	
24c	606 (EtOH)	730 (EtOH)	0.047 (EtOH)	6.12 (MeCN)	
25a	573 (PhMe)	600 (PhMe)	0.421 (PhMe)	—	148
25b	570 (PhMe)	624 (PhMe)	0.007 (PhMe)		
25c	581 (PhMe)	639 (PhMe)	0.002 (PhMe)		
25d	589 (PhMe)	658 (PhMe)	0.009 (PhMe)		
26a	559 (PBS : MeOH)	576 (PBS : MeOH)	0.250 (PBS : MeOH)	—	149 and 150
26b	377 (MeOH)	460 (MeOH)	0.151 (PhMe)	2.08 (PhMe)	
26c	589 (MeOH)	645 (MeOH)	—	—	
26d	353 (PBS : MeOH)	582 (PBS : MeOH)	0.270 (PBS : MeOH)	—	
26e	351 (PBS : MeOH)	582 (PBS : MeOH)	0.260 (PBS : MeOH)	—	
26f	354 (PBS : MeOH)	582 (PBS : MeOH)	0.250 (PBS : MeOH)	—	
26g	372 (PBS : MeOH)	582 (PBS : MeOH)	0.270 (PBS : MeOH)	—	

There are other factors that could affect the Stokes shift value and emission shifting of compounds, including solvation dynamic,^54,154^ pH effect,^155^ temperature,^156,157^ polymer dispersion,^158^ and others. Therefore, the incorporation of different components of Stokes shift entails a joint venture of consideration between the molecular conformation and its nanoenvironments. By establishing the fine lines of Stokes shifts, we aspire for straightforward compound classification, in the endeavor of material screening for white light generation.

## 5. Conclusion and perspectives

White light emission has been arousing great interests among researchers of different backgrounds, and plenty of research works have been accomplished to achieve emitters exhibiting large or mega Stokes shift property. Instead of discussing the plethora of methods in generating white light, we provided a focused discussion on materials designation. This review article has highlighted some recent developments of white light emitters that employ large or mega Stokes shift molecules both from organic and inorganic sources. The hallmark of large or mega Stokes shift is the fine control over the emission color through a multi-fluorophore system, mainly from red, green, and blue (RGB) emitters. This could provide a promising avenue to future optoelectronic devices and lighting applications that would unveil high color rendition, more energy efficiency, enhanced photostability and quality, and ease of device fabrication. Thus, it is necessary to understand the photophysical mechanisms that engender large or mega Stokes shift molecule. The processes that have been covered include ESIPT, ICT, excited state geometry relaxation or structure deformation, AIE, concessions between monomer and excimer, host–guest interaction and MLCT.

However, further devotion needs to be given in order to obtain large or mega Stokes shift materials with high quantum efficiency. Most of the previously reported low energy or red region emitters have considerably low fluorescence intensity due to the frantic, undesirable non-radiative decay pathways that occur extremely rapidly (10^−12^ to 10^−10^ s). In view of this, both experimental and simulation (theoretical) data of fluorescent materials from different backbones need to be extended for a deeper understanding of the electronic transitions that take place within the molecule, *i.e.*, intramolecular and intermolecular interactions based on the molecule functionalities and the applied chemical environments. The effort to harvest triplet excitons by thermally activated delayed fluorescence (TADF) is one of the progressing strategies for ameliorated quantum yield emission. Nonetheless, it comes with a competing effect that is the small gap (normally <0.2 eV) between the absorbed and emitted photons. Hence, it could be appended as a judicious approach in achieving both large or mega Stokes shift and superior fluorescence intensity. The architecture of the molecule possessing both properties that are sought after will therefore be a non-trivial task. It will especially be an arduous journey to optimize the contrasting properties to generate a pure and stable white light. To summarize, the common approaches aiming for a large or mega Stokes shift molecule are to construct a systematic material design, carrying an energy-donor–acceptor framework or/and extending the π-conjugation length of the compound. These are due to the elusive nature of the molecule's excited state upon photoexcitation. The aforementioned subtopics that revolve around the white light and Stokes shifts are in fact fascinating to further gain in-depth insight, and thus related prospects could evolve and flourish in the near future in the field of display devices, artificial lighting, bioimaging, and photosensitisers.

## Author contributions

Nadia Nabihah Mohd Yusof Chan writing – original draft, investigation, formal analysis. Azila Idris writing – review & editing, supervision, funding acquisition. Zul Hazrin Zainal Abidin supervision, validation. Hairul Anuar Tajuddin supervision, conceptualization. Zanariah Abdullah validation, project administration, resources.

## Conflicts of interest

The authors declare no conflicts of interest.

## Supplementary Material
