# Correction: White light employing luminescent engineered large (mega) Stokes shift molecules: a review

**DOI:** 10.1039/d1ra90109e

**Published:** 2021-05-14

**Authors:** Nadia Nabihah Mohd Yusof Chan, Azila Idris, Zul Hazrin Zainal Abidin, Hairul Anuar Tajuddin, Zanariah Abdullah

**Affiliations:** Department of Chemistry, Faculty of Science, University of Malaya 50603 Kuala Lumpur Malaysia azila_idris@um.edu.my; Centre for Ionics University of Malaya, Department of Physics, Faculty of Science, University of Malaya 50603 Kuala Lumpur Malaysia

## Abstract

Correction for ‘White light employing luminescent engineered large (mega) Stokes shift molecules: a review’ by Nadia Nabihah Mohd Yusof Chan *et al.*, *RSC Adv.*, 2021, **11**, 13409–13445, DOI: 10.1039/D1RA00129A.

The authors regret that Fig. 17 was incorrectly shown in the original article. The amended figure is shown below with labels **8a** and **8b** corrected to **12a** and **12b**.

**Fig. 17:**
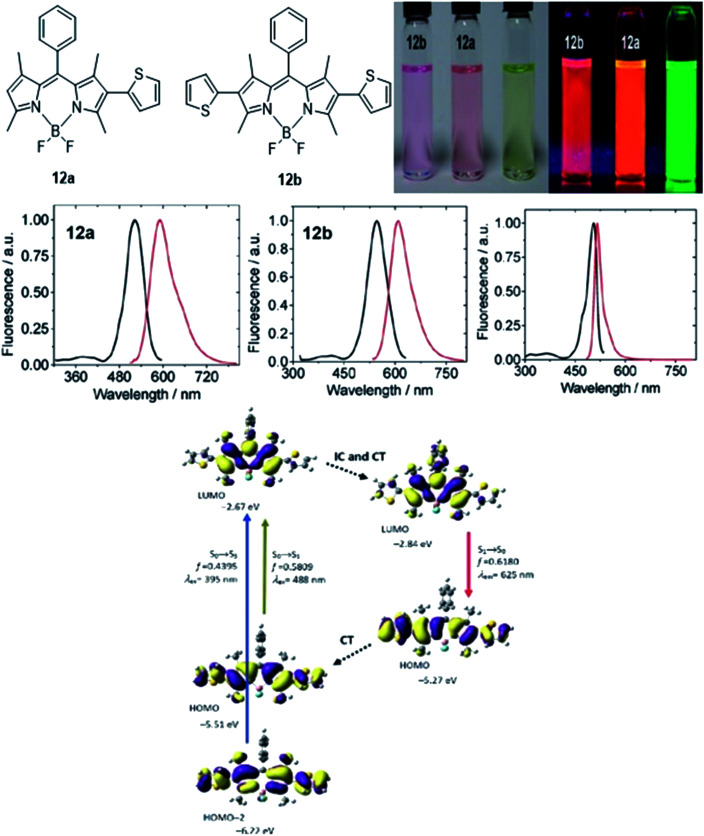
(Top) Molecular structure of 2-thienyl (**12a**) and 2,6-bisthienyl (**12b**) BODIPY derivatives and their emission colour, taken under ambient light and UV light (handheld UV lamp, 365 nm). *c* = 7.5 × 10^−6^ M (toluene, 25 °C). (Middle) Fluorescence spectra of **12a**, **12b** and unsubstituted BODIPY. (Bottom) Rationalization of the large Stokes shift of **12b**: the geometry relaxation upon photoexcitation and the frontier molecular orbitals (MOs) involved in the vertical excitation (*i.e.*, UV-vis absorption, the left two columns) and emission (right column) of **12b**. The figures were adapted from ref. 98 with permission. Copyright 2012 American Chemical Society.

The Royal Society of Chemistry apologises for these errors and any consequent inconvenience to authors and readers.

## Supplementary Material

